# Synthetic microbial communities of heterotrophs and phototrophs facilitate sustainable growth

**DOI:** 10.1038/s41467-020-17612-8

**Published:** 2020-07-30

**Authors:** Cristal Zuñiga, Tingting Li, Michael T. Guarnieri, Jackson P. Jenkins, Chien-Ting Li, Kerem Bingol, Young-Mo Kim, Michael J. Betenbaugh, Karsten Zengler

**Affiliations:** 1 Department of Pediatrics, University of California, San Diego, 9500 Gilman Drive, La Jolla, CA 92093-0760 USA; 2 Department of Chemical and Biomolecular Engineering, The Johns Hopkins University, 3400 North Charles Street, Baltimore, MD 21218 USA; 3 Department of Immunology, School of Basic Medical Sciences, Lanzhou University, 730000 Lanzhou, China; 4 National Bioenergy Center, National Renewable Energy Laboratory, Golden, CO 80401 USA; 5 Earth and Biological Sciences Directorate, Pacific Northwest National Laboratory, Richland, WA 99354 USA; 6 Center for Microbiome Innovation, University of California, San Diego, 9500 Gilman Drive, La Jolla, CA 92093-0403 USA; 7 Department of Bioengineering, University of California, San Diego, 9500 Gilman Drive, La Jolla, CA 92093-0412 USA

**Keywords:** Environmental microbiology, Computational biology and bioinformatics, Computer modelling

## Abstract

Microbial communities comprised of phototrophs and heterotrophs hold great promise for sustainable biotechnology. Successful application of these communities relies on the selection of appropriate partners. Here we construct four community metabolic models to guide strain selection, pairing phototrophic, sucrose-secreting *Synechococcus elongatus* with heterotrophic *Escherichia coli* K-12, *Escherichia coli* W, *Yarrowia lipolytica*, or *Bacillus subtilis*. Model simulations reveae metabolic exchanges that sustain the heterotrophs in minimal media devoid of any organic carbon source, pointing to *S. elongatus-E. coli* K-12 as the most active community. Experimental validation of flux predictions for this pair confirms metabolic interactions and potential production capabilities. Synthetic communities bypass member-specific metabolic bottlenecks (e.g. histidine- and transport-related reactions) and compensate for lethal genetic traits, achieving up to 27% recovery from lethal knockouts. The study provides a robust modelling framework for the rational design of synthetic communities with optimized growth sustainability using phototrophic partners.

## Abstract (web summary)

Successful application of microbial community for bioproduction relies on the selection of appropriate heterotroph and phototroph partners. Here, the authors construct community metabolic models to guide strain selection and experimentally validate metabolic exchanges that sustain the heterotrophs in minimal media.

## Introduction

Phototrophic microbial communities exhibit symbiosis between photoautotrophic and heterotrophic organisms supported primarily by solar energy and the fixation of carbon dioxide (CO_2_). This type of association dominates many biofilms, microbial mats, and lichens^1–3^, thriving in desiccation, nutrient starvation, and salinity or temperature extremes^4^. This ability to survive extreme conditions is due, in part, to division of labor and subsequent interactions between members of the community. Photoautotrophic members, classically either cyanobacteria or eukaryotic algae, convert CO_2_ into organic carbon for growth and maintenance of the heterotrophic partner(s). Exchange of these metabolites can sustain the heterotrophs under conditions devoid of any organic carbon source. In turn, the heterotrophs provide additional CO_2_, protection from environmental factors and predation, and often, a diverse array of metabolites produced by secondary metabolism^5^.

To date, understanding, engineering, and determining viable cultivation conditions for natural phototrophic communities remains challenging^6^. Thus, synthetic communities have been the primary platform for autotrophic-heterotrophic symbioses for bioenergy^7–11^, resulting in novel phototrophic systems to produce biomass and value-added compounds^7,12,13^. Synthetic phototrophic communities (SPCs) have been utilized for the production of biofuels^7^, α-amylase, and polyhydroxyalkanoates among other compounds^8,10,12^. Complex communities consisting of algae and bacteria also have potential applications in waste-water treatment^14,15^, bioremediation^16^, and as a bloom control method for phytoplankton^17^. Traditionally, microbial communities have been selected in long term adaptation and optimization experiments to define optimal culture conditions^18^. The critical challenge in synthetic community design is to maintain syntrophic interactions between members to avoid culture collapse^14,19^. Development of SPCs for bioproduction involves four critical steps: (a) strain selection, (b) screening of cultivation conditions, (c) efficient extraction of added-value products, and (d) process control and biomass recycling. The first three steps (a–c) are important drivers for implementation of successful bioproduction processes that can be optimized and guided using metabolic modeling.

Constraint-based metabolic modeling is a systems biology tool that provides a comprehensive metabolic understanding about individual microorganisms (metabolic models henceforth referred to as M-models)^20,21^ and microbial communities (community-metabolic models henceforth referd to as CM-models)^22–25^. These models account for biochemical and genomic information for an individual or community at the genome-scale. Resulting models are solved using flux balance analysis and can accurately predict thousands of functional states^24,26^. Simulations performed with CM-models describe key metabolic functions of microbial communities, defining all possible interactions among partners based on genetic and/or metabolic fitness. CM-models also enable prediction of effective culture conditions for production in robust biotechnological processes^22^.

Here, we reconstruct CM-models encompassing bacteria and fungi that are important contributors to biofuel production. The cyanobacterium *Synechococcus elongatus* PCC7942, with its high growth rate and robust metabolism, is able to synthesize hydrogen, ethanol, 2,3-butanediol, sucrose, squalene, and fatty acids among other organic chemicals from light and CO_2_^27^. For heterotrophic partners, we focus on established microbial cell factories, i.e., *Escherichia coli*, *Bacillus subtilis*, and the fungus *Yarrowia lipolytica*. CM-models for four synthetic communities based on the sucrose-secreting *S. elongatus* in pairwise combination with *E. coli (strains* K-12 and W)*, B. subtilis* str. 168, and *Y. lipolytica* Po1g are deployed. Predictions about biomass and metabolites (methanol, formaldehyde, ethanol, butanal, and succinate) production yields as well as flux distributions through the metabolic networks are experimentally validated using physiological and gene expression data. We explore metabolic fitness and genetic stability by evaluating compensatory mechanisms in resistance to knockouts of genes with lethal phenotypic traits mimicking enzymatic damage. We test predictions experimentally for ten knockout strains and explore potential production capabilities of organic chemicals, such as methanol, formaldehyde, ethanol, butanal, and succinate for all microbial communities.

## Results

### Community-metabolic models predict members’ growth rates

**Fig. 1:**
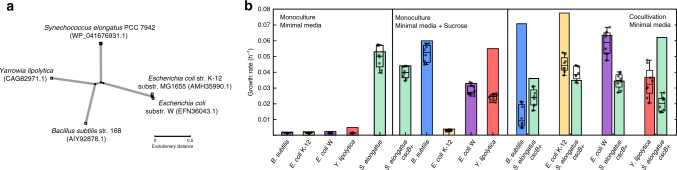
Community members and growth performance. **a** Neighbor-joining tree based of almost full-length 16S rRNA gene sequences, showing phylogenetic relationships between *S. elongatus* PCC7942 and the heterotrophic species used here. GenBank accession numbers are given in parentheses. **b** The bar plot depicts growth rates predicted using CM-models. Boxplots show experimental validation using at least six experimental replicates, the central mark indicates the median, and the bottom and top edges of the box indicate the 25th and 75th percentiles, respectively. The whiskers extend to the most extreme data points not considered outliers. The first panel shows growth in monoculture using minimal media without an organic carbon source. The second panel shows growth rates during monoculture, while using minimal media with additional sucrose. The third panel shows results obtained when combining *S. elongatus cscB*^+^ with all heterotrophs in minimal media. Source data underlying Fig. 1b are provided as a Source Data file.

CM-models are a mathematical representation of genomic and metabolic knowledge of microorganisms within a community^22,23^. These models can be used to elucidate, understand, and generate hypotheses about biological mechanisms shaping interactions between community members^22^. CM-models and M-models predict biological functions based on key substrate uptake capabilities referred to as constraints. Using the M-models of *E. coli* K-12 *i*ML1515, *E. coli* W *i*JO1366W^28,29^, *B. subtilis* str. 168, *i*YO844^30^, and *Y. lipolytica* Po1g, *i*Yali4^31^ (Fig. 1a), we reconstructed four community-metabolic models by pairing each heterotroph with the model for *S. elongatus i*JB792^32^. General properties of the community networks and M-models are shown in Table 1. The modeling compartment Shared Metabolite Pool (SMP) was manually curated in all CM-models, including experimental and genetic evidence of metabolic exchange capabilities by each community member. For example, we confirmed experimentally the consumption of 52 metabolites by *S. elongates* in monoculture. Experimental evidence, derived from high-throughput growth assays from consumption of amino acids, carbohydrates, and organic acids (BIOLOG Inc, Hayward, CA) was added into the SMP of all models. Metabolites in the SMP of all CM-models are listed in Supplementary Data 1 and simulation constraints for each CM-model are shown in Supplementary Data 2.

**Table 1:** M-model and CM-model properties.

Model features	*i*JB792	*i*ECW1372	*i*ML1515	*i*YO844	*i*Yali4	*i*CZ-Se-EcW(2157)	*i*CZ-Se-EcK-12(2152)	*i*CZ-Se-Bs(1629)	*i*CZ-Se-Yl(1686)
Genes	792	1273	1366	844	901	2157	2152	1629	1686^a^
Reactions	897	2477	2583	1250	1985	3301	3161	1921	2739
Metabolites	792	1111	1805	844	1684	2688	2547	1732	2399
Microorganism	*Synechococcus elongatus* PCC7942	*E. coli* W	*E. coli* K-12 MG 1655	*Bacillus subtilis*	*Yarrowia lipolytica*	*S. elongatus–E. coli* W	*S. elongatus–E. coli* K-12	*S. elongatus–B. subtilis*	*S. elongatus–Y. lipolytica*
Conditions	PA	A, AN	A, AN	A	A	P, A	P, A	P, A	P, A
Reference	Broddrick et al.^32^	Monk et al.^28^	Monk et al.^29^	Oh et al.^30^	Kerkhoven et al.^31^	This study	This study	This study	This study

The first row contains the names of the model. Each model can be simulated under different conditions: Phototroph models can be simulated under photoautotrophy (P), heterotrophy (H), and/or mixotrophy (M). Heterotroph models can be simulated under aerobic (A) and/or anaerobic (AN) conditions.

Organelles: *c* cytoplasm, *cx* carboxysome, *r* endoplasmic reticulum, *g* Golgi apparatus, *l* liposome, *p* periplasm, *m* mitochondria, *n* nucleus, *x* peroxisome, *t* thylakoid, and *v* vacuole.

*The consumption of ethanol was included into the *Yarrowia* model, adding genes and reactions.

We simulated CM-models based on experimental constraints, such as the sucrose secretion rate of 0.182 mmol gDW^−1^ h^−1^ by an engineered *S. elongatus* strain (*S. elongatus cscB*^*+*^)^33^. When this constraint was added into the M-model of *S. elongatus* (*i*JB792) the growth rate dropped about 16% from 0.0539 h^−1^ to 0.044 h^−1^, similar to experimental observations (0.045 ± 0.07 h^−1^). Growth rate simulations and experimental results are shown in Fig. 1b. In monoculture, M-Models successfully predicted growth phenotypes of all five microbes in minimal media. Predictions of monocultures in minimal media with sucrose were similarly successful with the exception of *Y. lipolytica*. Since, *Y. lipolytica* cannot use sucrose as its sole carbon source we used an engineered strain (*Y. lipolytica suc*^*+*^) with sucrose uptake capabilities in all the experiments shown here^34^.

Experimental validation of the CM-models yielded interesting and varied results. Predictions using the CM-models showed that *S. elongatus* (*cscB*^+^ or WT) should be able to grow at similar growth rates with *E. coli* or *B. subtilis* as in monoculture (0.039 ± 0.01 h^−1^ averaged across cocultures), but should grow faster when it is cultivated with *Y. lipolytica* (0.062 h^−1^). These expectations were validated by experiments with the exception of *S. elongates-Y. lipolytica* communities in which *S. elongatus* did not see a boost in growth but instead exhibited diminished growth, despite our simulations showing active exchange of isoleucine and other amino acids.

Synthetic phototrophic communities (SPC) of *S. elongatus cscB*^+^ with each heterotrophic partner supported growth of both community members over 72–96 h (Fig. 1b). Surprisingly, this was even the case for *E. coli* K-12, which is not able to consume sucrose. CM-model predictions of heterotrophic growth in communities with *S. elongatus* were most accurate for *E. coli* W and *Y. lipolytica* while *E. coli* K-12 and *B. subtilis* experimental growth rates were slower than predicted. Experimental growth rates of phototrophs and heterotrophs ranged between 0.04 ± 0.01 h^−1^ and 0.06 ± 0.015 h^−1^, similar to predicted growth rates of 0.05 h^−1^ and 0.07 h^−1^ (Fig. 1b). For the SPC containing *B. subtilis* we experimentally observed rapid growth of the heterotroph during the first 24 h; however, this fast growth was not sustained over the course of growth (72 h). Growth curves for all SPCs are shown in Supplementary Fig. 1.

### Predicted production capabilities underlie partner selection

**Fig. 2:**
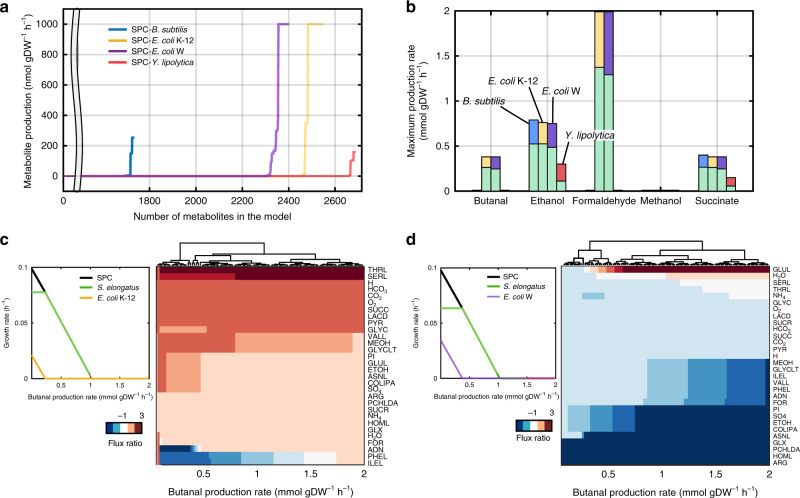
Organic chemical production by synthetic phototrophic communities. **a** Production capabilities were evaluated for all SPCs by iteratively simulating the synthesis of metabolites while maximizing for growth. Metabolite production rates were sorted and plotted for each synthetic phototrophic community (SPC). Metabolites feasible to produce were identified by having production rates over 1 × 10^−2^ mmol gDW^−1^ h^−1^. **b** Overall production capabilities for butanal, ethanol, formaldehyde, methanol, and succinate. The bar plot shows the predicted maximum production rate achieved by each SPC. The predicted abundance (growth rate) of community members is shown for *S. elongatus* *cscB*^+^(green) and for heterotrophs (blue *B. subtilis*, yellow *E. coli* K-12, purple *E. coli* W, or pink *Y. lipolytica*). Microbial communities containing *E. coli* strains showed high formaldehyde production potential. **c**, **d** Predicted growth rates and associated metabolic exchanges for the SPC composed of *S. elongatus cscB*^*+*^ and either *E. coli* K-12 (**c**) or *E. coli* W (**d**) while producing butanal. CM-models enable prediction of substrate resource allocation into growth or into target metabolites. Production of metabolites reduces the growth of community members as observed in the growth rate plots. Here, the overall growth rate of the community (black line) and each member (green *S. elongatus cscB*^*+*^ paired with yellow or purple for *E. coli* K-12 or *E. coli* W, respectively) were simulated. Simulations show that at higher butanal production rates the heterotroph will be outcompeted by the phototroph before the SPC crashes. The complete-linkage clustering based on the metabolic exchange predictions shows associations among metabolites listed on the *y*-axis and butanal production rates from left to right. Growth rate predictions were remarkably associated with a specific metabolic exchange. For example, the synthesis of butanal by the *E. coli* K-12 SPC was possible by increasing the flux exchange of metabolites such as isoleucine (ILEL), phenylalanine (PHEL), serine (SERL), and threonine (THRL), while for the *E. coli* W SPC butanal production relied heavily on L-glutamine exchange (GLUL). Metabolic exchanges of all SPCs are given in Supplementary Fig. 3.

CM-models offer a remarkably higher topological potential, achieving feasible solutions while optimizing for the synthesis of target metabolites by shifting the resources across members. CM-models contain the most comprehensive collection of metabolites that are intermediates in the synthesis of biomass or are part of the biomass compositions (e.g., carbohydrates, lipids, and proteins) of the community members. Here, we simulated production capabilities of every metabolite in the models (9369 total, Fig. 2a). Metabolite production constraints were setup as boundaries synthesize each metabolite, thus community growth simulations were calculated by maximizing the biomass growth of both community members at the same time. We considered metabolite production feasible when metabolite boundaries enabled the growth of both community members (Fig. 2a). We found community-specific production potential in all SPCs; the SPC containing *E. coli* K-12 was capable of producing the most metabolites (111 metabolites), followed by *E. coli* W (106 metabolites), *Y. lipolytica* (60 metabolites), and *B. subtilis* (39 metabolites).

We evaluated the capability of community members to secrete organic chemicals (methanol, formaldehyde, ethanol, butanal, and succinate), that are declared as primary feedstock for chemical synthesis by the Environmental Protection Agency of the United States of America. Currently these metabolites are manufactured primarily through either chemical synthesis or single-strain biorefineries^35,36^. Utilization of these metabolites accounts for 4–30% of the total production costs of feedstocks^35^. When we evaluated the maximum production capability, growth phenotypes, metabolic exchange, and yields attained with the CM-Models during the production of organic chemicals (Fig. 2b and Supplementary Fig. 2), we predicted differences in the metabolic capabilities of each SPC. For example, we found that all SPCs were theoretically able to synthesize ethanol (0.011 ± 0.01 g gDW^−1^), or succinate (0.066 ± 0.004 g gDW^−1^), but only SPCs containing *E. coli* strains were able to synthesize formaldehyde (average yield 0.017 g gDW^−1^) and butanal (average yield 0.012 ± 0.002 g gDW^−1^) (Fig. 2c, d). Metabolite productivities were compared with engineered strains under monoculture and wild-type coculture conditions, showing that cocultures are more competitive to produce succinate but not ethanol (Supplementary Data 3). Thus, using engineered strains in cocultivation could potentially achieve even higher productivities.

We found that community growth rate is driven by member-specific flux distributions and metabolic exchange, which responds differently to the requirement to produce an additional organic compound. For example, differences in the predicted flux through the SMP at each production rate of butanal were observed (Fig. 2c, d). In the community containing *E. coli* K-12, the model predicted that *S. elongatus cscB*^*+*^ would increase the flux exchange of serine and threonine over two-fold, resulting in higher secretion of these metabolites. In turn, *E. coli* K-12 would provide adenine, phenylalanine, and isoleucine at higher fluxes (Fig. 2d). Metabolic adjustments also hinted at new metabolites being exchanged (e.g., ammonium, arginine, succinate, and glyoxylate) during butanal secretion. The exchange of metabolites was activated by butanal secretion since these metabolites were not exchanged when maximizing only for biomass production. Simulations regarding the SPC with *E. coli* W showed an overall reduction of the exchange flux of all transport reactions except for glutamine. This effect was associated with depletion of the heterotrophic partner (Fig. 2d). Although *E. coli* K-12 and *E. coli* W are genetically very similar, their individual metabolic networks harbor unique metabolic capabilities. *E. coli* K-12 has 163 unique reactions and *E. coli* W has 180. While the main differences were found in alternative carbon metabolism, the metabolic networks also differed in the number of reactions in cysteine, glutamate, tyrosine, and valine metabolism, lipopolysaccharide biosynthesis, and in active transporters (Supplementary Data 4).

**Fig. 3:**
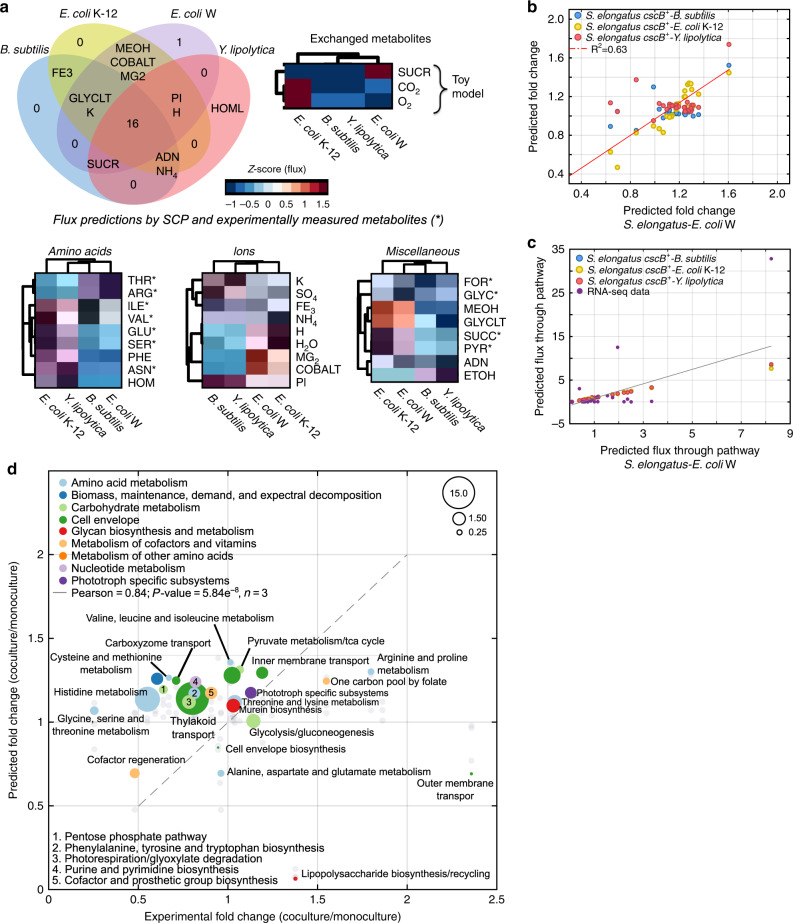
Metabolic exchange is linked to differential expression in RNA-seq data and predicted flux distributions. **a** The Venn diagram shows the predicted metabolic exchange for all synthetic phototrophic communities. Metabolites highlighted in bold represent the 16 (e.g., ASNL, GLUL, and ILEL) metabolites shared by all synthetic communities. Besides expected metabolites to be exchanged (CO_2_, O_2_, and sucrose), simulations show mutual transfer of amino acids, ions, and other metabolites. Marked metabolites (*) were identified in supernatant samples using target metabolomics (Tables 2, 3). **b** *S. elongatus cscB*^+^ fold-change analysis. Correlation between predicted subsystems fold-change of *S. elongatus* under cocultivation with *E. coli* W (*x*-axis) versus predicted fold-change when cultivated with *B. subtilis* (Pearson = 0.49, *p*-value = 0.011, *n* = 3, blue dots), *E. coli* K-12 (Pearson = 0.93, *p*-value = 9.1e−^12^, *n* = 3, yellow dots), and *Y. lipolytica* (Pearson = 0.33, *p*-value = 0.009, *n* = 3, red dots). **c** Predicted flux for each pathway in *S. elongatus* cocultured with *E. coli* W (*x*-axis) and other heterotrophic partners (*y*-axis) including *B. subtilis*, *E. coli* W, and *Y. lipolytica*. Flux evaluated by RNA-seq of S. elongatus cocultured with *E. coli* W (purple) demonstrates validation of flux prediction (Pearson = 0.82, *p*-value = 1.02e^−7^, *n* = 3). **d** Fold-change of RNA-seq data and predicted flux distributions under mono- and coculture was calculated and normalized at the pathway level. Expression data were obtained for the SPC containing *E. coli* W. Bubble size represents the median of expression burden and flux burden by pathway. Predicted fold-change for the microbial communities containing *B. subtilis*, *E. coli* W, and *Y. lipolytica* is shown in light gray dots.

Community members interact through adjusting their fluxes associated with metabolic exchange and/or with specific metabolic pathways. In addition to CO_2_ and O_2_, the models predicted a wide array of other metabolites, including alcohols, organic acids, and amino acids, would be exchanged in order to maximize growth. Overall, the CM-models identified 16 exchanged metabolites in all SPCs (Fig. 3a, Venn diagram). These included metabolites supplemented in the culture medium as well as biologically produced metabolites, such as threonine, isoleucine, and pyruvate (highlighted in bold in Fig. 3). Additionally, most of the SPCs exchanged metabolites specific for a particular SPC, such as homoserine for the community composed of *S. elongatus* and *Y. lipolytica*, and arginine for *S. elongatus* and *E. coli* W. Predicted fluxes associated with metabolic exchanges were clustered into three groups, i.e., ions, amino acids, and miscellaneous (organic acids, overflow metabolism and carbohydrates). We predicted that the SPC containing *E. coli* K-12 was the most efficient in exchanging amino acids based on its total metabolite exchange flux, e.g., isoleucine, valine, glutamate, serine, and phenylalanine (Fig. 3a, clustergram). Similar predictions about higher fluxes were obtained for ions and miscellaneous metabolites. The SPC with the second highest exchange fluxes was *S. elongatus* cscB+ and *E. coli* W, which was characterized by high exchanges of threonine, arginine, and isoleucine. This SPC was the most efficient in activating exchange reactions containing magnesium and cobalt. The total fluxes in the SPCs containing *B. subtilis* or *Y. lipolytica* were lower in comparison to fluxes of SPCs containing *E. coli*. The *B. subtilis* and *Y. lipolytica* containing SPCs clustered together and exhibit remarkably different flux profiles compared to *E. coli* K-12 (see also Supplementary Figs. 3, 4).

Interestingly, the SPC composed of *S. elongatus* cscB^+^ and *E. coli* K-12, which is unable to grow with sucrose, was predicted to thrive in minimal media devoid of any organic carbon source. These predictions were validated experimentally and we identified *E. coli* K-12 as the most suitable heterotrophic partner for *S. elongatus*, exhibiting the highest growth phenotypes. We therefore characterized the metabolic and genetic interactions of *S. elongatus* *cscB*^+^ and *E. coli* K-12 in greater detail.

### Metabolic profiles give insight into microbial interactions

Metabolic interactions were experimentally characterized using Nuclear Magnetic Resonance (NMR) and Gas Chromatography-Mass Spectrometry (GC-MS). Metabolic profiles for 34 metabolites were measured from biomass samples and culture medium supernatants under mono- and coculture conditions. Experimentally determined metabolic profiles validated the exchange of predicted metabolites. NMR and GC-MS analysis of intracellular (Table 2) and extracellular (Supplementary Data 5) metabolites for the SPC of *S. elongatus cscB*^+^ and *E. coli* K*-*12 along with monoculture profiles was performed. Sucrose remained constant in the extracellular medium of *S. elongatus cscB*^+^ monoculture and the *S. elongatus cscB*^*+*^-*E. coli* K-12 coculture due to the lack of a sucrose transporter in this *E. coli* strain. Other metabolites detected in the extracellular broth of this SPC (*S. elongatus cscB*^*+*^-*E. coli* K-12) were pyruvate, methanol, formate, acetate, and high concentrations (13.7 mM) of ethanol, suggesting that these are also available for metabolite exchange. These metabolites were often lower in coculture than in monocultures, providing further support of this exchange.

**Table 2:** NMR data for extracellular metabolite concentrations.

Metabolite	Metabolite ID	*E. coli* K-12 (μM)	Coculture (μM)	*S. elongatus* (μM)
2-oxoglutarate	akg	404.200	0.000	0.000
3-phosphoglyceric acid	g3pg	NF	NF	NF
4-hydroxybenzoate	4hbz	12.800	0.000	0.000
Acetate	ac	424.000	2.300	3.400
Acetone	aact	7.300	4.400	7.200
Adenine	ade	0.000	0.000	0.000
Citric acid	cit	NF	NF	NF
D-arabinose	arab	NF	NF	NF
D-fructose	fru	NF	NF	NF
D-fructose-6-phosphate	f6p	NF	NF	NF
D-glucose	glc	0.000	0.000	0.000
D-glucose-6-phosphate	g6p	NF	NF	NF
D-mannitol	mnl	NF	NF	NF
D-ribose	rib	NF	NF	NF
D-ribose-5-phosphate	r5p	NF	NF	NF
Ethanol	etoh	13.200	13.700	18.600
Formate	for	1343.200	6.500	43.300
Glycerol	glyc	NF	NF	NF
Glycine	gly	NF	NF	NF
Isobutyrate	but	2.300	0.000	0.000
L-aspartic acid	asp	0.000	0.000	0.000
L-glutamic acid	glu	0.000	0.000	0.000
L-serine	ser	NF	NF	NF
L-threonine	thr	0.000	0.000	0.000
L-valine	val	0.000	0.000	0.000
Methanol	meoh	1.600	6.700	5.400
Pantothenate	pnto	5.800	0.000	0.000
Proline	pro	NF	NF	NF
Propylene glycol	glyclt	4.700	0.000	0.000
Pyruvate	pyr	0.000	0.000	2.600
Succinate	succ	5.400	0.000	0.000
Sucrose	sucr	903.500	86.000	101.500
Tyrosine	tyr	0.000	0.000	0.000
Lactate	lac	0.000	0.000	0.000

Standard error of all measurements is below 10% of the average observed concentration. Measurements obtained from independent samples.

*NF* not found. Predicted metabolites not detected in NMR and GC-MS: L-arginine, L-isoleucine, L-phenylalanine, and homoserine.

Our computational studies suggested optimized growth should include the exchange of a number of additional metabolites, especially amino acids (Fig. 3a). In order to determine if these amino acids and other metabolites were present in cells, we evaluated intracellular metabolites in *S. elongatus cscB*^*+*^ and *E. coli* K-12 using GC-MS. Some intracellular metabolites, including the amino acids glutamate and aspartate and the dipeptide glycylproline (precursor of serine and threonine synthesis) accumulated to higher relative levels in *S. elongatus* compared to *E. coli*. Other metabolites, such as malonate, were detected intracellularly as well. GC-MS analysis (Table 3) confirmed the high intracellular levels of glutamate and aspartate as well as other amino acids in *S. elongatus*, and also revealed high intracellular levels of glycerol and glycine in *S. elongatus* relative to its *E. coli* K-12 partner. Interestingly all these metabolites were confirmed experimentally to be effectively transported into the cytoplasmic space of *S. elongatus* and *E. coli* K-12 (Supplementary Data 1). Overall, our NMR and GC-MS analysis revealed a different intracellular metabolic fingerprint for the cyanobacterium as compared to its heterotrophic partner. This provides further evidence that phototrophic communities can be advantaged through division of labor using interwoven metabolic networks, especially in alleviation of partner-specific bottlenecks.

**Table 3:** Intracellular metabolite Z-score comparisons from GC-MS.

Metabolite	Metabolite ID	*E. coli* K-12 (Z-score)	*S. elongatus* (Z-score)
2-oxoglutarate	akg	2.077	0.085
3-phosphoglyceric acid	g3pg	−0.391	2.258
4-hydroxybenzoate	4hbz	−0.535	−0.418
Acetate	ac	NF	NF
Acetone^a^	aact	1.943	−0.917
Adenine	ade	1.575	1.072
Citric acid	cit	−0.428	−0.963
D-arabinose	arab	1.759	−0.591
D-fructose	fru	0.953	−0.546
D-fructose-6-phosphate	f6p	2.069	0.571
D-glucose	glc	2.212	−0.343
D-glucose-6-phosphate	g6p	1.618	1.297
D-mannitol	mnl	2.268	−0.378
D-ribose	rib	2.262	−0.363
D-ribose-5-phosphate	r5p	−0.220	2.262
Ethanol	etoh	NF	NF
Formate	for	NF	NF
Glycerol	glyc	−0.372	1.599
Glycine	gly	0.178	2.199
Isobutyrate	but	2.268	−0.378
L-aspartic acid	asp	0.277	2.173
L-glutamic acid	glu	0.210	2.192
L-serine	ser	1.390	−0.585
L-threonine	thr	−0.370	2.264
L-valine	val	NF	NF
Methanol	meoh	NF	NF
Pantothenate	pnto	0.951	−0.410
Proline	pro	−0.378	2.268
Glycolate^b^	glyclt	0.135	0.784
Pyruvate	pyr	2.249	−0.142
Succinate	succ	2.263	−0.429
Sucrose	sucr	−0.179	−0.546
Tyrosine	tyr	0.247	1.936
Lactate	lac	2.248	−0.196

Z-score was determined using three independent biological replicates. Standard error of all measurements is below 10% of the average observed concentration.

*NF* not found.

Metabolite precursor found:

^a^Acetoacetate.

^b^Propylene glycol.

### Simulated pathway fluxes suggest network bottlenecks

CM-Models not only unravel metabolic exchange but can also decipher alterations in the activity of complete pathways. Shifting from mono- to coculture conditions triggered changes across fluxes in multiple pathways of the entire network. Our predictions suggested that these metabolic changes occur in all SPCs studied here. The predicted fold-change (coculture/monoculture) of subsystem fluxes in all SPCs were remarkably correlated (Pearson correlation, average *R*^2^ = 0.63) (Fig. 3b, c). Similarly, the predicted flux through pathways were well correlated across all SPCs and significantly correlated with *S.elongatus* and *E.coli* W expression data (Pearson = 0.82, *p*-value = 1.02e^−7^).

Predicted flux differences between mono- and coculture conditions were identified in transport reactions and metabolism of pyruvate, purine, pyrimidine, alanine, aspartate, and glutamate in both members of the SPC (Fig. 3d). Furthermore, flux through member-specific pathways, such as photosynthesis, carbon fixation, and biosynthesis of plastoquinol, phylloquinone, and carotenoids, significantly changed between mono- and coculture conditions. Measured expression data of the SPC containing *S. elongatus* and *E. coli* W showed significantly differential expression (two sided *t*-test, *n* = 3, *p*-value < 0.05) of genes associated with alanine, aspartate, and glutamate metabolism, arginine and proline metabolism, the pentose phosphate pathway, phototroph-specific metabolism, purine and pyrimidine metabolism, and transport reactions as predicted (Supplementary Fig. 5 and Supplementary Data 7).

We quantified and compared expression and fluxes by pathway, identifying potential bottlenecks that if relieved could improve biomass yields. For example, predicted flux and observed expression data associated with transporters located in the thylakoid as well as histidine metabolism in the cytoplasm were highly activated, making up around 50% of the total expression (RNA-seq reads) and flux (mmol gDW^−1^ h^−1^) across the entire metabolic network. Histidine metabolism is linked with the pentose phosphate pathway and alanine, aspartate, and glutamate metabolism, encompassing the synthesis of several glutamate precursors. Even though we could not quantify/detect intracellular accumulation of histidine experimentally, glutamine, a histidine precursor, was accumulated in both *S. elongatus* (9.9 mM) and *E. coli* K-12 (2.1 mM). This metabolite was not found in the supernatant of any mono- or cocultures. Overall, CM-models are a reliable tool to identify functional states in association with growth phenotypes, offering details of cellular communication and potential network bottlenecks limiting microbial interactions.

### SPCs are more resistant to lethal traits than monocultures

Tolerance to inhibitors is one of the main challenges for strain engineering of monocultures. Often growth rates and productivities are limited by the accumulation of toxic intermediates (e.g., end-products and/or metabolic intermediates) that can harm productivity or lead to culture collapse^37^. These inhibitions can affect metabolism at the reaction or pathway levels^38^. To probe the ability of SPCs to cope with enzymatic inhibition, we simulated single gene deletions of all genes in the CM-models (7624 total).

**Fig. 4:**
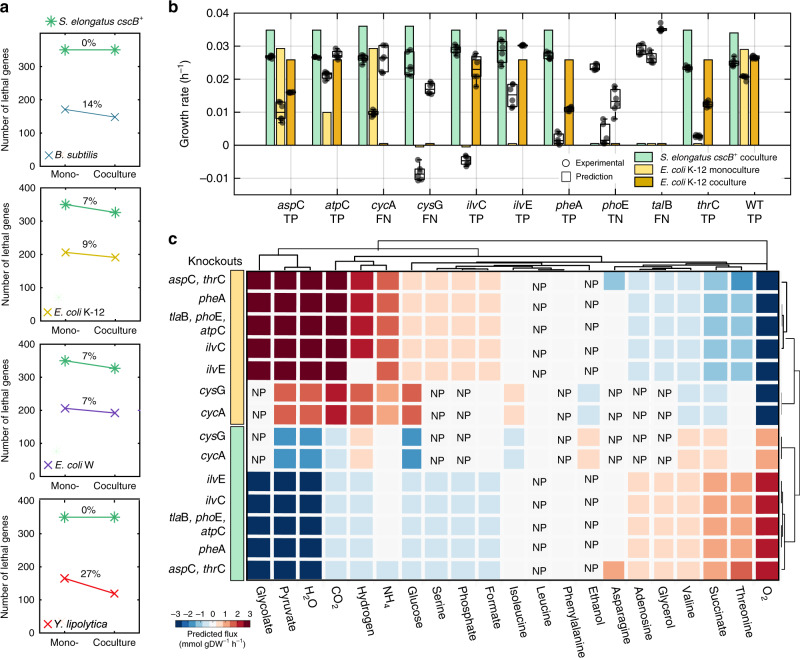
Community fitness compensates for lethal phenotypic traits by tuning metabolic exchange. **a** Simulations about resistance to lethal phenotypes across community members. The percentage of knockouts (KOs) with lethal phenotypes was estimated for both community members using the difference of lethal KOs among monoculture and coculture conditions. The percentage reduction is shown for *S. elongatus* (green) and for the heterotrophs. The communities can reduce their total lethal phenotypic outcomes by 5–9%. However, the heterotrophic partner benefited disproportionately, reducing the number of lethal traits by at least 7% for *E. coli* W and up to 27% for *Y. lipolytica*. The percentage of lethality reduction was determined using the number of genes that are lethal in monoculture vs coculture. The complete dataset is given in Supplementary Data 8, 9. **b** Experimental validation of selected genes with resistance phenotypes. Six *E. coli* K-12 mutant strains with lethal phenotypes (*ilv*C, *cys*G, *ilv*E, *phe*A, *pho*E, and *thr*C) and four with nonlethal phenotypes (*asp*C, *cyc*A, *tal*B, and *atp*C) were experimentally tested under monoculture and coculture conditions. Predicted growth phenotypes are shown with bars and experimental results with boxplots. In boxplots the central mark indicates the median, and the bottom and top edges of the box indicate the 25th and 75th percentiles, respectively. Benchmarking of the models is achieved by comparing experimental and predicted outcomes as true positive (TP), true negative (TN), false positive (FP), and false negative (FN). **c** Predicted metabolic exchange shifts in accordance with the KO studied. Complete-linkage clustering shows metabolic exchange associations by genes (*y*-axis) and metabolites (*x*-axis). The effect of each KO is shown for *S. elongatus* (green) and *E. coli* K-12 (yellow). Flux distributions were normalized by using the flux distribution obtained under wild-type conditions for both members. Predicted metabolic exchange of *tal*B, *pho*E, and *atp*C knockouts is represented in one row as the shifts were identical. Similarly, results were found for the metabolic exchange of KOs *asp*C and *thr*C. NP stands for metabolite not predicted to exchange under given condition. Source data underlying Fig. 4b are provided as a Source Data file.

Model simulations showed that members of microbial communities can efficiently compensate for some metabolic activities, overcoming up to 27% of phenotypic traits lethal to one member, allowing the damaged participant to thrive in the community though they could not in monoculture. For example, during monoculture growth of *S. elongatus cscB*^+^, 350 genes are essential, meaning if they are knocked out *S. elongatus cscB*^*+*^ cannot grow in minimal media. This is in contrast to *S. elongatus cscB* + grown in communities where the number of lethal deletions falls to 327 or 326, depending on the cocultivation partner (Fig. 4a). This equates to a 7% increase in community resilience in the face of gene knockouts as a proxy for inhibited genes. So despite *S. elongatus cscB*^+^ being the growth-sustaining microorganism in the community, our predictions showed that the presence of *E. coli* strains could compensate for up to 7% of lethal knockouts in *S. elongatus cscB*^*+*^.

We used these genome-scale model simulations to guide the selection of ten *E. coli* K-12 knockout (KO) mutants with variable growth phenotypes in monoculture versus coculture conditions to test experimentally. Six of those mutants were predicted to not grow in monoculture and were associated with amino acid metabolism (*ilv*C, *ilv*E, and *phe*A), cofactor and prosthetic group biosynthesis (*cys*G and *thr*C), and inner membrane transport (*pho*E). Of these, most strains died in monoculture in minimal media as expected and only the strain lacking *ilvE* grew in monoculture under our cultivation conditions (Fig. 4b). Four strains with non-essential genes knocked out were associated with amino acid metabolism (*asp*C), oxidative phosphorylation (*atp*C), the pentose phosphate pathway (*tal*B), and inner membrane transport (*cyc*A) (Fig. 4b, Supplementary Data 8, 9). All of these strains grew successfully in monoculture albeit with grow rates that varied significantly with respect to the predicted rates, since we used uptake rates determined under wild-type conditions to constrain the CM-model.

Growth of *E. coli* strains missing *ilv*C*, cys*G*, phe*A*,* and *thr*C was rescued by *S. elongatus cscB*^+^ as predicted for the strains *ilv*C *and phe*A (Supplementary Data 9). The genetic damage in the heterotrophic partner did not significantly affect the growth rate of the phototroph experimentally (Fig. 4b, two sided *t*-test, *n* = 4, *p*-value > 0.05). The model demonstrated good accuracy for growth/no-growth prediction of the ten assayed KOs. Matthews correlation coefficient (MCC) values were estimated for each condition, by comparing experimental and predicted outcomes (Supplementary Data 9). Across KOs, the highest prediction accuracy was observed for *E. coli* K-12 monoculture (0.90), followed by *S. elongatus cscB*^+^ coculture (0.7) and *E. coli* K-12 coculture (0.7); the positive predicted values ranged from 0.83 to 1 for all conditions. Selected strains were also cultured in rich medium (80% Luria-Bertani (LB) + 20% BG-11). We observed that all KOs were rescued in monoculture. Interestingly, the phototrophic communities maintained syntrophic interactions despite the capability of *E. coli* to thrive in rich medium. Overall, the growth rates of community and monoculture increased up to 41% while cultured in rich medium (see Supplementary Fig. 6).

Predicted metabolic exchange was highly reciprocal among community members, meaning that the secretion flux was identical to the uptake flux by the partner for most metabolites under optimal conditions. However, simulations also showed additional secretion of metabolites by *E. coli* K-12, such as lactate and citrate for all KO strains. Resistance to lethal phenotypic traits is associated with metabolite-tuning and metabolite-flux-activation under specific conditions. For example, simulations of the *E. coli* K-12 *phe*A gene KO promoted increased uptake of O_2_ from *S. elongatus*, while *E. coli* K-12 increased uptake of threonine, succinate, valine, glycerol, adenosine, phenylalanine, and asparagine while secreting CO_2_, glycolate, pyruvate, glucose, serine, formate, isoleucine, and deactivating the exchange of leucine and ethanol (Fig. 4c).

This advanced community modeling tool gives insight into stability of these cultivation platforms, namely through enhanced tolerance to inhibition through offsetting lethal traits among community members. Future network discoveries of individual microorganisms will continue improving the definition of constraints as well as the predictive power of M-models and eventually of CM-models. Such discoveries will highlight areas of interest that may be responsible for current mismatches between predictions and experimental results.

## Discussion

Emergent metabolic modeling tools have enabled significant progress toward understanding interactions in microbial communities. This achievement, together with advances in various omics methodologies, has opened the possibility of switching from biorefineries consisting of monocultures to multi-organismal cell factories^22,25^. Currently, a number of challenges to multi-organismal cell factories remain, such as high sensitivity to cultivation collapse under unexpected conditions and/or for unknown reasons^7,14,26^. Utilization of sustainable microbial communities (e.g., SPCs) has resulted in a promising approach to overcome current limitations^7,10,11^. Furthermore, the use of both synthetic and natural phototrophic communities allows for the utilization of renewable resources for sustainable production^39^, maintaining diverse and robust microbial interactions, reducing overall cost of feedstocks and minimizing transport logistics^35^. We explored the potential of photosynthesis-based processes as sustainable growth. Simulations predicted different growth phenotypes depending on the heterotrophic partner (*B. subtilis, E. coli* K-12, *E. coli* W, or *Y. lipolytica*). The SPCs containing *E. coli* improved community growth effectively by exchanging metabolites (e.g., asparagine, glutamine, phenylalanine, pyruvate, serine, succinate, and valine, among others, Figs. 2, 3) to take maximum advantage of supplied nutrients and light for the production of organics, as predicted (Fig. 2b, d). Meanwhile, SPCs containing *B. subtilis* or *Y. lipolytica* did not grow experimentally as predicted, possibly due to the high sensitivity of amino acids to folding events of metal transporters^40^, deactivation of hexose transporters in *Y. lipolytica*^41^, as well as to the presence of reactive oxygen species^7,8^ potentially affecting growth of *B. subtilis*. Furthermore, sugar assimilation in *Y. lipolytica*, including the uptake mechanism, metabolic pathways, and regulatory mechanisms, is currently poorly understood^26,41^.

Predicted member fitness depends on specific key functions that maintain and enhance the community performance of the SPC, especially in pathways regarding arginine and proline metabolism, citric acid cycle, one-carbon and folate metabolism, and pyruvate metabolism (Fig. 3b). Accurate prediction of metabolic adjustment and fitness improvement in coculture conditions offers a portrait of integral usage of light, CO_2_, and O_2_ in addition to other metabolites, such as alcohols, organic acids, and amino acids that are naturally produced during cultivation and can enhance community performance. Synthesis of metabolites associated with overflow and secondary metabolism (e.g., formate, ethanol, and acetate, Supplementary Figs. 3, 4) is prevented as much as possible in monocultures^42^ or controlled by the energetic state of cells^43^. However, for microbial communities, the active exchange of these overflow metabolites maintains and promotes the growth of community members. Certain metabolites predicted to be part of the metabolic exchange in this study have previously been reported in experimental studies. For example, pyruvate and glycerol, which were secreted by our SPCs (Supplementary Figs. 3, 4), were also detected to be secreted by the phototroph *Auxenochlorella protothecoides*, and incorporated by heterotrophic partners^15^. In turn, the heterotrophs provided vitamin precursors, increasing the growth rate and lipid productivity of the phototroph^15^.

Constraint-based modeling of microbial communities is a promising approach for developing, and controlling robust and stable multi-organismal biotechnological processes. In particular, manually curated CM-models, which compile knowledge about network connectivity, cell communication, and overall function in great detail^22,24,44^. Our CM-model for *S. elongatus* and *E. coli* K-12 predicted that activation/deactivation of transport activity associated with the thylakoid and histidine metabolism can potentially relax metabolic bottlenecks. Simulations were validated by transcriptional and metabolomic analyses of the photobiont while cocultivated with *E. coli*. Both flux predictions and expression data showed significant changes in core genes of energy metabolism, carbon fixation, and cofactor and vitamin synthesis. These results are in sync with a previous expression analysis for the SPC of *Thermosynechococcus elongatus* BP-1 and *Meiothermus ruber* strain A, in which significant differential expression was observed in phototroph-specific pathways^45^.

Mathematical models of heterotrophic-autotrophic communities have been previously published, encompassing a limited number of parameters, such as inorganic carbon, nitrogen, phosphorous, and light uptake rates^46,47^. However, CM-models provide insight into diverse environmental and genetic stages (here we used 9369 metabolites associated with 11,129 reactions and 7627 genes total), as well as into the interactions among stages for each microbial community. The broader potential of CM-model simulations enables quantitative insights into metabolic exchange and pathway fitness. Other approaches, e.g., one developed for the diatom *Phaeodactylum tricornutum* and the bacterium *Pseudoalteromonas haloplanktis*^48^, using a combination of kinetic and constraint-based models, require determination of specific exchange rates (e.g., glutamine, threonine, asparagine, serine and aspartate) for simulation. Experimental quantification of these parameters can be challenging even for monocultures^23^, limiting the simulation capabilities of these multiscale models. The shared metabolite pool provides strong versatility to CM-models to determine production capabilities of each microbial community as well as to track quantitative biochemical fluxes and therefore specific resources used to produce metabolites of interest.

Usually, inhibitory effects can diminish the growth of microorganisms in their natural environment and in many industrial applications^49,50^, but our modeling predictions for all SPCs indicated that cocultivation can help circumvent potential inhibitory effects by redirecting flux and metabolic exchanges between members (Fig. 4). An experimental study of the SPC composed of the diatom *P. tricornutum* and the bacterium *Alteromonas macleodii* demonstrated that the KO of nitrate reductase in the heterotrophic partner was compensated by the diatom. This phenotype was attributed to possible metabolic exchange of other nitrogenized compounds^51^. Other studies have also linked nitrate or ammonium availability with a defined type of microbial interaction (e.g., competition, mutualism, or amensalism)^21,22,52^. Additionally, experimental results using transposon sequencing (TN-seq) applied to microbial communities have shown differences in gene essentiality between mono- and coculture conditions^53–55^. This effect, described as community dependent essentiality, resulted in similar phenotypic outcomes as generated by our metabolic modeling approach. For example, TN-seq data of *E. coli* collected during cocultivation with *Hafnia alvei*, *Geotrichum candidum*, and *Penicillium camembertii*^54^ showed similar gene essentiality (*asp*C, *ilv*E, *ilv*C, *thr*C, *cor*A, *phe*A, and *thr*C) as our SPC of *S. elongates* and *E. coli*. Interestingly, gene essentially of the ATP synthase/thiamin triphosphate synthase complex *(atp*C in our SPC) was also observed in a different coculture composed of *Staphylococcus aureus* and *Pseudomonas aeruginosa* (*atp*D)^53^ hinting at commonalities in the evolution of diverse microbial communities that could be independent of the members. This intriguing hypothesis requires additional experimental confirmation.

To date, a lack of detailed knowledge about microbial communities has limited our ability to predict their responses and redirect their activity for improved bioproduction^23,56^. However, the development of innovative modeling tools that enable quantitative identification of potential metabolic drivers among a myriad of physiological stages (i.e., environmental and genomic) results in solid advances toward a more comprehensive understanding of microbial community structure and function. Additionally, combinations of these model-driven hypotheses with omic studies will help guide the design and execution of synthetic phototrophic microbial communities.

## Methods

### Community-metabolic network reconstruction

CM-models were reconstructed using M-models of *S. elongatus* PCC7942, *i*JB792^32^, *B. subtilis*, *i*YO844^30^, *Y. lipolytica*, *i*Yali4^31^, *E. coli* W and K-12, *i*ECW1372^28^ and *i*ML1514^29^. Additional information about the models is shown in Table 1. All models were subjected to quality control and assessment tests (QC/QA) and integrated as CM-models using the COnstraint-Based Reconstruction and Analysis (COBRA) Toolbox^20^. The COBRA Toolbox is a MATLAB (MathWorks Inc., Natick, MA) software suite for quantitative prediction of cellular and multicellular biochemical networks, implementing the most comprehensive collection modelling methods and algorithms to perform high-throughput model-driven analysis^20^.

M-models were combined through the Shared Metabolite Pool (SMP) as described by Zuniga et al.^22^. This compartment streamlines the connectivity of individual models and the extracellular space based on each microorganism’s capabilities. Metabolites suitable for sharing were manually curated based on experimental data of community members in isolation. We performed a high-throughput growth assay (BIOLOG Inc, Hayward, CA) using *S. elongatus*, testing over 180 different carbon sources (see Supplementary Data 1). Metabolites added to the SMP for *E. coli*, *B. subtillis*, and *Y. lipolytica* were set in accordance with existing data in each M-model and existing information in the literature^29,31,57,58^. We adjusted the *i*Yali4 metabolic network to represent the genotype of *Y. lipolytica* Po1g, in particular the leucine auxotrophy was fulfilled by the exchange of isoleucine. Metabolic models were shared following the standard protocols for computational analysis^59^.

### Simulation tools and constraints

CM-models benchmarking was performed using the Gurobi Optimizer Version 5.6.3 solver (Gurobi Optimization Inc., Houston, Texas) in the COBRA Toolbox^20^ for MATLAB. We simulated the maximal growth rate of the community members using the Flux Balance Analysis (FBA)-associated algorithm OptCom^20^. This algorithm maximizes for the biomass objective functions or biomass reactions, which contain all metabolites that comprise organism biomass (e.g., carbohydrates, lipids, and proteins). The growth rate of the population results from the sum of fluxes through both biomass reactions of individual models. Constraints about CO_2_ and light consumption rates were imported from the *i*JB792 model. The sucrose secretion rate of *S. elongatus* was constrained to 0.182 mmol gDW^−1^ h^−1^. Benchmarking of experimental and predicted results was performed by calculating modeling statistics for the community members under mono- and coculture: true positive (TP), true negative (TN), false positive (FP), false negative (FN), and the confusion matrix and various measures of quality, such as accuracy, specificity, sensitivity, positive predicted, negative predicted, and Matthews correlation coefficient (TP + TN/(TP + TN + FP + FN))^60^. Metabolites productivity calculations were generated by constraining each metabolite production rate (0–2 mmol gDW^−1^ h^−1^) and maximizing for the biomass growth of both community members at the same time. We considered metabolite production feasible if both community members grew; these metabolites were selected and are shown in Fig. 2a. Phenotype phase planes were used to estimate the average growth and production rate while synthesizing butanal, ethanol, formaldehyde, and succinate. Growth and production rates under monoculture conditions for engineered strains were processed using the B10NUMB3R5 database^61^. Examples of the calculations are given in Supplementary Data 3.

### Robustness analysis and syntrophic pathways

FBA was used to identify metabolic exchange and to perform robustness analysis^20^. Exchange rates of these metabolites were iteratively varied to zero flux or double flux. Simulations deployed growth rate sensitivity of each member in the community. Core sets of vital metabolic exchanges were defined and compared for all CM-models (Supplementary Data 1). The solution space of M-models and CM-networks was scanned by uniformly sampling the solution space using optGpSampler^62^. All models were reduced for sampling. This unbiased assessment provides the possible flux distributions of the network at given constraints^63^. Sampling flux distributions were analyzed using the Symbolic Math Toolbox for MATLAB. The sampling results indicated the metabolic pathways/subsystems activity under monoculture and coculture conditions, and changes in pathway usage due to cocultivation were found.

We developed a method to compare expression data of individual microorganisms and communities with predicted flux distributions. Transcriptomic data for the pair *S. elongatus* and *E. coli* W was collected under monoculture and coculture conditions. Predicted flux distributions were normalized using the growth rate (µ) of individual microorganisms when using M-models and predicted growth rate of the community member when using CM-models, Eq. (1), where *i* denotes the index of the reaction and *j* the microorganism. The number of active reactions by subsystem is denoted by *n* and the total subsystems in the network by *k*, Eq. (2). For example *Sn*_*k,j*_ will contain the total normalized flux by subsystem (*k*) for the community member (*j*), Eq. (3). Supplementary Fig. 5 shows *Sn*_*k,j*_ obtained values for the SPC *S. elongatus* and *E. coli* K-12.

1 $${\mathrm{Flux}}_{i,x} = \frac{{{\mathrm{Flux}}_{i,j}}}{{\mu _j}}$$

2 $${\mathrm{Pathway}}\;{\mathrm{flux}}_{k,j} = \mathop {\sum }\limits_{i = 1}^{i = n} {\mathrm{Flux}}_{k,j}$$

3 $$Sn_{k,j} = \frac{{{\mathrm{Pathway}}\;{\mathrm{flux}}_{k,j}}}{{\left| {{\mathrm{Pathway}}\;{\mathrm{flux}}_{k,j}} \right|}}$$

We explicitly determined the term normalized subsystem (*Sn*_*k,j*_) for all subsystems, conditions, and community members. The culture condition (monoculture, coculture) exits at *m*. Predicted fluxes and gene expression data were normalized to values from −1 to 1 and compared using two sided *t*-test of The Statistics and Machine Learning Toolbox for MATLAB. The flux variability was determined using FVA^64^.

The difference in *Sn*_*k,j*_ from mono- to coculture conditions defines pathway usage as syntrophically active (i.e., higher flux), neutrally active (i.e., no change in flux), and deactivated (i.e., decrease in flux). Syntrophically active pathways were assumed to uptake or secrete mass from and to the partner as a result of metabolic exchange. Thus, *Sn*_*k,j,m*1_ represents each pathway and can take values from −1 to 1 depending on the total carried flux under mono- (*m1*) and coculture (*m2*) conditions, Eq. (4) and Eq. (5). The uptake of mass from the SMP to the network was given by:

4 $$Sn_{k,j,m1} \in \left\{ {0,1} \right\} \, > \, Sn_{k,j,m2} \vee Sn_{k,j,m1} \in \left\{ { - 1,0} \right\} \, > \, Sn_{k,j,m2}$$

And the secretion of metabolites to the SMP by:

5 $$Sn_{k,j,m1} \in \left\{ { - 1,0} \right\} \, > \, Sn_{k,j,m2} \vee Sn_{k,j,m1} \in \left\{ {0,1} \right\} \, > \, Sn_{k,j,m2}$$

The normalized values by subsystem defined the flux change or flux preservation in each pathway.

### Genetic stability depends on the phototrophic community participant

Genetic stability affects members’ prevalence and depends on the metabolic interactions between members. CM-models enabled quantification of the resistance to genetic damage in community members by studying metabolic network performance under genetic constraints. Single gene knockout (KO) simulations of over 12,800 genes in the metabolic networks (monoculture and community) were performed in turn, determining growth phenotypes under monoculture and community conditions. Genes predicted to be essential and non-essential were compared across conditions and growth patterns stabilizing, improving or decreasing the growth of the community members were determined.

### Strains and culturing conditions

*S. elongatus* PCC7942 (ATCC #33912) was engineered to secrete sucrose through the expression of the sucrose/proton symporter *cscB*^*+*^ ^33^. Inoculum culture of sucrose-secreting cyanobacterium was prepared using BG-11 (Sigma–Aldrich) medium supplemented with 1 g L^−1^ HEPES (pH 8.9) and 100 mM NaCl at 35 °C. BG-11 medium is composed of (in 1 L): 1.5 g NaNO_3_, 0.04 g K_2_HPO_4_, 0.075 g MgSO_4_·7H_2_O, 0.036 g CaCl_2_·2H_2_O, 0.006 g Citric Acid·H_2_O, 0.006 g Ferric Ammonium Citrate, 0.001 g Na_2_EDTA·2H_2_O, 0.02 g Na_2_CO_3_, 1 mL BG-11 trace metals solution. The BG-11 trace metals solution (in 1 L) consists of: 2.86 g H_3_BO_3_, 1.81 g MnCl_2_**·**4H_2_O, 0.22 g ZnSO_4_**·**7H_2_O, 0.39 g Na_2_MoO_4_**·**2H_2_O, 0.079 g CuSO_4_**·**5H_2_O, 49.4 mg Co(NO_3_)_2_**·**6H_2_O. Cultures were illuminated 24 h a day while shaking at 150 rounds per minute^7,8^. All experiments were performed with six to eight replicates. Flask cocultures were completed in 25 mL volumes in baffled flasks. Cultures were plated on rich media to check for contamination. Once cyanobacteria were exposed to coculture media (CoBBG-11 or CoYBG-11) no subsequent subcultures were made to avoid evolution away from sucrose secretion. To prepare precultures for *S. elongatus*, *S. elongatus* cultures at exponentially growing phase were centrifuged and washed twice with BG-11 and suspended in a proper volume of coculture medium. To prepare precultures for heterotrophic strains (*B. subtilis, E. coli*, and *Y. lipolytica*), single colonies were picked into LB-Miller media and grown until turbid at 37 °C. Cells were diluted into the appropriate coculture media containing 2% sucrose to acclimate the cells to coculture media, and maintained within log-phase growth (OD_600_ < 0.70) before use in cocultures. The log-phase cultures were centrifuged and washed twice with BG-11 and suspended in a proper volume of coculture medium. Coculture inoculum was prepared from cultures growing in exponential phase (OD_750_ 0.5) using ~2.5 × 10^7^ of *S. elongatus* (0.1 OD_750_) and ~7.5 × 10^6^ of heterotrophs (0.01 OD_600_). Experiments were carried out in batch cultures. Samples were obtained in exponential phase assuming pseudo steady state. The *Synechococcus elongatus* biomass concentration was analyzed by FACSCalibur flow cytometer (BD Biosciences, San Jose, CA) after adding BD Liquid Counting Beads (BD Biosciences, San Jose, CA). The absolute cell numbers in samples were determined by comparing cellular events to the counting beads events measured by the flow cytometer using the equation provided in the kit’s total dissolved solids document. For the heterotrophic strains, serial dilutions were plated on LB plates to determine cell numbers. Colony-forming units (CFU) were counted after overnight incubation at 37 °C. All heterotrophs were propagated in BG-11 (Sigma–Aldrich) using the same methods of Hays et al.^8^, while *E. coli* and *B. subtilis* 168 strains were maintained in LB-Miller at 37 °C and *Y. lipolytica* Po1g was maintained in YNB media at 25 °C as recommended by Yeastern Biotech Co. (Taipei, Taiwan). Strains were streaked from frozen stocks on LB plates before growth in liquid media. Seven *E. coli* knockout strains (*asp*C*, atp*C*, cyc*A*, cys*G*, ilv*C*, ilv*E*, phe*A*, pho*E*, tal*B*,* and *thr*C) were obtained from the Keio collection^65^ in Pamela Silver’s Lab at Harvard University. Knockout experiments in rich media were performed in 24-well plates using 0.8 mL of LB + 0.2 mL of BG-11 + 0.4% glucose. Inoculums were prepared as in growth experiments and we kept same culture conditions. Cell numbers were determined using LB agar plates. Samples were collected at 0, 8, 24, 32, and 48 h. The experiment was performed in triplicates.

### Analytical methods

Exchange capabilities of *S. elongatus* were determined experimentally by performing growth assays on 180 carbon sources. Biolog plates PM1 and PM2 were obtained from BIOLOG Inc. and the manufacturer’s protocol was followed^66^. Starting culture density was OD_750_ = 0.3, culture media was standard BG11, incubated 25–27 °C. Biolog plates were run in duplicate. The plates were incubated in a light room and examined at 490 nm for dye absorbance alterations and at 750 nm to assess optical density. Intracellular metabolites of *E. coli* and *S. elongatus* were extracted by the methanol-chloroform-water extraction method. This yielded 8.39 mg and 7.46 mg of dry metabolite extract, respectively. One milligram of each extract was used for NMR (Varian Direct Drive (VNMRS) 600 MHz spectrometer, Agilent Technologies, Santa Clara, CA, USA) and GC-MS (Agilent 6890 N, MSD 5975B, Santa Clara, CA, USA)^67^. Medium samples were dried and derivatized as well. 1D ^1^H NMR analysis was done with normal setting values for global metabolomics^68^. The remaining 7.39 mg and 6.46 mg were dissolved in 180 µL D_2_O with 0.5 mM NMR standard for NMR data acquisition.

RNA extraction, library generation and sequencing were performed by harvesting and snap-freezing cells in liquid nitrogen in biological triplicates for each condition. Cell lysates were prepared by grinding the frozen cell pellets in liquid nitrogen with 400 µl of RLT buffer (Qiagen RNeasy kit). RNA was stabilized by the addition of 2 ml Trizol reagent (Thermo Fisher Scientific) to each 1 ml of lysate. Total RNA was extracted using the RNeasy kit (Qiagen). mRNA was enriched using the Dynabeads mRNA purification kit (Invitrogen). Sequencing libraries were generated using the KAPA RNA HyperPrep kit (Roche) and following the recommended protocol. The libraries were paired-end sequenced on an Illumina HiSeq TM 4000, using 100 bp cycle kits. The sequencing adaptors were trimmed using the trim_galore program^69^. The reads were aligned to the *S. elongatus* genome in the NCBI database (Assembly No. GCF_000012525.1). Subread package-featureCounts (version 1.5.0-p1)^70^ was used to determine reads per each coding region. The aligned sequencing reads were used to determine RNA expression as reads per kilobase per million. The aligned reads were also used to determine differential gene expression using DESeq2^71^.

### Reporting summary

Further information on research design is available in the Nature Research Reporting Summary linked to this article.

## Supplementary information

Supplementary Information

Peer Review File

Reporting Summary

Description of Additional Supplementary Files

Supplementary Data 1

Supplementary Data 2

Supplementary Data 3

Supplementary Data 4

Supplementary Data 5

Supplementary Data 6

Supplementary Data 7

Supplementary Data 8

Supplementary Data 9

## Data Availability

Data supporting the findings of this work are available within the paper and its Supplementary Information files. A reporting summary for this Article is available as a Supplementary Information file. The datasets generated and analyzed during the current study are available from the corresponding author upon request. All sequencing reads were deposited in the Sequence Read Archive under BioProject PRJNA642094, with specific numbers listed in Supplementary Data 6. Additionally, all materials are available at https://github.com/cristalzucsd/SyntheticMicrobialCommunities. Source data are provided with this paper.

## Source data

Source Data

## Code availability

Code is available at GitHub [https://github.com/cristalzucsd/SyntheticMicrobialCommunities].
