# Supplementary material for: Synthetic microbial communities of heterotrophs and phototrophs facilitate sustainable growth
Source: Nat Commun. 2020 Jul 30;11:3803. doi: 10.1038/s41467-020-17612-8 (PMC7393147; doi:10.1038/s41467-020-17612-8)
Supplement: Supplementary file 1 — Supplementary Information [file 41467_2020_17612_MOESM1_ESM.pdf]

Synthetic microbial communities of heterotrophs and phototrophs facilitate sustainable microbial growth

Zuñiga *et al.*

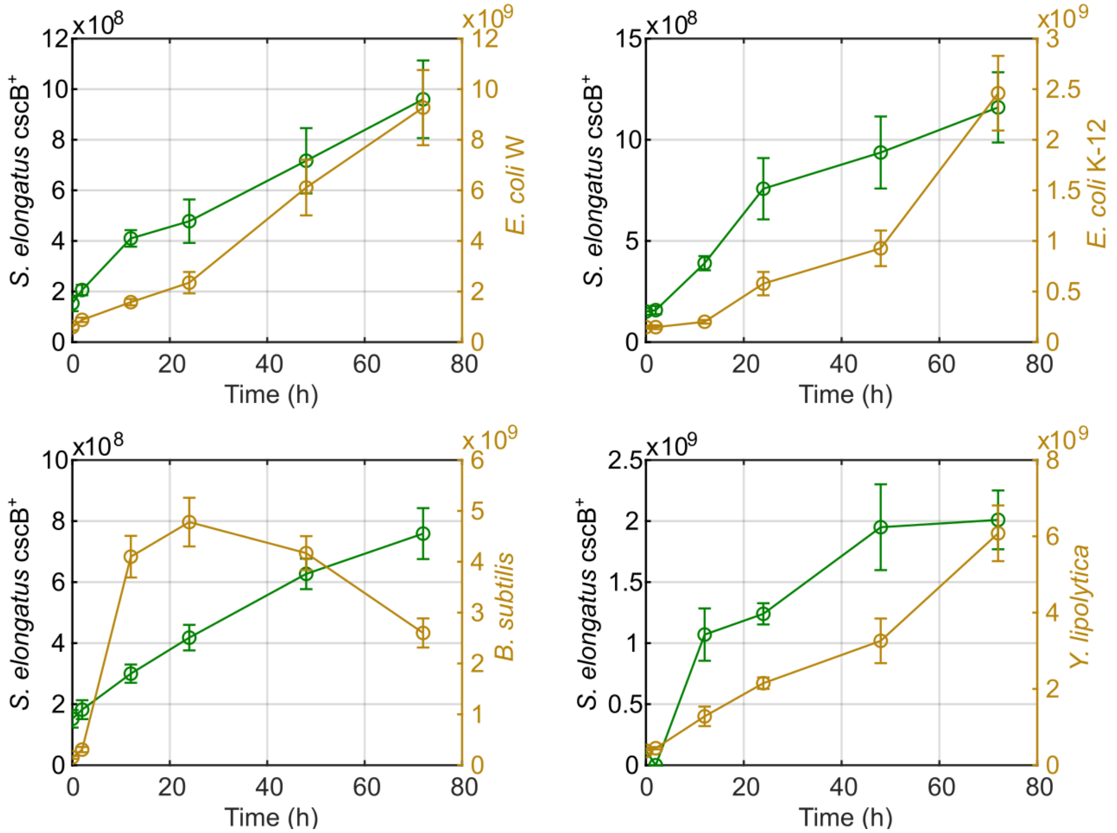

Supplementary Fig. 1. Experimental biomass measurements of the SPCs over the course of growth. All cultures were initiated and monitored for 72 h to ensure standardization in the determination of growth phenotypes for all SPCs. Individual microbe quantification was completed via CFU determination for heterotrophs and *S. elongatus* was quantified via flow cytometry. Error bars are the standard deviation of three biological replicates. Source data are provided as a Source Data file.

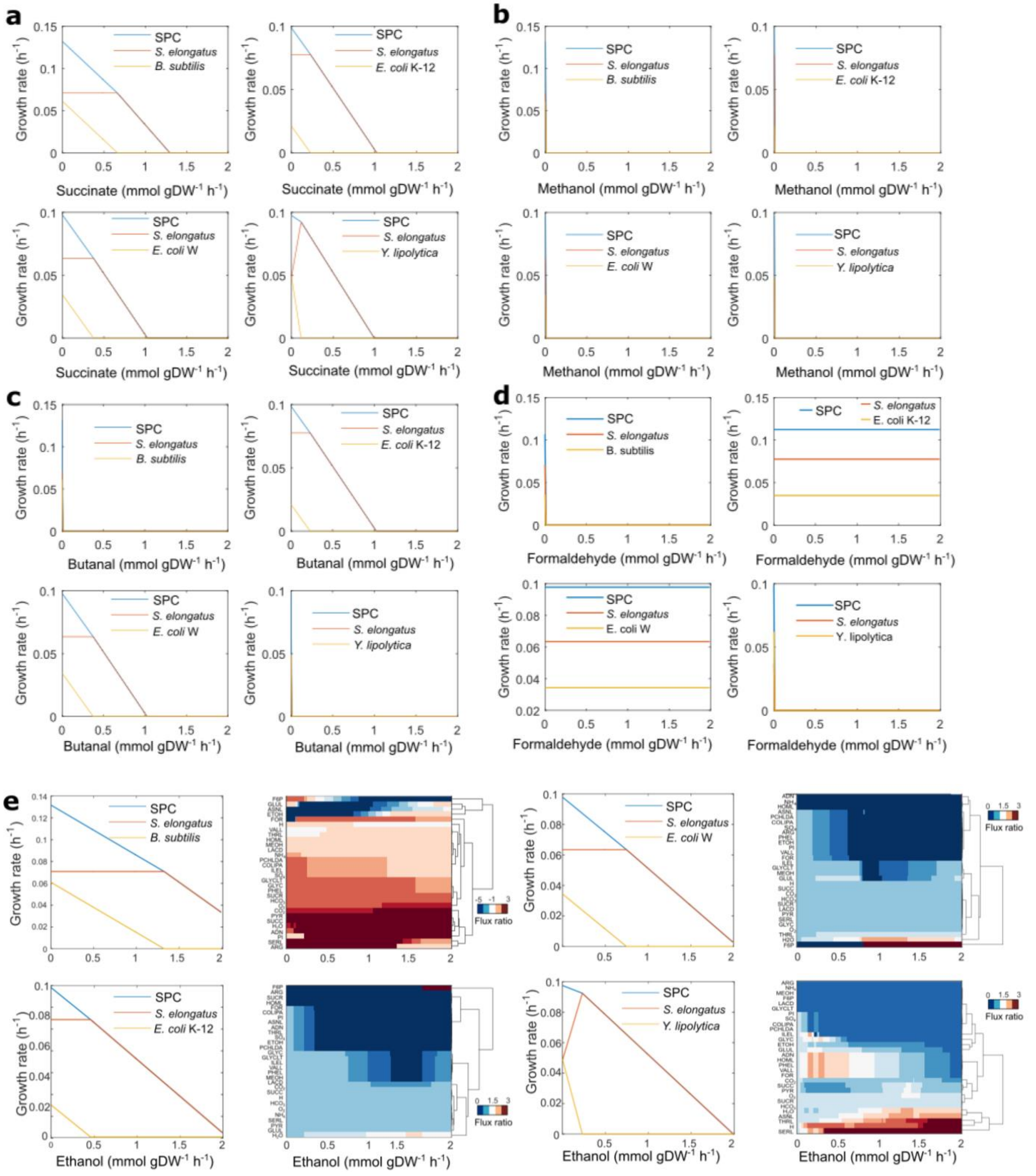

Supplementary Fig. 2. Synthesis of organic chemicals by the phototrophic communities. The CM-models were used to simulate the capabilities of SPC to produce different chemicals. **a** succinate, **b** methanol, **c** butanal (see Fig. 2), **d** formaldehyde and **e** ethanol. We predicted that all SPC can produce ethanol and succinate, however just the pairs containing *E. coli* strains were able to produce butanal and formaldehyde, on the other hand none of the SPC synthesized methanol. Yields estimated from average growth rates and metabolites production rates are shown in

Supplementary Table 3 for mono- and coculture conditions. Metabolites abbreviations are: arginine (ARG), ammonium (NH_4), methanol (MEOH), D-lactate (LACD), glycolate (GLYCLT), phosphate (PI), sulfate (SO_4), oligosaccharide lipid A (COLIPA), protochlorophyllide a (PCHLDA),isoleucine (ILEL), glycerol (GLYC), ethanol (ETOH), gluthamate (GLUL), adenosine (ADN), homoserine (HOML), phenylalanine (PHEL), valine (VALL), formate (FOR), succinate (SUCC), pyruvate (PYR), sucrose (SUCR), asparagine (ASNL), serine (SERL), and threonine (THRL).

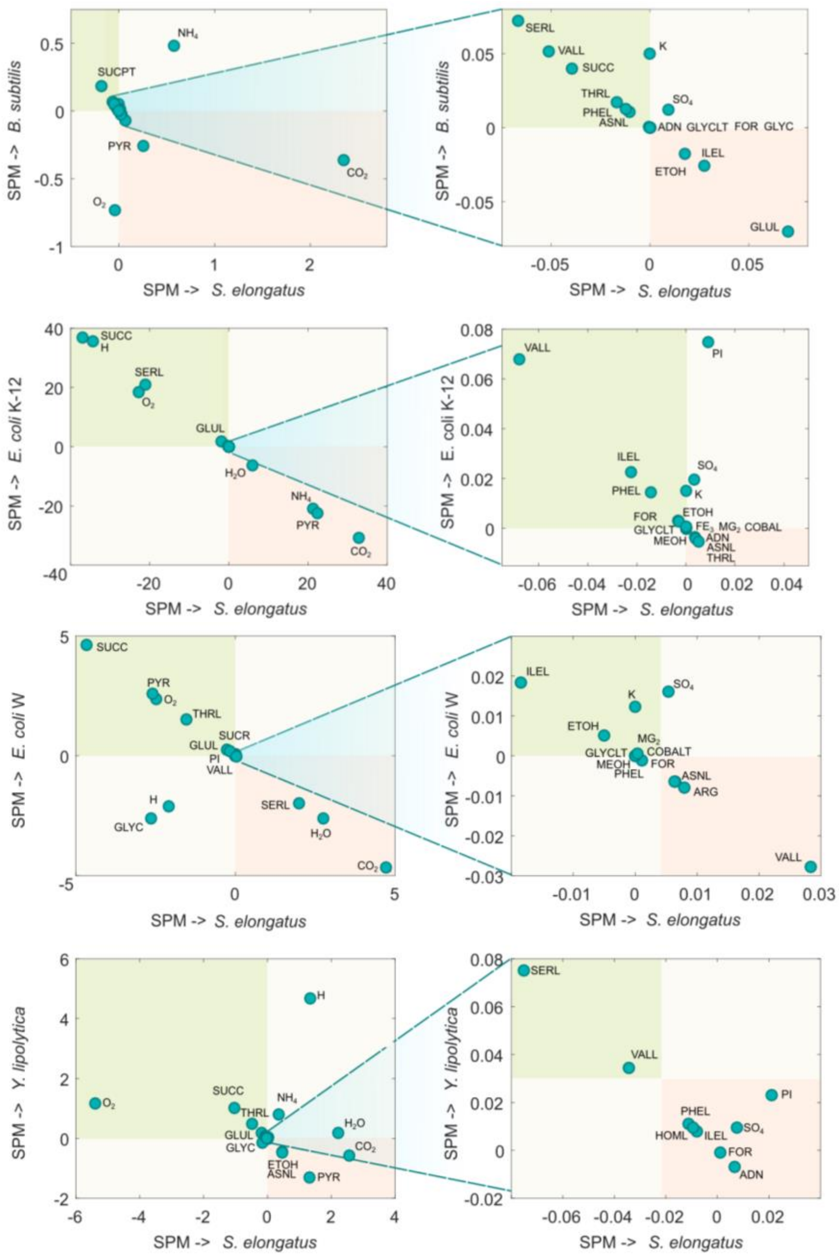

Supplementary Fig. 3. Predicted shared pool of metabolites for all phototrophic communities. The green panel encloses metabolites produced by the alga and consumed by the yeast (see Fig. 2). The red panel contains metabolites provided by the yeast to the alga. Metabolites in pearl-white panel are supplied (e.g. culture medium compounds) or secreted to the system.

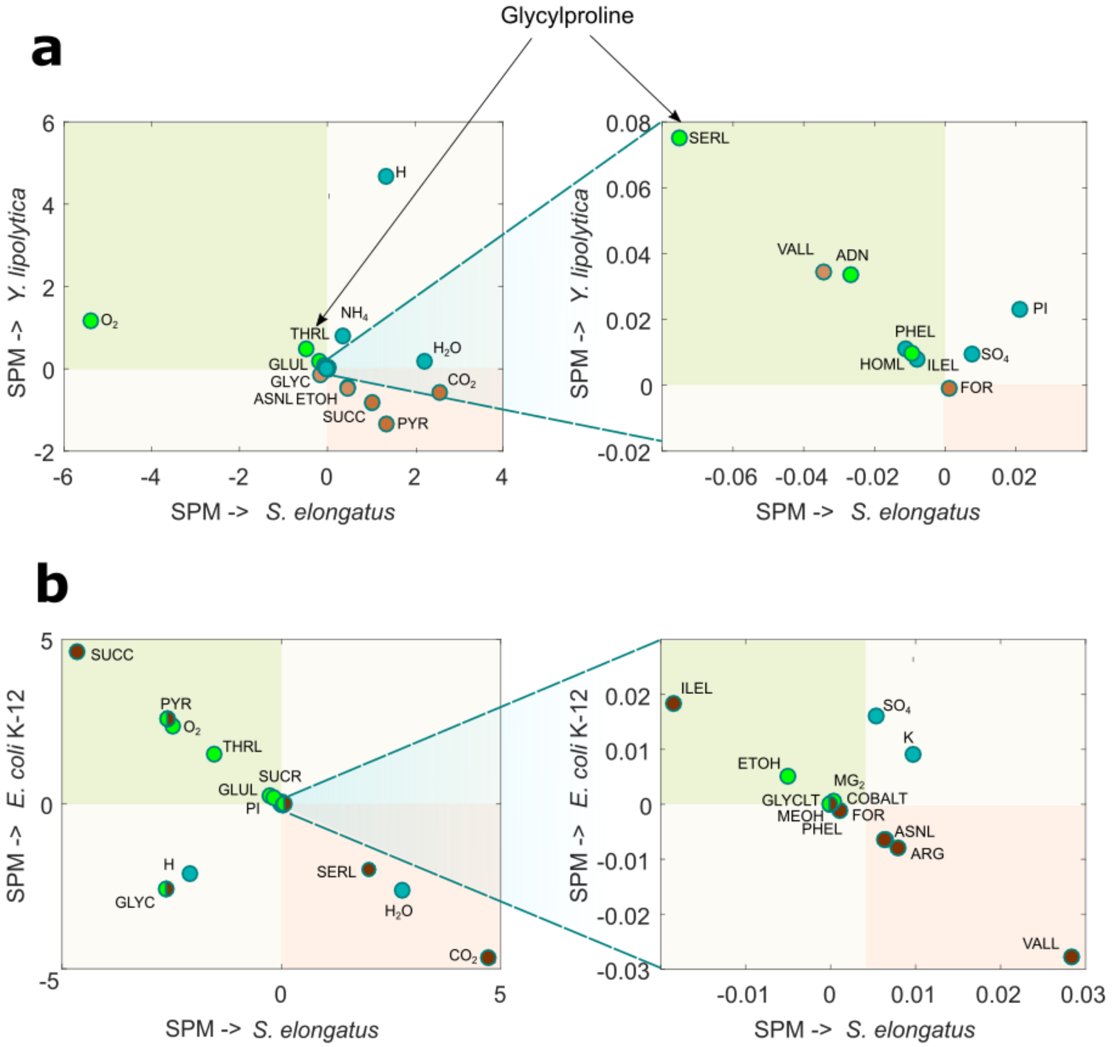

Supplementary Fig. 4. Metabolites identified through NMR and CG-MS analysis. **a** Predicted and validated metabolic exchange for the SPC *S. elongatus* and *Y. lipolytica*. **b** Predicted and validated metabolic exchange for the SPC *S. elongatus* and *E. coli* K-12. The green panel encloses metabolites produced by the alga and consumed by the yeast. The red panel contains metabolites provided by the yeast to the alga. Metabolites in pearl-white panel are supplied (e.g. culture medium compounds) or secreted to the system. Metabolites in brown were observed experimentally in the supernatants of the microbial communities.

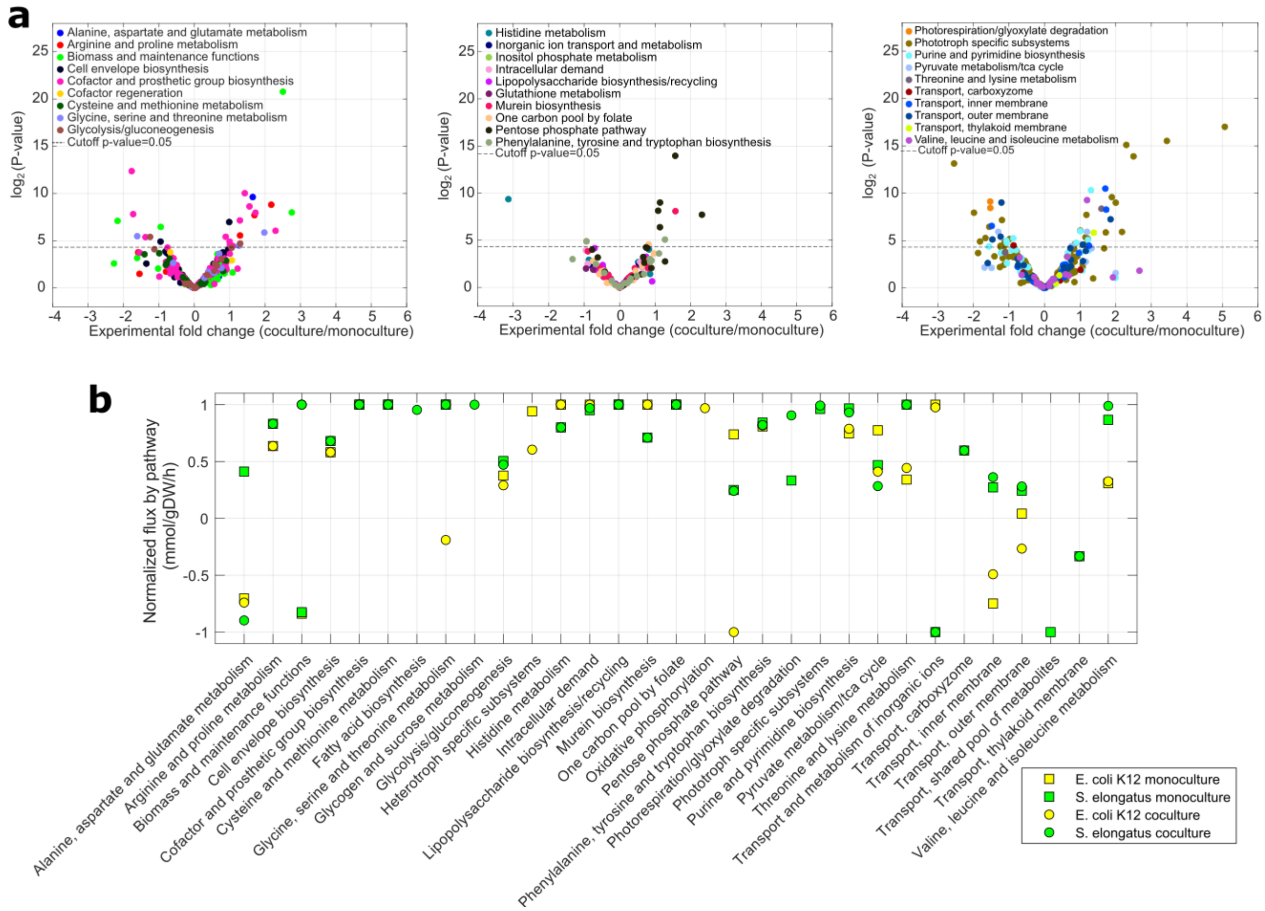

Supplementary Fig. 5. Differential expression analysis. a Significantly differentially expressed genes obtained from RNA-seq. **b** Predicted flux distributions under monoculture and coculture for the microbial community composed by *E. coli* K-12 and *S. elongatus*. Color markers of same color belong to the same community member, when they are located in a different position means that this pathway is perturbed during the coculture cultivation. Simulations were performed under the same environmental conditions and normalized values by pathway are shown in Figure 4b.

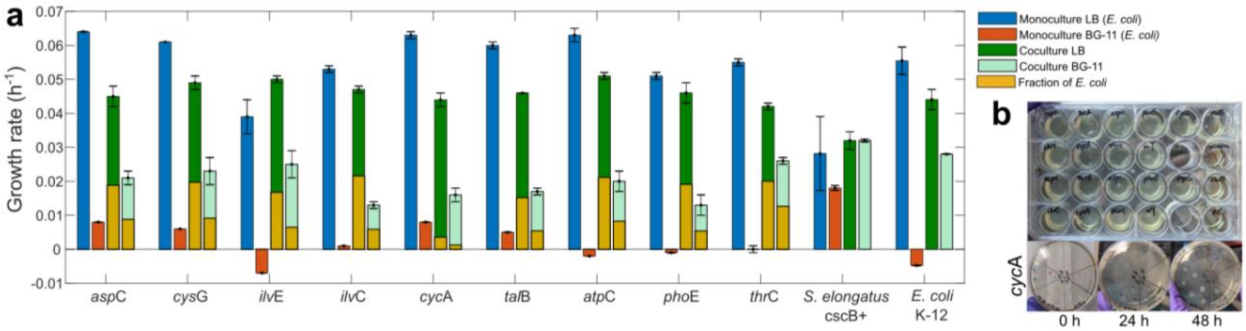

Supplementary Fig. 6. Community fitness in rich medium and minimal medium. a Five *E. coli* K-12 mutant strains with lethal phenotypes (*cys*G, *ilv*E, *ilv*C, *pho*E, *thr*C) and four with nonlethal phenotypes (*asp*C, *cyc*A, *tal*B, *atp*C) were experimentally tested under monoculture and coculture conditions while using the rich culture medium (80% Luria-Bertani, LB and 20% BG-11) and minimal medium BG-11. Experimentally determined growth phenotypes are shown with bars, error bars show the media and standard deviation of three replicates. **b** Knockout experiments were performed in 24-well culture plates. The number of cells of *E. coli* over the course of growth was determined using LB agar plates. Source data are provided as a Source Data file.
