# Supplementary material for: Synthetic microbial communities of heterotrophs and phototrophs facilitate sustainable growth
Source: Nat Commun. 2020 Jul 30;11:3803. doi: 10.1038/s41467-020-17612-8 (PMC7393147; doi:10.1038/s41467-020-17612-8)
Supplement: Supplementary file 4 — Description of Additional Supplementary Files [file 41467_2020_17612_MOESM4_ESM.pdf]

Description of Additional Supplementary Files

Supplementary Dataset 1
Metabolic capabilities of heterotrophs and phototrophs.

Supplementary Dataset 2
Constraints applied to the models

Supplementary Dataset 3
Metabolites production parameters by SPC

Supplementary Dataset 4
Unique metabolic capabilities in *E. coli* K-12 and *E. coli* W

Supplementary Dataset 5
Measurements of intracellular and extracellular metabolites by NMR and GC-MS

Supplementary Dataset 6
RNA-seq metadata

Supplementary Dataset 7
Expression analysis

Supplementary Dataset 8
Gene essentiality results

Supplementary Dataset 9
Genes with different role in monoculture and in the community
