# Supplementary material for: Synthetic microbial communities of heterotrophs and phototrophs facilitate sustainable growth
Source: Nat Commun. 2020 Jul 30;11:3803. doi: 10.1038/s41467-020-17612-8 (PMC7393147; doi:10.1038/s41467-020-17612-8)
Supplement: Supplementary file 2 — Peer Review File [file 41467_2020_17612_MOESM2_ESM.pdf]

Reviewers' comments:

Reviewer #1 (Remarks to the Author):

Overview:

In Zuniga et al., the authors report a comprehensive systems analysis of autotroph/heterotroph mixed communities. The basis of the formation of these communities is a previously-characterized strain of the model cyanobacterium, Synechococcus elongatus sp. PCC 7942 that was engineered to produce sucrose. In the context of the mixed communities, the engineered S. elongatus strain uses light and CO_2 to photosynthetically produce sucrose that can then be used as the primary carbon source for co-cultivated heterotrophic strains. 4 different species of heterotroph are evaluated in this manuscript, including 2 different strains of E. coli (K-12 and W), B. subtilis, and the fungus Yarrowia lipolytica. The authors develop metabolic models for these co-cultures by adapting published genome-scale metabolic models for each of these species and combining them by modeling a separate extracellular, shared pool of metabolites that can be exchanged across species. These models are evaluated against direct experimental co-cultures and the predicted vs. observed results are reported for growth rates, gene essentiality (in E. coli co-cultures), and metabolite exchange. Additionally, transcriptomic analysis is conducted for the co-cultures in order to provide an indication of how co-mingling of unrelated species influences the metabolism of the cyanobacterium.
Altogether, these experiments represent a more comprehensive analysis of autotroph/heterotroph co-cultures that expands upon prior reports that have utilized the sucrose-secreting cyanobacteria (S. elongatus cscB+) to construct synthetic co-cultures. Therefore, the report has the potential to significantly advance in our understanding of the fundamental and emergent interactions in such ‘bottom-up’ engineered consortia.
Beyond the direct relevance to the field of engineered cynobacteria consortia for bioproduction, there may be implications of this work more broadly to other engineered co-cultures/consortia as well as to the organization of natural microbial consortia. The current formatting of the manuscript leaves a number of open questions. Especially given the complexity of the systems being analyzed, the multiple co-cultures evaluated, and the lack of specific outcomes that can be succinctly summarized, it is possible that the impact of this study may be more effectively communicated in a longer-format.

Major comments/questions:

In this reviewer’s opinion, this work is a significant extension upon prior art in the domain of engineered cyanobacteria-heterotroph communities and, as such, could be suitable for publication in a high impact journal. Of particular note, the ability of the developed co-culture metabolic models to predict some gene essentiality outcomes seems to be a strength of the report. Similarly, the predictions of emergent metabolic fluxes, and those that may be the most important to maximize when considering co-culture bioproduction (Figure 2CD), were very interesting and could advance the sophistication of co-culture metabolic engineering, if implemented. Finally, the direct measurements of a number of extracellular metabolites in co-cultures by NMR was viewed as an important measurement with implications for understanding emergent cross-species interactions. However, the short formatting of this article may not be the most conducive to conveying the significance of the results. As it stands, it is somewhat difficult to digest/understand the significance of a number of the results. It is possible that many of the questions below may be addressed by reformatting and/or expanding upon the description and discussion of the results.
First, this reviewer was unfortunately unable to access the Supplementary Tables (1-12) that are referenced in this manuscript. There seems to be some substantial information that is reported in these Tables, especially Table 1 and 2. But I was not able to find a link on the Nature Communications reviewer portal or a through the cited GitHub URL. Some of this information seemed to be central to the evaluation of the manuscript.

It was curious that the metabolic models predicted a reduction in growth rate for S. elongatus when

grown in almost all co-cultures relative to the monoculture (Figure 1B). This prediction seems to agree with the author's experimental results, but goes against the cyanobacterial growth enhancement observed in previously published co-cultures containing the same species (Hays 2017). Given that there is no competition for the primary carbon source between autotroph (CO2/bicarbonate) and heterotroph (sucrose and other cyanobacterial photosynthates) and that carbon is likely to be the rate-limiting nutrient for growth under the conditions tested; is there a simple description of why the models predict this result? Light shading? Competition for a micro-nutrient in the media? Perhaps the models suggest that cyanobacterial exudates are taken back up in cyanobacterial monocultures in a way that promotes growth? It is possible that if there were a common theme underlying this predicted growth decrease, it might be possible to improve co-culture bioproduction by correcting for this predicted incompatibility.

There was potentially exciting predictions of metabolic exchanges that might be particularly important to promote robust growth in the synthetic co-cultures (e.g., see line 249: "Our computational studies suggest optimized growth should include the exchange of a number of additional metabolites). However, the important exchanges that were predicted were not explicitly reported. Furthermore, the logical connection between the predicted metabolites that should be exchanged to promote robustness, and the measurements of intracellular metabolites by NMR was unclear. For example, is it not obvious that amino acids will accumulate (and to different levels) within both microbial species? Some of my confusion with understanding this section may be a result of not having access to Supplemental Tables (esp Supplemental Table 1).

The section on emergent metabolite exchanges and gene expression (paragraph beginning on line 265) was generally difficult to follow. Yet, this section could also be one of the most significant with regard to the insights provided by the metabolic modeling. This section seems to report the prediction of significant emergent metabolic exchanges that are not specifically programmed in the "toy model". A lack of access to some of the Supplemental Tables (including Table S6) precluded more careful evaluation of the predicted flux changes with the observed gene expression data, and may have generally obscured comprehension. Despite significant interest in the results reported and time invested, this reviewer was unable to understand what was being shown.

What are the primary fluxes that the model predicts to support E. coli K-12 growth in the presence of S. elongatus CscB+ (given that K-12 is unable to utilize sucrose)? Is it a surprise, given the relatively high rate of efflux of sucrose in comparison to other carbon compounds, that the growth of K-12 is similar to that of E. coli W (which can utilize sucrose)?

Of somewhat lesser importance, the context of this work was not framed in reference to the multiple studies that precede it.
There are now multiple published manuscripts describing the use of this same cyanobacterial strain (S. elongatus CscB+) for the formation of co-cultures. Indeed, 3 of the 4 co-cultures (E. coli W, E. coli K-12, B. subtilis) with this strain have been previously evaluated, and a couple relevant publications of such co-cultures are not cited in this manuscript (Smith, MJ et al., ACS synthetic biology (2016); Smith, MJ et al. Biotechnology and bioengineering (2017); Weiss, TL et al., 2017 Metabolic Engineering). Without a priori knowledge of these works, it may be difficult for the reader to contextualize some of the significance of what is reported herein.

Minor Points:
The significance of Figure 1A is not clear from the current format. Additionally, there are no bootstrap values shown at nodes on the current panel, as is reported in the figure legend. Based on the current formatting, this panel would be better assigned to a supplement in order to make room for more impactful information.

In Figure 2B, it is unclear what the divided colored bars represent. Is the green vs. the other color of the bar graph meant to represent the proportion of the compound of interest (e.g., formaldehyde)

that is contributed by each of the partner species? Or merely meant to represent the abundance of each species? If the latter, is this proportion based on cell number, dry biomass, cell volume, or other?

It is somewhat surprising that the predicted feasible metabolites for co-cultures of K-12 is much higher than the predicted co-cultures of E. coli W, particularly as the primary difference between these strains is a relatively small number of genes related to sucrose catabolism. Is there insight to be gained about this based on the models, or is this likely an artifact of differences in the complexity of the relative genome scale E. coli models (i.e., iECW1372 vs iJO1366W)?

It is unclear why the metabolic models of the co-culture would predict a failure of S. elongatus to grow in certain pairings with E. coli knockouts – given that the cyanobacterium is not dependent upon heterotroph viability. For example, as reported in Figure 3B, the model appears to predict no growth of any species when E. coli mutants in the genes phoE and talB are used. The authors statement of line 206 "Interestingly, genetic damage in the heterotrophic partner did not significant affect the growth rate of the phototroph…" is somewhat confusing in this context. Why/when would "genetic damage" in the heterotrophic partner ever be expected to have this result? Are there insights to be gained from these false negatives or improvements that could be incorporated into the models?

A few typos in Supplemental Figure 1 in the figure legend – most noticeably, the statement that "none of the SPC synthesized methanol and formaldehyde" disagrees with the panel and the results shown for formaldehyde production in Figure 2B.
Generally, a number of other (often small) typographical errors in the manuscript could obscure reader comprehension of the results.

Reviewer #2 (Remarks to the Author):

In the presented manuscript Zuniga et al. describe several microbial co-cultures between heterotrophs and phototrophs. They mainly used genome-scale metabolic models to evaluate the potential interactions between the species in co-culture. Despite the interest on the topic and the novelty of the modelling methodology, there are some flaws in the way the article is described that would need to be amended before publications.

Abstract, introduction:
-Several aspects on the text, including the title and abstract are a misleading. There is a constant reference to bioproduction, but there is no bioproduction at all in the results (just a brief quantification of metabolites in the media). The real strength of the manuscript is the analysis of co-cultures using modelling and the experimental validation of that approach. By reading the title and abstract the reader expect a completely different content.
-The reader expect that all the conclusion apply to the different microbial systems studied but sadly, the validations have been only carried out in E. coli co-culture.

First part of results: This is the most important part of the paper, where the new modelling aspects are shown and validated with growth. This must be expanded, and negative results must be explained with more detail. Some comments below:
-it is a bit unclear how the parameters were calculated to correlate models and experiments, was everything done in chemostat to represent the steady state of the model? Or flask?
-L85. The strains of bacillus and yarrowia must be mentioned too.
-L124. Why this disagreement with the model? What is the predicted metabolite exchange happening here?
-L129. The authors should explain why K12 is growing, what are they exchanging? What is the prediction of the model?

- what is the volume of the pre-cultures used to inculate? How this affect growth? Are pre-cultures properly washed?
- Fig 1b should also show how is the growth in g/L as it is not the same to have a very quick growth but with final biomass produced, what is especially relevant in bioproduction. The full growth curves should be shown in supplementary. How is the quantification of each cell type?

Second part of results: Here the authors are measuring the secretion of different metabolites to the media. This section is not very clear but is certainly not related to bioproduction. Firstly, the well known metabolites produced by the selected organisms such as acetone, acetoin or citric acid are not considered and only others are measured that are not really produced in relevant amounts by these organisms. Then the values of production are low, and thus, more in the range of things that are secreted or accumulated rather than things with potential for bioproduction, as it is understood in microbial biotechnology. This section is mainly talking about E. coli K12.
-the 'production' is shown as mmol/gDCW/h (Fig. 2b) which are the units given by the model but not units often used in bioproduction experiments. How can you translate this to titers and yields? Is any of the metabolites actually produced in any significant amount for bioproduction? They all look very low to me.
-the metabolite exchange predicted by the model in the different scenarios are not validated, are they?
-The text in this section is almost exclusively about E. coli co-cultures.

Third part of results:
-only 3/4 KO were validated experimentally, maybe more KO should be tested. Why the authors selected only 10?
-The validation has been done only in E. coli. If the authors want to claim the validation as general, other organisms should be tested too.
-Figure 3 B, why some of the co-cultures did not allow grow of elongatus in the model?

Four part of results:
-L239. How can Fructose 6P be predicted as exchangeable when it cannot cross membranes?
-L241. Confirmed again only for E. coli.
-Make a clear table with the prediction of metabolites exchanged and the validated ones by NMR or GC. Try to explain the mismatches.
-L261. Are the intracellular metabolites of any of the species different in co-culture than in monoculture?
-RNAseq validation only for E. coli.
-Table 2 NMR. Why coli is producing sucrose? More than elongatus?
-Table 3 should show the extracellular metabolites too, which are more relevant for this work.

Methods:
Media composition for co-cultures is missing. Description of BG-11 could be also a good addition.

General comments and suggestion:
I think the manuscript has potential, but it is misleading in many aspects. To me, most of the validations has been done with E. coli as co-culture. I think the manuscript will benefit from a clearer story if the paper is focused in this co-culture only. Maybe a short paragraph can be added at the end to say that this can be applied to any other organisms with model available and you can add in supplementary some of the modelling results you have for other microbes. On the contrary, as it is written now the authors would need to include more experimental validations for all the different microbes.
I would also focus in the novelty of this article which is the modelling and its validation. I would try to explain more how it works and how people could use it. The authors must avoid highlighting too much the bioproduction, as this work is not about bioproduction at all. The potential of this work for

bioproduction can be discussed in the discussion section.

Reviewer #3 (Remarks to the Author):

In this manuscript, the authors conduct a combined theoretical and experimental study exploring the interactions among five species: Synechococcus elongatus, two strains of Escherichia coli, Yarrowia lipolytica, and Bacillus subtilis. Flux within different defined communities are predicted using community metabolic models, and then model predictions are validated with experimental expression, KO, and metabolomics data.

Community model analyses have been conducted previously within a wide range of systems to date, including studies that compare community model flux predictions with expression data. That said, this is the first study to examine these specific species and combine an autotroph with a range of defined heterotrophs. This is perhaps the first study to confirm that gene essentiality profiles are altered when species grow in community, although TN-seq data to this effect has been previously published. It would be interesting to compare these results with changes in gene essentiality when growing in rich media vs minimal media. One would expect changes to be similar.

The metabolomics data is somewhat new as a validation of interactions in community models, although the data as currently presented seems unclear and inconclusive. The authors' logic in explaining what changes are occurring and why needs to be clarified. Overall, the work is an interesting demonstration and validation of community metabolic models, but beyond this it's unclear what new insights have been revealed by the work. A deeper message may be here, but unfortunately, the paper has many grammatical and organizational issues that need to be resolved before it can be fully understood by readers.

My current significant concerns include:
1.) On line 105 the authors say: "The modeling compartment Shared Metabolite Pool (SMP) was manually curated in all CM-models, including experimental and genetic evidence of metabolic exchange capabilities by each community member."

How different were the metabolite exchange constraints in the community models from the transport reactions built into the original isolate species models? Were more exchanges added based on experimental evidence? Were some removed?

2.) Can the authors explain why the community models predict increased growth in S. elongatus when cocultured with Y. lipolytica?

3.) How were CM-model growth rates predicted? What was the objective function? The authors mention using the COBRA toolbox community simulation tools in the methods, but then explain very little about how the tools work. This relies on the reader to have more expertise in COBRA community models than will be typical, so it is recommended that the methods describe how the community models work in a little more detail.

4.) The expression flux comparison method could use much better description. For example, is the individual cell or community model growth used to normalize fluxes in equation 1? The notation in these equations is generally not well described. For another example, it's not at all clear what this sentence is trying to say: "Snk,j deploys pathways usage as syntrophically active, neutrally active, and deactivated."

5.) On line 138, the authors state: "Metabolite production feasibility simulations were combined with biomass growth maximization of both community members. When the metabolite production rate and

growth of both community members were higher than zero, the tested metabolite was counted as feasible (Fig. 2a)."

This implies that maximum growth was computed first, then the authors attempted to produce the metabolites? So if a metabolite decreased the growth yield, it's production would be reported as zero? Is this correct? Also, when you say the biomass growth of both members was maximized, did you maximize the sum of the biomass or both species? Or maximize one species first, then maximize the other? Order would matter in that case. Also, how did community production profiles compare with individual species production profiles for each species?

6.) How do community knockout results compare with individual species growing in very rich media?

7.) Could the thrC results have to do with regulation of threonine transporters in the donor or receiver strains? If this mutant is simply grown in a very rich media, is it viable?

8.) The higher levels of certain metabolites does not necessarily indicate the presence of metabolic exchange within the co-cultures. Can the authors explain the logic of this analysis further? Were the metabolite level increases statistically significant? How much higher were the levels in co-culture? This isn't totally clear when only Z-scores are provided.

Minor comments:
The manuscript includes numerous grammatical errors and sentences that are difficult to understand in their present state. As such, it could use a very thorough edit for understandability and grammar.

Response to the reviewers NCOMMS-19-28644

Reviewer #1 (Remarks to the Author):

Overview:

In Zuniga et al., the authors report a comprehensive systems analysis of autotroph/heterotroph mixed communities. The basis of the formation of these communities is a previously-characterized strain of the model cyanobacterium, *Synechococcus elongatus* sp. PCC 7942 that was engineered to produce sucrose. In the context of the mixed communities, the engineered *S. elongatus* strain uses light and CO_2 to photosynthetically produce sucrose that can then be used as the primary carbon source for co-cultivated heterotrophic strains. 4 different species of heterotroph are evaluated in this manuscript, including 2 different strains of *E. coli* (K-12 and W), *B. subtilis*, and the fungus *Yarrowia lipolytica*. The authors develop metabolic models for these co-cultures by adapting published genome-scale metabolic models for each of these species and combining them by modeling a separate extracellular, shared pool of metabolites that can be exchanged across species. These models are evaluated against direct experimental co-cultures and the predicted vs. observed results are reported for growth rates, gene essentiality (in *E. coli* co-cultures), and metabolite exchange. Additionally, transcriptomic analysis is conducted for the co-cultures in order to provide an indication of how co-mingling of unrelated species influences the metabolism of the cyanobacterium. Altogether, these experiments represent a more comprehensive analysis of autotroph/heterotroph co-cultures that expands upon prior reports that have utilized the sucrose-secreting cyanobacteria (*S. elongatus* cscB+) to construct synthetic co-cultures. Therefore, the report has the potential to significantly advance in our understanding of the fundamental and emergent interactions in such 'bottom-up' engineered consortia. Beyond the direct relevance to the field of engineered cyanobacteria consortia for bioproduction, there may be implications of this work more broadly to other engineered co-cultures/consortia as well as to the organization of natural microbial consortia. The current formatting of the manuscript leaves a number of open questions. Especially given the complexity of the systems being analyzed, the multiple co-cultures evaluated, and the lack of specific outcomes that can be succinctly summarized, it is possible that the impact of this study may be more effectively communicated in a longer-format.

Major comments/questions:

In this reviewer's opinion, this work is a significant extension upon prior art in the domain of engineered cyanobacteria-heterotroph communities and, as such, could be suitable for publication in a high impact journal. Of particular note, the ability of the developed co-culture metabolic models to predict some gene essentiality outcomes seems to be a strength of the report. Similarly, the predictions of emergent metabolic fluxes, and those that may be the most important to maximize when considering co-culture bioproduction (Figure 2CD), were very interesting and could advance the sophistication of co-culture metabolic engineering, if implemented. Finally, the direct measurements of a number of extracellular metabolites in co-cultures by NMR was viewed as an important measurement with implications for understanding emergent cross-species interactions. However, the short formatting of this article may not be the most conducive to conveying the significance of the results. As it stands, it is somewhat difficult to digest/understand the significance of a number of the results. It is possible that many of the

questions below may be addressed by reformatting and/or expanding upon the description and discussion of the results.

We thank the reviewer for her/his time and effort to review our manuscript as well as for her/his valuable comments. We have now expanded several sections of the manuscript and provided a more detailed description of all our findings. We have also edited several sections of the manuscript, for example expanded Fig. 2A deploying metabolic exchange in a more comprehensive fashion. Additionally, we included several new Supplementary Materials, such as a new Supplementary Fig. 1 and Supplementary Table 3 and 5.

First, this reviewer was unfortunately unable to access the Supplementary Tables (1-12) that are referenced in this manuscript. There seems to be some substantial information that is reported in these Tables, especially Table 1 and 2. But I was not able to find a link on the Nature Communications reviewer portal or a through the cited GitHub URL. Some of this information seemed to be central to the evaluation of the manuscript.

We thank the reviewer for her/his valuable comment. We apologize for not including the Supplementary Tables in the compressed file provided during the submission of the manuscript. Table 1 and 2 were included in the main text and Supplementary Tables 1-9 are now included in this version of the manuscript. All supporting materials are now also available at https://github.com/cristalzucsd/SyntheticMicrobialCommunities.

It was curious that the metabolic models predicted a reduction in growth rate for *S. elongatus* when grown in almost all co-cultures relative to the monoculture (Figure 1B). This prediction seems to agree with the author's experimental results, but goes against the cyanobacterial growth enhancement observed in previously published co-cultures containing the same species (Hays 2017). Given that there is no competition for the primary carbon source between autotroph (CO2/bicarbonate) and heterotroph (sucrose and other cyanobacterial photosynthates) and that carbon is likely to be the rate-limiting nutrient for growth under the conditions tested; is there a simple description of why the models predict this result? Light shading? Competition for a micro-nutrient in the media? Perhaps the models suggest that cyanobacterial exudates are taken back up in cyanobacterial monocultures in a way that promotes growth? It is possible that if there were a common theme underlying this predicted growth decrease, it might be possible to improve co-culture bioproduction by correcting for this predicted incompatibility.

We thank the reviewer for pointing this out. We updated the materials and methods section, clarifying the inoculum ratios used during the experiments shown in Fig. 1b. We followed the cultivation instructions from Hays et al. using an optimized inoculum ratio of 3:1 (cyanobacteriun:heterotroph). This optimized inoculum ratio enabled the growth of heterotrophs better than photoautotrophs in batch cultivation, triggering the cultivation of stable cocultures that prevented the heterotroph from outcompeting *S. elongatus*. Community stability not only relies on inoculation ratios, but also on appropriate cultivation conditions (e.g. media composition, solid or liquid media, batch or continuous cultivation) as well as on light:dark illumination cycles as demonstrated by Hays et al. Hays et al. also showed that changes in those conditions could lead to different growth phenotypes of the community members. For example, when they used inoculum ratios that varied up to two orders of magnitude, experimental outcomes benefited the community members. Since our experiments were performed in batch cultures we cannot discard possible nutrient limitations, especially limitations associated with metals or other micronutrients. Our simulations showed the importance of these metabolites for the communities to thrive. We have now highlighted the importance of these parameters in the manuscript, describing specifics of our cultivation conditions.

Furthermore, we have now included all details about our experimental conditions in the manuscript, such as:
"*S. elongatus* was propagated in BG-11 (Sigma-Aldrich) plus 1 g/L HEPES, pH 8 in constant light at 35 °C. Cyanobacteria cultures were checked for contamination via plating on rich media. *B. subtilis* and *E. coli* were propagated in LB-Miller (EMD Millipore). Co-culture medium was optimized consisting of BG-11 supplemented with 106 mM NaCl, 4 mM NH4Cl and 25 mM HEPPSO, pH 8.3-KOH.

To prepare pre-cultures for *S. elongatus*, *S. elongatus* cultures at exponentially growing phase were centrifuged and washed twice with BG-11 and suspended in a proper volume of co-culture medium. To prepare pre-cultures for heterotrophic strains (*B. subtilis, E. coli,* and *Y. lipolytica*), single colonies were picked into LB-Miller media and grown until turbid at 37 °C. Cells were diluted into the appropriate co-culture media containing 2% sucrose to acclimate the cells to co-culture media, and maintained within log phase growth (OD600 < 0.70) before use in co-cultures. The log phase cultures were centrifuged and washed twice with BG-11 and suspended in a proper volume of co-culture medium. The inoculum volume for *S. elongatus* and the heterotrophic strains were 1 mL and 200 µL, respectively, to achieve initial cell numbers of $\sim 2.5*10^7$ for *S. elongatus* (OD750=0.1) and $\sim 7.5*10^6$ for the heterotrophs (OD600=0.01).
Heterotrophic growth was measured by inoculating washed cells at 0.01 OD600 (bacteria) or 0.05 OD600 (yeast) into fresh co-culture media containing sucrose. Data for growth rate measurements were collected from 25 mL flask cultures while 96-well plates with 1 mL culture volumes were used to assay growth in a gradient of sucrose concentrations (0.156 mg/mL to 10 mg/mL, Fig. 2c) as well as growth in conditioned media. OD600 of plates was read on a Synergy Neo plate reader (BioTek, Winooski VT)."

There was potentially exciting predictions of metabolic exchanges that might be particularly important to promote robust growth in the synthetic co-cultures (e.g., see line 249: "Our computational studies suggest optimized growth should include the exchange of a number of additional metabolites). However, the important exchanges that were predicted were not explicitly reported. Furthermore, the logical connection between the predicted metabolites that should be exchanged to promote robustness, and the measurements of intracellular metabolites by NMR was unclear. For example, is it not obvious that amino acids will accumulate (and to different levels) within both microbial species? Some of my confusion with understanding this section may be a result of not having access to Supplemental Tables (esp Supplemental Table 1).

We thank the reviewer for this constructive comment. We have decided to modify Figure 3a including heatmaps of predicted fluxes of exchanged metabolites by all cocultures. We also added the percentage of the total flux exchange attributed to each metabolite. The updated figure highlights metabolites that were successfully identified through targeted metabolomics of the culture supernatants. The new version of the manuscript also has a new Supplementary Table 5 that shows experimental measurements of intracellular and extracellular metabolites under both mono- and coculture conditions.

The section on emergent metabolite exchanges and gene expression (paragraph beginning on line 265) was generally difficult to follow. Yet, this section could also be one of the most significant with regard to the insights provided by the metabolic modeling. This section seems to report the prediction of significant emergent metabolic exchanges that are not specifically programmed in the "toy model". A lack of access to some of the Supplemental Tables (including Table S6) precluded more careful evaluation of the predicted flux changes with the observed gene expression data, and may have generally obscured comprehension. Despite significant interest in the results reported and time invested, this reviewer was unable to understand what was being shown.

We apologize for the missing information and the lack of clarity. We noticed that this section was very brief (due to space limitations) and thus difficult to understand due to the lack of supporting information in the main Figures. We have now expanded this section, emphasizing the development of computational methods as well as how predicted outcomes can be compared with experimentally determined expression profiles. We also updated Figure 3c showing only experimental data. Data in the new Fig. 3c is based on raw data information shown in Table S7.

What are the primary fluxes that the model predicts to support *E. coli* K-12 growth in the presence of *S. elongatus* CscB+ (given that K-12 is unable to utilize sucrose)? Is it a surprise, given the relatively high rate of efflux of sucrose in comparison to other carbon compounds, that the growth of K-12 is similar to that of *E. coli* W (which can utilize sucrose)?

We thank the reviewer for pointing this out – this was actually a surprising finding for us as well. The revised version of the manuscript describes how *E. coli* K-12 is sustained by *S. elongatus* through the exchange of key metabolites (e.g. succinate, pyruvate, and serine). CM-models enable users to predict the flux of metabolites necessary for successful growth of both community members. These calculations are based on flux balance analysis (FBA), which is a widely used approach for studying biochemical networks, in particular the genome-scale metabolic network reconstructions of individual organisms. Here, we applied FBA to our community networks, estimating the flow of metabolites through the connected networks, thereby making it possible to predict the growth rate of each organism. Fluxes (mmol/gDW/h) through exchange reactions showed that the synthetic phototrophic community (SPC) containing *E. coli* K-12 was able to thrive by exchanging succinate, serine, glutamine and pyruvate (average fluxes 1-40 mmol/gDW/h). Additionally, fluxes two orders of magnitude smaller included valine, isoleucine, phenylalanine, formate, ethanol, asparagine, and threonine (Supplementary Fig. 2). NMR and GC-MS analysis of the coculture supernatants confirmed the presence of succinate, pyruvate, serine, isoleucine, formate, asparagine, arginine and valine (Supplementary Fig. 3). For the SPC containing *E. coli* W metabolites such as sucrose, succinate, pyruvate, threonine, glutamine, valine and serine were highly exchanged (average fluxes 1-5 mmol/gDW/h). So, *S. elongatus* rearranges it metabolism and exchanges to grow with both different *E. coli* strains, independent of their ability to utilize sucrose. The new Fig. 3 now shows the predicted fluxes of metabolites being exchanged (Fig. 3a). Furthermore, we moved the previously presented graphical representation of these flux distributions to Supplementary Fig. 3-4.

Of somewhat lesser importance, the context of this work was not framed in reference to the multiple studies that precede it. There are now multiple published manuscripts describing the use of this same cyanobacterial strain (S. elongatus CscB+) for the formation of co-cultures. Indeed, 3 of the 4 co-cultures (E. coli W, E. coli K-12, B. subtilis) with this strain have been previously evaluated, and a couple relevant publications of such co-cultures are not cited in this manuscript (Smith, MJ et al., ACS synthetic biology (2016); Smith, MJ et al. Biotechnology and bioengineering (2017); Weiss, TL et al., 2017 Metabolic Engineering). Without a priori knowledge of these works, it may be difficult for the reader to contextualize some of the significance of what is reported herein.

We thank the reviewer for her/his valuable comment and for highlighting previous studies describing heterotroph-autotroph platforms for bioproduction. We have now included all references suggested by the reviewer, which serve as framework for our work, especially for the heterotrophic cocultures with *E. coli W*, *E. coli* K-12, and *B. subtilis*. These previous findings support our modeling predictions about the exchange of specific metabolites (e.g. glutamate, lysine, glycolate) in microbial communities of *S. elongatus*[1]. We also included a discussion of our modeling predictions in the context of these previous findings.

Minor Points:

The significance of Figure 1A is not clear from the current format. Additionally, there are no bootstrap values shown at nodes on the current panel, as is reported in the figure legend. Based on the current formatting, this panel would be better assigned to a supplement in order to make room for more impactful information.

We have now combined Fig. 1a and Fig. 1b in a single figure. We apologize that bootstrap values were not shown and decided to delete the bootstrap sentence. Adding further information to this figure would result in figure size that is not suitable for print version. We apologize for oversizing some figures initially in our manually created PDF file. The current version of the manuscript contains the final version of Fig. 1.

In Figure 2B, it is unclear what the divided colored bars represent. Is the green vs. the other color of the bar graph meant to represent the proportion of the compound of interest (e.g., formaldehyde) that is contributed by each of the

partner species? Or merely meant to represent the abundance of each species? If the latter, is this proportion based on cell number, dry biomass, cell volume, or other?

We thank the reviewer for her/his comment and apologize for not including sufficient information in the figure caption. The color of each bar represents the proportion of the heterotrophs and *S. elongatus* based on predicted growth rates. We have now edited the caption of Fig. 2b and clarified that bar colors represent the growth rate proportion of community members. We have also shorted the caption length to 350 words to meet the journal guidelines.

It is somewhat surprising that the predicted feasible metabolites for co-cultures of K-12 is much higher than the predicted co-cultures of *E. coli* W, particularly as the primary difference between these strains is a relatively small number of genes related to sucrose catabolism. Is there insight to be gained about this based on the models, or is this likely an artifact of differences in the complexity of the relative genome scale *E. coli* models (i.e., iECW1372 vs iJO1366W)?

Metabolic networks are remarkably different across strains and sub-strains of the same bacterial genus[2]. Usually, networks acquire flux distributions within a solution space delimited by network topology and constraints, meaning that addition or deletion of reactions will change the solution space and predicted flux distributions[2,3]. We screened for differences in both *E. coli* networks and found that *E. coli* W has 171 unique reactions while *E. coli* K-12 contains 193. These metabolic differences are not only associated with sucrose uptake. They are also linked to reactions in alternate carbon metabolism, cell envelop and cofactors biosynthesis, as well as with some reactions related to alanine, arginine, proline, lysine, tryptophan, and tyrosine metabolism. It is important to highlight that the *E. coli* metabolic models used in our work have been manually curated for decades[4,5]. The *E. coli* W M-model was obtained from a previous work, in which pan and core metabolic capabilities of 55 *E. coli* species were characterized[4], while the *E. coli* K-12 model *i*ML1515 is the most curated metabolic model available to date[5].

We think that this clarification will facilitate the interpretation of our modeling results and the predicted differences in flux distributions. The revised version of the manuscript now contains an expanded explanation of why predictions made with the *E. coli* models can vary. We also included a new Supplementary Table 4, which contains all unique reactions in *E. coli* K-12 and W.

It is unclear why the metabolic models of the co-culture would predict a failure of *S. elongatus* to grow in certain pairings with *E. coli* knockouts – given that the cyanobacterium is not dependent upon heterotroph viability. For example, as reported in Figure 3B, the model appears to predict no growth of any species when *E. coli* mutants in the genes phoE and talB are used. The authors statement of line 206 “Interestingly, genetic damage in the heterotrophic partner did not significant affect the growth rate of the phototroph…” is somewhat confusing in this context. Why/when would “genetic damage” in the heterotrophic partner ever be expected to have this result? Are there insights to be gained from these false negatives or improvements that could be incorporated into the models?

We thank the reviewer for her/his comment. Metabolic models are a robust quantitative biology tool, however simulations accuracy depends on models structure (e.g. network topology and connectivity) as well as on applied constraints (e.g. uptake and secretion rates, lower and upper reaction boundaries). Knockouts simulations disrupt network connectivity, sometimes preventing growth simulation due to infeasible solutions. The complexity of CM-models generates higher infeasibility ratesthan the obtained for M-models. We apologize for the misleading sentence in line 206.

Overall, feasible solutions in metabolic models are defined by a “flux solution space”. For communities is composed by the solution space delimited for each individual metabolic network. When extreme constraints are applied, the optimization result is a growth rate of 0.0 1/h and a void flux distribution vector. In the cases of the knockouts (phoE

and talB) in the previous Fig. 3b now Fig. 4b, we removed reactions associated with the phoE and talB genes, thus, FBA predicts that growth is not possible under knockout conditions. Usually these types of predictions are associated with the maximum amount of ATP that can be produced from certain amount of resources provided to the phototroph. If this amount is less than the minimum bound of the ATP maintenance of both microorganisms, 3.5 mmol/gDW/h, there is no feasible solution. This is a very constructive comment and we have now expanded the analysis of modeling outcomes regarding metabolic adjustment of knockouts. We have now included this information into the materials and methods section. We also reworded the sentence in line 206 in a more comprehensive way.

Regarding the questions:

Why/when would “genetic damage” in the heterotrophic partner ever be expected to have this result? When model simulations predicted the growth reduction of any SPC member it is because one of the microorganisms lost a valuable metabolic function (e.g. production of high energy metabolites, tryptophan, lipids). If this function was critical for the community the active member will channel resources to its community partner. This effect is remarkably present when we study lethal knockouts but it is also possible in non-lethal knockouts.

Are there insights to be gained from these false negatives or improvements that could be incorporated into the models? Yes, false negatives point to reactions in pathways that need further manual curation in M-models. Since CM-models are highly limited by how comprehensive are M-models. M-models are the primary source of information to reconstruct community models. Fig. 3b show how M-models and CM-models were not able to accurately predict experimentally observed phenotypes. Different attempts have been achieved in the last decade to curate as much as possible M-models networks [5,6]. Several underground metabolic functions have been discovered in *E. coli* during this highly complex experimental approaches [6].

A few typos in Supplemental Figure 1 in the figure legend – most noticeably, the statement that “none of the SPC synthesized methanol and formaldehyde” disagrees with the panel and the results shown for formaldehyde production in Figure 2B.

Thanks for pointing this out. We have now corrected the caption of Supplementary Fig. 1 and proofread all the text in the Supplemental Materials.

Generally, a number of other (often small) typographical errors in the manuscript could obscure reader comprehension of the results.

We have proofread the document and fixed several typos.

Reviewer #2 (Remarks to the Author):

In the presented manuscript Zuniga et al. describe several microbial co-cultures between heterotrophs and phototrophs. They mainly used genome-scale metabolic models to evaluate the potential interactions between the

species in co-culture. Despite the interest on the topic and the novelty of the modelling methodology, there are some flaws in the way the article is described that would need to be amended before publications.

Major comments

Abstract, introduction:

-Several aspects on the text, including the title and abstract are a misleading. There is a constant reference to bioproduction, but there is no bioproduction at all in the results (just a brief quantification of metabolites in the media). The real strength of the manuscript is the analysis of co-cultures using modelling and the experimental validation of that approach. By reading the title and abstract the reader expect a completely different content. -The reader expects that all conclusions apply to the different microbial systems studied but sadly, the validations have been only carried out in *E. coli* co-culture.

We thank the reviewer for critical comments. Based in part on this comment, we have modified the manuscript including the title and abstract to avoid confusions and misleading statements about bioproduction using synthetic phototrophic communities (SPC). We agree that the terminology "bioproduction" could be misinterpreted. The revised manuscript is entitled "Designing synthetic microbial communities of heterotrophs and phototrophs for sustainable growth". The revised version of the manuscript describes all evaluated SPCs at the beginning and then explains why further validation was performed for the *E. coli*-containing SPCs.

First part of results:

This is the most important part of the paper, where the new modelling aspects are shown and validated with growth. This must be expanded, and negative results must be explained with more detail. Some comments below:
-it is a bit unclear how the parameters were calculated to correlate models and experiments, was everything done in chemostat to represent the steady state of the model? Or flask?

We thank the reviewer for her/his valuable comment which was also stated by Reviewer #1. We have now expanded several sections of the manuscript and provided a more detailed description of all our findings. We describe modeling outcomes that were confirmed experimentally as well as those predictions that could not experimentally confirmed. We agree with the reviewer that this information is of great importance and could serve as valuable dataset for the reader.

We thank the reviewer for pointing out that detailed experimental descriptions were missing in the previous version of the manuscript. All experiments were performed in flasks with continuous CO_2 supply; data used to compare with the modeling outcomes was obtained at exponential phase to mimic a pseudo steady state. We have now clarified the use of flasks in the materials and methods section.

-L85. The strains of bacillus and yarrowia must be mentioned too.
Good catch! We have included the strain information of *Bacillus* and *Yarrowia* in the different sections of the manuscript, for example in the results, methods and Fig. 1. The *Bacillus subtilis* strain was 168 and the *Yarrowia lipolytica* strain Po1g.

-L124. Why this disagreement with the model? What is the predicted metabolite exchange happening here?

We thank the reviewer the comment. Modeling predictions showed that fluxes associated with metabolic exchange by the SPC composed by *S. elongatus* cscB+ and *Y. lipolytica* were remarkably lower than the predicted for *E. coli*

SPCs. We found that the metabolic exchange of CO_2, sucrose, and oxygen was three times lower than predictions obtained for *E. coli* strains. Additionally, we observed a highly reduced exchange of ions except for the exchange of phosphate. We have now included these findings in the results section of the revised version of the manuscript. Additionally, we have also expanded Fig. 3a before 4a, including all fluxes predicted to be exchanged by all SPCs.

-L129. The authors should explain why K12 is growing, what are they exchanging? What is the prediction of the model?
We apologize for obviating vital information about metabolic exchange predictions of all SCPs. We have now rerun the models and obtained metabolic exchange simulations, expanding the content of Fig. 3a. The current version of the manuscript describes how *E. coli* K-12 is sustained by *S. elongatus* through the exchange of key metabolites (e.g. succinate, pyruvate, and serine) as previously shown in Supplemental Fig. 3-4. We found that average fluxes through exchange reactions of succinate, serine, glutamine and pyruvate in the SPC containing *E. coli* K-12 varied between 1-40 mmol/gDW/h. Additionally, fluxes two orders of magnitude smaller included exchange of valine, isoleucine, phenylalanine, formate, ethanol, asparagine, and threonine (Fig. 3a, Supplementary Fig. 3). NMR and GC-MS analysis of the coculture supernatants confirmed the presence of succinate, pyruvate, serine, as well as valine, formate, and asparagine (Supplementary Fig. 4 and new Supplementary Table 5). We have now updated the results section and figures in the manuscript to highlight these results.

- what is the volume of the pre-cultures used to inculate? How this affect growth? Are pre-cultures properly washed?
We thank the reviewer for pointing this out. We updated the materials and methods section, clarifying the inoculums concentrations (~$2.5*10^7$ of *S. elongatus* and ~ $7.5*10^6$ of heterotrophs) used during the experiments shown in Fig. 1b. This optimized inoculum ratio enabled the growth of heterotrophs better than photoautotrophs in batch cultivation, triggering the cultivation of stable cocultures that prevented the heterotroph from outcompeting *S. elongatus*. Community stability changes depending on inoculation ratios as demonstrated by Hays et al. 2017 (a former member of our team)[1,7]. Hays et al. showed that changes in those conditions could lead to different growth phenotypes of the community members. For example, when inoculum ratios were varied up to two orders of magnitude experimental outcomes benefited the most abundant member. The inoculums were properly washed and checked for contamination – we have now stated this explicitly in the text.
We have now included all details about our experimental conditions in the manuscript and they read as:
"*S. elongatus* was propagated in BG-11 (Sigma-Aldrich) plus 1 g/L HEPES, pH 8 in constant light at 35 °C. Axenic cyanobacteria were checked for contamination via plating on rich media. *B. subtilis* and *E. coli* were propagated in LB-Miller (EMD Millipore). Co-culture medium was optimized consisting of BG-11 supplemented with 106 mM NaCl, 4 mM NH4Cl and 25 mM HEPPSO, pH 8.3-KOH.
To prepare pre-cultures for *S. elongatus*, *S. elongatus* cultures at exponentially growing phase were centrifuged and washed twice with BG-11 and suspended in a proper volume of co-culture medium. To prepare pre-cultures for heterotrophic strains (*B. subtilis*, *E. coli*, and *Y. lipolytica*), single colonies were picked into LB-Miller media and grown until turbid at 37 °C. Cells were diluted into the appropriate co-culture media containing 2% sucrose to acclimate the cells to co-culture media, and maintained within log phase growth (OD600 < 0.70) before use in co-cultures. The log phase cultures were centrifuged and washed twice with BG-11 and suspended in a proper volume of co-culture medium. The inoculum volume for *S. elongatus* and the heterotrophic strains were 1 mL and 200 µL, respectively, to achieve initial cell numbers of ~$2.5*10^7$ for *S. elongatus* (OD750=0.1) and ~ $7.5*10^6$ for the heterotrophs (OD600=0.01).
Heterotrophic growth was measured by inoculating washed cells at 0.01 OD600 (bacteria) or 0.05 OD600 (yeast) into fresh co-culture media containing sucrose. Data for growth rate measurements were collected from 25 mL flask cultures while 96-well plates with 1 mL culture volumes were used to assay growth in a gradient of sucrose concentrations (0.156 mg/mL to 10 mg/mL, Fig. 2c) as well as growth in conditioned media. OD600 of plates was read on a Synergy Neo plate reader (BioTek, Winooski VT)."

- Fig 1b should also show how is the growth in g/L as it is not the same to have a very quick growth but with final biomass produced, what is especially relevant in bioproduction. The full growth curves should be shown in supplementary. How is the quantification of each cell type?

We thank the reviewer for pointing this out. Simulations obtained using M-models as well as CM-models enabled predicting rates, e.g. growth rate, uptake or secretion rates. For validation purposes we decided to display our results as rates. Biomass concentrations were used to calculate SPC-specific growth rates. Cell numbers were determined using colony-forming units (CFU) for heterotrophs and flow cytometry for the phototroph. Growth curves are now included as Supplemental Fig. 1. We have also expanded details about biomass quantification into the methods section "Strains and culturing conditions".

The added text now reads: "The *Synechococcus elongatus* biomass concentration was analyzed by a FACSCalibur flow cytometer (BD Biosciences, San Jose, CA) after adding BD Liquid Counting Beads (BD Biosciences, San Jose, CA). The absolute cell numbers in samples were determined by comparing cellular events to the counting bead events measured by the flow cytometer using the equation provided in the kit's total dissolved solids document. For the heterotrophic strains, serial dilutions were plated on LB plates to determine cell numbers. Colony-forming units (CFU) were counted after overnight's incubation at 37 °C."

Second part of results:

Here the authors are measuring the secretion of different metabolites to the media. This section is not very clear but is certainly not related to bioproduction. Firstly, the well known metabolites produced by the selected organisms such as acetone, acetoin or citric acid are not considered and only others are measured that are not really produced in relevant amounts by these organisms. Then the values of production are low, and thus, more in the range of things that are secreted or accumulated rather than things with potential for bioproduction, as it is understood in microbial biotechnology. This section is mainly talking about *E. coli* K12.

The reviewer is correct that the amounts produced are not suitable for production biotechnologically and the term bioproduction in this contecxt is misleading and does not accurately describe the focus of our research. In the revised version of our manuscript we focused on sustainable growth, describing metabolic interactions among community members and comparing the production capabilities of each phototrophic community (4 total). The response of the community networks enabled selecting for the SPC containing *E. coli* K-12 as the most promising community. Thus, further analyses were performed for the pair containing *E. coli* K-12. We have now expanded the workflow of or research clarifying when we use experimental data of all communities or only of the community containing *S. elongatus-E. coli* K-12 to confirm modeling predictions. We have also created a new Supplementary Table 5 that highlights how citrate, pantothenate, and acetone are accumulated in the supernatants of the coculture SPC *S. elongatus-E. coli* K-12.

-the 'production' is shown as mmol/gDCW/h (Fig. 2b) which are the units given by the model but not units often used in bioproduction experiments. How can you translate this to titers and yields? Is any of the metabolites actually produced in any significant amount for bioproduction? They all look very low to me.

We have expanded the results section, including calculated yields for butanal, ethanol, formaldehyde, and succinate. Average product/biomass yields varied among 0.13-0.27 g/gDW. We have now created the new Supplementary Table 3, which shows all the yields for all SPCs and metabolites in Fig. 2b. Metabolite production capabilities were performed assuming a "wild type" community network. All CM-models generated during this research will be publically available, enabling further studies to identify suitable network states (e.g. knockouts) to improve the yield

of certain metabolite. Again, the amount of products is not intended for biotechnological purpose but rather shows new avenues for metabolites, which could potentially be generated in the future.

-the metabolite exchange predicted by the model in the different scenarios are not validated, are they?

We thank the reviewer for her/his comment. We validated the metabolic exchange of SPCs growing in wild type conditions for the community of *E. coli* K-12, since this community was predicted to have the highest metabolite flux exchange (see new Fig. 3a). Overall metabolic exchange flux was up to 30 times smaller in the SPCs containing *B. subtilis* and *Y. lipolytica* (see Supplemental Table 2). We have now clarified that validation was only performed for the *E. coli* K-12 community and additional validation of rest of SPCs was out of the scope of this research. This statement is now clarified in the results section of the revised version of the manuscript.

-The text in this section is almost exclusively about *E. coli* co-cultures.
The reviewer is correct, that most of the experimental validation was performed for the SPC composed of *S. elongatus* and *E. coli* K-12. We have now clarified in the text that this particular SPC was one of the most promising since *E. coli* K-12 was able to thrive without sucrose consumption, had the highest metabolic exchange, and was predicted to have the best potential of all SPCs considered. In the revised version of the manuscript we made clear when referring to exclusive confirmation of predictions for the SPC composed by *S. elongatus* and *E. coli* K-12.

Third part of results:

-only 3/4 KO were validated experimentally, maybe more KO should be tested. Why the authors selected only 10?

We thank the reviewer for her/his comment. Gene essentiality simulations (12,893 gene knockouts) were used as a framework to track genes/gene changing their growth phenotypes while growing under mono- or coculture conditions. These simulations unraveled new phenotypes, especially for essential genes knockouts that were compensated under coculture conditions. We used the available knockouts gene library of *E. coli* K-12, which enabled us to perform a proof of concept study (instead of validating all 12,893 knockouts) of how CM-models can help to define gene essentiality in microbial communities. These prediction capabilities lead to the discovery of genes with metabolic capabilities likely to be compensated by a community partner. These genes can probably become auxotrophies throughout evolution, as often observed in stable microbial communities[8]. We performed a model-driven selection of genes maintaining monoculture phenotypes in coculture conditions (*aspC, cycA, talB, atpC, thrC, phoE*) and genes changing their phenotypes (*pheA, cysG, ilvE, ilvC*) to demonstrate the potential of CM-models to predict gene essentiality for different SPCs. This experiment was designed as a proof of concept to test changes in gene essentiality. We have now clarified the KO selection and experimental design in the methods section, which includes two treatments with four different genes with at least four replicated each. Additionally, the revised version of the manuscript includes an expanded Supplemental Table 8, showing changes in genes essentiality due to cocultivation. We have also compared our gene essentiality results with previous transposon sequencing (TN-seq) data sets published for *E. coli*[9–11]. Experimental outcomes of gene mutagenesis obtained for different microbial communities confirm our predictions in which the presence of a community partner shapes the gene essentiality of each community member. We have added a discussion of this effect in our revised manuscript.

-The validation has been done only in *E. coli*. If the authors want to claim the validation as general, other organisms should be tested too.

We apologize for statements in the results section that could have been misleading. We have fixed several sentences in the revised version of the manuscript, pointing out why experimental confirmation was performed only for the SPC composed by *S. elongatus* and *E. coli* K-12.

-Figure 3 B, why some of the co-cultures did not allow grow of *S. elongatus* in the model?

Constraint-based models depend on model structure (e.g. network topology and connectivity) and applied constraints (e.g. uptake and secretion rates, lower and upper reaction boundaries) to simulate phenotypes. When extreme constraints are applied the solution space gets reduced and optimization results are unfeasible (zero growth with a void flux distribution vector). Every knockout simulation layers an additional constraint which can affect simulation outcomes. Supplemental Table 8 shows predictions statistics, overall out of 12,893 gene knockouts predictions only 148 resulted infeasible. However, 108 are in associated with *S. elongatus* genes. Usually unfeasible predictions are associated with the maximum amount of ATP that can be produced from certain amount of resources provided to the phototroph. If this amount is less than the minimum bound of the ATP maintenance of both microorganisms, 3.5 mmol/gDW/h, there is no feasible solution. This is a very constructive comment and we have now expanded the results and methods sections to find more describing feasibility in a more comprehensive fashion.

Four part of results:

-L239. How can Fructose 6P be predicted as exchangeable when it cannot cross membranes?

We thank the reviewer for this comment. Community metabolic models rely on the creation of an artificial compartment referred to as shared metabolite pool (SMP). This compartment defines which metabolites are suitable for sharing based on experimental data. To account for as many metabolites as possible we tested the growth of the community members in isolation over 180 different carbon sources. Overall, *S. elongatus* was able to consume 52 metabolites (Supplementary Table 1), including fructose-6P. CM-models assume that when a metabolite can be consumed, it can be potentially excreted as well. Thus, metabolite exchange is only possible if both microorganisms contain the same transport reaction. In this case the models of *B. subtilis*, *E. coli* K-12 and *E. coli* W can exchange fructose 6P [5,12,13].
Previous studies had shown that engineered *S. elongatus* cscB$^+$ enhances hydrolytic capabilities, triggering glucose and fructose consumption by the endogenous sucrose invertase (putatively encoded by SYNPCC7942_0397)[14]. Our experimental results found that *S. elongatus* can uptake fructose 6-phosphate as well as D,L-alpha-glycerol-phosphate. The consumption of those metabolites enhanced growth phenotypes by more than 20±2% in comparison with a control. Currently, in a separate study we are analyzing in-depth changes in the morphology and metabolism of *S. elongatus* due to sucrose enginering (cscB$^+$). In previous studies we have noticed interesting metabolic and physiological changes in *S. elongatus* phenotypes. We thank the reviewer for pointing this out; we have now expanded the definition of SMP in the methods section clarifying how the models are built.

-L241. Confirmed again only for *E. coli*.

We have now explicitly stated for what pairs experimental confirmation and/or predictions were performed (see also comments above).

-Make a clear table with the prediction of metabolites exchanged and the validated ones by NMR or GC. Try to explain the mismatches.

We thank the reviewer for this constructive comment. We have now created a new Supplementary Table 5, showing all measurements by NMR and GC-MS. We compared all measurements under mono- and coculture conditions with modeling predictions related to metabolic exchange and intracellular production rates. We also clustered metabolic profiles, facilitating the identification of changes in metabolic profiles. We included a detailed discussion of these results in the revised manuscript.

-L261. Are the intracellular metabolites of any of the species different in co-culture than in monoculture?

A new table (Supplementary Table 5) shows an in-depth analysis of differences between intracellular and extracellular concentrations of metabolites under mono- and coculture conditions. Supplementary Table 5 also encompasses clustergrams that enable identification of metabolites that change the most based on condition. We have included measurements using NMR and GC-MS in this table as well and discussed the results in the manuscript.

-RNAseq validation only for E. coli.

We have now explicitly stated for what pairs experimental confirmation and/or predictions were performed (see also comments above).

-Table 2 NMR. Why coli is producing sucrose? More than elongatus?

We thank the reviewer for pointing this out. We used sucrose as a control in all experiments. We have now included a description of the controls in the methods section.

-Table 3 should show the extracellular metabolites too, which are more relevant for this work.

Both intracellular and extracellular samples provide insights into metabolic communication among the community partners. When comparing intracellular metabolite profiles of cultures under mono- and coculture we can identify possible intracellular bottlenecks due to accumulation. On the other hand, characterizing extracellular metabolites allows for contextualization of possible metabolic exchanges. We thank the reviewer for this comment and have made changes to report results in a more comprehensive fashion. The revised version of the manuscript shows predictions for each SPC (new Fig. 3a) as well as a complete dataset of metabolites measured by NMR and GC-MS (new Supplementary Table 5). Both analytical techniques complemented each other to characterize as many metabolites as possible, for example ethanol and formate were successfully detected using NMR but not by GC-MS. Metabolites results obtained using NMR are given in molar concentrations (uM). GC-MS data was normalized using Z-scores because raw collected data referred to metabolite abundances, which highly change from metabolite to metabolite. The use of Z-scores facilitates data interpretation as deployed in the clustergrams. The revised version of the manuscript describes these findings.

Methods:

Media composition for co-cultures is missing. Description of BG-11 could be also a good addition.

Thanks for this suggestion. The methods section was expanded and now reads as: “BG-11 medium is composed of (in 1 L): 1.5 g $NaNO_3$, 0.04 g K_2HPO_4, 0.075 g $MgSO_4 \cdot 7H_2O$, 0.036 g $CaCl_2 \cdot 2H_2O$, 0.006 g Citric Acid·H_2O, 0.006 g Ferric Ammonium Citrate, 0.001 g $Na_2EDTA \cdot 2H_2O$, 0.02 g Na_2CO_3, 1 mL BG-11 trace metals solution. The BG-11 trace metals solution (in 1 L) consists of: 2.86 g H_3BO_3, 1.81 g $MnCl_2 \cdot 4H_2O$, 0.22 g $ZnSO_4 \cdot 7H_2O$, 0.39 g $Na_2MoO_4 \cdot 2H_2O$, 0.079 g $CuSO_4 \cdot 5H_2O$, 49.4 mg $Co(NO_3)_2 \cdot 6H_2O$.”

General comments and suggestion:

I think the manuscript has potential, but it is misleading in many aspects. To me, most of the validations has been done with *E. coli* as co-culture. I think the manuscript will benefit from a clearer story if the paper is focused in this co-culture only. Maybe a short paragraph can be added at the end to say that this can be applied to any other organisms with model available and you can add in supplementary some of the modelling results you have for other microbes. On the contrary, as it is written now the authors would need to include more experimental validations for all the different microbes.

I would also focus in the novelty of this article which is the modelling and its validation. I would try to explain more how it works and how people could use it. The authors must avoid highlighting too much the bioproduction, as this work is not about bioproduction at all. The potential of this work for bioproduction can be discussed in the discussion section.

We are very thankful for the reviewers comments and have made changes throughout the manuscript accordingly. We believe that our manuscript has improved greatly due to these suggestions. The revised version of the manuscript now clarifies which modeling outcomes were confirmed experimentally and which SPC was used for validation. We have restructured the manuscript and mention the models at the beginning of the revised version. We also have clarified that only the SPC of *S. elongatus* and *E. coli* K-12 was studied in greater detail. We also added an additional paragraph to explain why this SPC was selected. Furthermore, we have modified several of the main figures and added supplemental figures and tabels analyzing the metabolomics data in-depth. Additionally, we avoided the use of the term bioproduction, since it was misleading and did not accurately reflect the work presented here.

Reviewer #3 (Remarks to the Author):

In this manuscript, the authors conduct a combined theoretical and experimental study exploring the interactions among five species: *Synechococcus elongatus*, two strains of *Escherichia coli*, *Yarrowia lipolytica*, and *Bacillus subtilis*. Flux within different defined communities are predicted using community metabolic models, and then model predictions are validated with experimental expression, KO, and metabolomics data. Community model analyses have been conducted previously within a wide range of systems to date, including studies that compare community model flux predictions with expression data. That said, this is the first study to examine these specific species and combine an autotroph with a range of defined heterotrophs. This is perhaps the first study to confirm that gene essentiality profiles are altered when species grow in community, although TN-seq data to this effect has been previously published. It would be interesting to compare these results with changes in gene essentiality when growing in rich media vs minimal media. One would expect changes to be similar. The metabolomics data is somewhat new as a validation of interactions in community models, although the data as currently presented seems unclear and inconclusive. The authors' logic in explaining what changes are occurring and why needs to be clarified. Overall, the work is an interesting demonstration and validation of community metabolic models, but beyond this it's unclear what new insights have been revealed by the work. A deeper message may be here, but unfortunately, the paper has many grammatical and organizational issues that need to be resolved before it can be fully understood by readers.

We would like to thank the reviewer for the constructive comments. The revised version of our manuscript has improved greatly in structure and content, including more literature information about changes in gene essentiality as well as additional experimental data. Additionally, we have included experiments that compare the growth phenotypes in minimal medium (BG-11) with the ones obtained in rich medium. We found that growth rates of the community almost doubled because of the nutrients present in LB media (41±11%). Interestingly, we observed syntrophic interactions even though *E. coli* knockouts could thrive in rich media under monoculture conditions.

We also thank the reviewer for pointing us to the TN-seq experiments. Experimental outcomes of gene mutagenesis obtained for different microbial communities confirm our predictions in which the presence of a community partner shapes the gene essentiality of each community member. We have included a detailed discussion of this effect in our manuscript.

My current significant concerns include:

1.) On line 105 the authors say: "The modeling compartment Shared Metabolite Pool (SMP) was manually curated in all CM-models, including experimental and genetic evidence of metabolic exchange capabilities by each community member." How different were the metabolite exchange constraints in the community models from the transport reactions built into the original isolate species models? Were more exchanges added based on experimental evidence? Were some removed?

This is an excellent comment. Community metabolic models rely on the creation of an artificial compartment referred to as shared metabolite pool (SMP). This compartment defines which metabolites are suitable for sharing based on experimental data obtained using isolates. We performed a high-throughput growth essays for *S. elongatus* in monoculture, testing over 180 different carbon sources of which *S. elongatus* was able to consume 52 metabolites (Supplementary Table 1). Similar essays had been previously performed for the heterotrophs *B. subtilis*, *E. coli* K-12, *E. coli* W, and *Y. lipolytica*. This information was included into the M-models by manual curation, adding exchange and transport reactions from the extracellular space to the cytoplasm. These transport reactions were used as a template to create the compartment SMP by adding an additional group of reactions that connects the

extracellular space with the SMP and then with the cytoplasm of each microorganism. By carefully adding reactions, all metabolite consumption capabilities were included in all the CM-models. Metabolite exchange is therefore only possible if both microorganisms contain the same transport reaction (see Fig. 2c-d).
The complete dataset of consumed metabolites by all community members under monoculture conditions is given in Supplementary Table 1. We thank the reviewer for pointing this out; we have now expanded the definition of SMP in the methods section clarifying how the CM-models were built.

2.) Can the authors explain why the community models predict increased growth in *S. elongatus* when cocultured with *Y. lipolytica*?

We thank the reviewer for her/his comment. To simulate community growth we optimize for the biomass reaction of each community member. Briefly, we use OptCom[15], an FBA-associated algorithm that allows the optimization of multiple objective functions at the same time considering thermodynamic/capacity and mass constraints (lb, ub, and biomass stoichiometric coefficients). The overall community growth rate is determined by adding individual growth rates of all community members. The predicted growth rates of each community member depends on the robustness of its own network and how microbes split resources provided in the culture medium as well as exchange mass fluxes. For the SPC of *S. elongatus* and *Y. lipolytica*, we found that the exchange of CO_2 and O_2 among community member is remarkable lower than for other SPCs (e.g. *S. elongatus-E. coli*), reducing the growth yields. Our new Fig. 3a also shows that *Y. lipolytica* has a very low exchange of potassium, glycolate, and certain amino acids, such as threonine, isoleucine, and glutamine. These changes in predicted flux distributions can be the main reasons why *S. elongatus* can achieve higher growth rates than *Y. lipolytica*. We now highlight these findings in the new version of the manuscript.

3.) How were CM-model growth rates predicted? What was the objective function? The authors mention using the COBRA toolbox community simulation tools in the methods, but then explain very little about how the tools work. This relies on the reader to have more expertise in COBRA community models than will be typical, so it is recommended that the methods describe how the community models work in a little more detail.

The revised version of the manuscript now contains a detailed description of the tools and algorithms used to simulate the model. We have also described the function of the biomass objective function and how it helps to accurately simulate growth of individual members in a community.

The expanded text in the methods section now reads:

CM-models were reconstructed using M-models of *S. elongatus PCC7942*, *i*JB792[16], *B. subtilis*, *i*YO844 , *Y. lipolytica*, *i*Yali4[17], *E. coli* W and K-12, *i*ECW1372 and iJO1366[2,18]. Additional information about the models is shown in Table 1. All models were subjected to quality control and assessment tests (QC/QA) and integrated as CM-models using the COnstraint-Based Reconstruction and Analysis (COBRA) Toolbox[19]. The COBRA Toolbox is a MATLAB (MathWorks Inc., Natick, MA) software suite for quantitative prediction of cellular and multicellular biochemical networks, implementing the most comprehensive collection modelling methods and algorithms to perform high-throughput model-driven analysis[19].CM-models benchmarking was performed using the Gurobi Optimizer Version 5.6.3 solver (Gurobi Optimization Inc., Houston, Texas) in the COBRA Toolbox[19] for MATLAB. We simulated the maximal growth rate of the community members using the Flux Balance Analysis (FBA) associated algorithm OptCom[19]. This algorithm maximizes for the biomass objective functions or biomass reactions, which contain all metabolites that comprise organism biomass (e.g. carbohydrates, lipids, proteins).

4.) The expression flux comparison method could use much better description. For example, is the individual cell or community model growth used to normalize fluxes in equation 1? The notation in these equations is generally not

well described. For another example, it's not at all clear what this sentence is trying to say: "Snk,j deploys pathways usage as syntrophically active, neutrally active, and deactivated."

We thank the reviewer for her/his comment. We use the member-specific predicted growth rate to normalize fluxes in Equation 1. We have now carefully described all components in Equations 1-5 to provide more detail.

5.) On line 138, the authors state: "Metabolite production feasibility simulations were combined with biomass growth maximization of both community members. When the metabolite production rate and growth of both community members were higher than zero, the tested metabolite was counted as feasible (Fig. 2a)." This implies that maximum growth was computed first, then the authors attempted to produce the metabolites? So if a metabolite decreased the growth yield, it's production would be reported as zero? Is this correct?

Yes, that's correct. We used the OptCom algorithm to compute only growth rates at first and then we added as constrain the generation of metabolites at increased production rates (x-axis Fig. 2c-d). Phase planes show how increased production rates reduce the growth of the community members, due to shifting of resources from biomass production to metabolites synthesis. We apologize that this fact was not clear in the previous version of the manuscript and have now included additional text to state this fact.

Also, when you say the biomass growth of both members was maximized, did you maximize the sum of the biomass or both species? Or maximize one species first, then maximize the other? Order would matter in that case. Also, how did community production profiles compare with individual species production profiles for each species?

OptCom has the capability to maximize for several biomass objective functions at the same time. Thus, we can predict microorganism-specific growth rates within the microbial community. The overall biomass growth rate is given by the sum of each community member growth rate. Growth profiles were calculated for mono- and coculture conditions. The new Supplementary Table 3 shows the growth and production rates as well as the overall yield. Overall, we found that *B. subtilis* cannot produce ethanol, succinate, butanal, or formaldehyde in monoculture conditions. We also found that *E. coli* W and *Y. lipolytica* can produce a lower amount of these metabolites (~10-30% less) under monoculture conditions compared to coculture growth. Similar predictions were obtained for *E. coli* K-12. Except for succinate production, which it was higher under monoculture. We thank the reviewer for this very constructive comment and have now expanded the results and methods sections.

6.) How do community knockout results compare with individual species growing in very rich media?

We performed an additional experiment to examine the mono- and coculture growth phenotypes in rich media (80% LB + 20% BG-11). We used a LB:BG-11 mix because salts present in BG-11 are necessary for the cyanobacteria to grow. Interestingly, the SPC maintained syntrophic interactions despite of the capability of *E. coli* to thrive in LB while having lethal knockouts. We found that rich media increased the community growth rate by 41±11% in many of the KO cocultures. We also observed that the growth rate of *E. coli* K-12 wild type and S. elongatus cscB+ achieved under mono- and coculture conditions are significantly different (t-test, p-value<0.05, n=3), being the coculture LB growth rate up to 15% higher than in BG-11 (data shown in Fig. 4). We have now generated a new Supplementary Fig. 6 with all the outcomes obtained for all KOs while cultured in rich and minimal medium. We have also modified accordingly the methods and results sections in the main text.

7.) Could the thrC results have to do with regulation of threonine transporters in the donor or receiver strains? If this mutant is simply grown in a very rich media, is it viable?

We thank the reviewer for his comment. As he/she pointed out *thr*C growth phenotypes depend on culture conditions. For example, there is experimental evidence that under monoculture conditions *thr*C can achieve positive growth phenotype, while cultured in rich medium such as Luria-Bertani (LB) enriched, LB Lennox, and LB broth but deployed lethal growth phenotypes in minimal media such as M9 medium with 0.4% glucose, M9 medium with 1% glycerol, and MOPS medium with 0.4% glucose. In our new experiment we found that indeed, the *thr*C strain grew as well as the other mutants and wildtype in very rich media (LB). We also found that both rich and minimal media also rescue the *thr*C knockout in coculture. We have now updated Fig. 4 and Supplementary Table S9. Model simulations associated with *thr*C showed higher flux exchange of threonine, increasing from 1.6 mmol/gDW/h to 3.07 mmol/gDW/h. The reasons for differences between growth predictions and experiment may be also due to the absence of the complete *thr*C reaction pathway. This gene catalyzes reactions associated with serine conversion into 3-chloroalanine and pyruvate as well as vinylglycine synthesis, an intermediary in thymidylate synthase activity. Reactions associated with 3-chloroalanine and vinylglycine are out of the scope of the *E.coli* K-12 *i*ML1515 model, which likely explains the inaccuracy of predicted growth of the *thr*C knockout (KO).

8.) The higher levels of certain metabolites does not necessarily indicate the presence of metabolic exchange within the co-cultures. Can the authors explain the logic of this analysis further? Were the metabolite level increases statistically significant? How much higher were the levels in co-culture? This isn't totally clear when only Z-scores are provided.

This is an excellent point. We agree with the reviewer, the presence of metabolites does not indicate metabolic exchange necessarily. However, to date there is no suitable experimental technique that can resolve metabolic exchange quantitatively[20], thus, in this research we used target metabolomics data of both intracellular and extracellular samples to get insights into metabolic communication among the community partners. When comparing intracellular metabolite profiles of cultures under mono- and coculture we can identify possible intracellular bottlenecks due to accumulation. On the other hand by characterizing extracellular metabolites we can contextualize possible metabolic exchanges. We apologize if our previous version of the manuscript did not relate the results in a comprehensive fashion. The revised version of the manuscript shows predictions for each SPC (new Fig. 3a) as well as a complete dataset of metabolites measured by NMR and GC-MS (new Supplementary Table 5). Both experimental techniques resulted complementary to characterize as many metabolites as possible, for example ethanol and formate was successfully detected using NMR but not GC-MS. These experimental drawbacks about obtaining complete datasets limit our capabilities to perform statistical comparisons (see new Supplementary Table 5). Additionally, metabolites results obtained using NMR are given in molar concentrations (uM) and GC-MS as abundances with values up to two orders of magnitude higher. Calculation of Z-scores of GC-MS data helped to visualize these experimental outcomes, which highly change from metabolite to metabolite. The use of Z-scores facilitates data interpretation as deployed in the clustergrams. The new version of the manuscript describes our findings regarding metabolic exchange and experimental validation. We expanded Figures and text as well as we also included new Supplementary materials.

Minor comments:

The manuscript includes numerous grammatical errors and sentences that are difficult to understand in their present state. As such, it could use a very thorough edit for understandability and grammar.

We thank the reviewer for the comment. We have fixed several typographical errors and have edited the text to improve understandability.

References

1. Smith, M. J. & Francis, M. B. A designed *A. vinelandii – S. elongatus* coculture for chemical photoproduction from air, water, phosphate, and trace metals. *ACS Synth. Biol.* **5**, 955–961 (2016).
2. Monk, J. M. *et al.* Genome-scale metabolic reconstructions of multiple *Escherichia coli* strains highlight strain-specific adaptations to nutritional environments. *Proc. Natl. Acad. Sci.* **110**, 20338–20343 (2013).
3. Orth, J. D., Thiele, I. & Palsson, B. Ø. What is flux balance analysis? *Nat. Biotechnol.* **28**, 245–248 (2010).
4. Feist, A. M. *et al.* A genome-scale metabolic reconstruction for *Escherichia coli* K-12 MG1655 that accounts for 1260 ORFs and thermodynamic information. *Mol. Syst. Biol.* **3**, 121 (2007).
5. Monk, J. M. *et al.* *i*ML1515, a knowledgebase that computes *Escherichia coli* traits. *Nat. Biotechnol.* **35**, 904–908 (2017).
6. Guzmán, G. I. *et al.* Model-driven discovery of underground metabolic functions in *Escherichia coli*. *Proc. Natl. Acad. Sci.* **112**, 929–934 (2015).
7. Hays, S. G., Yan, L. L. W., Silver, P. A. & Ducat, D. C. Synthetic photosynthetic consortia define interactions leading to robustness and photoproduction. *J. Biol. Eng.* **11**, 4 (2017).
8. Embree, M., Liu, J. K., Al-Bassam, M. M. & Zengler, K. Networks of energetic and metabolic interactions define dynamics in microbial communities. *Proc. Natl. Acad. Sci.* **112**, 15450–15455 (2015).
9. Ibberson, C. B. *et al.* Co-infecting microorganisms dramatically alter pathogen gene essentiality during polymicrobial infection. *Nat. Microbiol.* **2**, 17079 (2017).
10. Morin, M., Pierce, E. C. & Dutton, R. J. Changes in the genetic requirements for microbial interactions with increasing community complexity. *Elife* **7**, (2018).
11. Lewin, G. R., Stacy, A., Michie, K. L., Lamont, R. J. & Whiteley, M. Large-scale identification of pathogen essential genes during coinfection with sympatric and allopatric microbes. *Proc. Natl. Acad. Sci.* **116**, 19685–19694 (2019).
12. Dietz, G. W. & Heppel, L. A. Studies on the uptake of hexose phosphates. II. The induction of the glucose 6-phosphate transport system by exogenous but not by endogenously formed glucose 6-phosphate. *J. Biol. Chem.* **246**, 2885–90 (1971).
13. Henry, C. S. *et al.* High-throughput generation, optimization and analysis of genome-scale metabolic models. *Nat. Biotechnol.* **28**, 977–982 (2010).
14. McEwen, J. T., Machado, I. M. P., Connor, M. R. & Atsumi, S. Engineering *Synechococcus elongatus* PCC 7942 for continuous growth under diurnal conditions. *Appl. Environ. Microbiol.* **79**, 1668–1675 (2013).
15. Zomorrodi, A. R. & Maranas, C. D. OptCom: a multi-Level optimization framework for the metabolic modeling and analysis of microbial communities. *PLoS Comput. Biol.* **8**, e1002363 (2012).
16. Broddrick, J. T. *et al.* Unique attributes of cyanobacterial metabolism revealed by improved genome-scale metabolic modeling and essential gene analysis. *Proc. Natl. Acad. Sci.* **113**, E8344–E8353 (2016).
17. Kerkhoven, E. J., Pomraning, K. R., Baker, S. E. & Nielsen, J. Regulation of amino-acid metabolism controls flux to lipid accumulation in *Yarrowia lipolytica*. *npj Syst. Biol. Appl.* **2**, 16005 (2016).
18. Orth, J. D. *et al.* A comprehensive genome-scale reconstruction of *Escherichia coli* metabolism. *Mol. Syst. Biol.* **7**, 535 (2011).
19. Schellenberger, J. *et al.* Quantitative prediction of cellular metabolism with constraint-based models: the COBRA Toolbox v2.0. *Nat. Protoc.* **6**, 1290–1307 (2011).

20. Zuñiga, C., Zaramela, L. & Zengler, K. Elucidation of complexity and prediction of interactions in microbial communities. *Microb. Biotechnol.* **10**, 1500–1522 (2017).

Reviewers' comments:

Reviewer #1 (Remarks to the Author):

The revised manuscript of Zungia et al., is substantially improved in language and presentation. Some of the fundamental insights that are gained through the flux analysis are more clearly stated in the document. Many of my original concerns have been addressed, though I point out a few additional considerations that may further improve reader comprehension below.

Comments:

Line 280 is unclear: " This prediction was validated experimentally, where the heterotrophic partners improved resistance to S. elongatus knockouts (7-27%) under cocultivation." This statement appears to say that the authors experimentally validated some set of S. elongatus knockouts that were predicted to be lethal in mono-culture and found that they were rescued in growth in co-culture. This would be a technically impressive feat and would be of considerable interest to the subject of the manuscript, but I can not find any data in Figure 4, or elsewhere, that relates to this statement. (e.g., Fig 4a appears to relate to analysis of simulations, not live cultures). Please revise accordingly.

Related to this point, the manuscript is significantly improved in reader comprehension, but there continues to be some difficulties at various points when the reader is attempting to determine if a given statement or figure panel relates to simulations or experimental data. The authors are to be commended in validating a number of the simulation predictions, but further clarifying when they are referencing simulations vs. experimental (or both) in main text and figure legends would assist the ease of reading. Generally, such ambiguities tend to be clustered towards the later analyses (Figures 3 and 4 and associated Supplemental information)

In the proofs of the Supplemental Information, Figure S2 has text with insufficient resolution to read – even with digital zoom. Likewise, a list somewhere in the manuscript/Supplemental of all of the abbreviations used (e.g., for the various metabolites) and their full names would be helpful.

Minor comments:

Thylakoid transport reactions are lumped together as one of the dominant fluxes that are predicted to constrain community growth parameters by the community models. Because it is unclear what all is included as part of "thylakoid transport" (e.g., line 250), it is hard to know if this might represent a unexpected insight, or merely a more obvious conclusion (i.e., if the model were allowed more active photosynthetic processes, a higher total biomass would be achieved). I was unable to immediately locate all of the reaction fluxes categorized as "thylakoid transport" even in the Supplemental Files. A small clarification in text and/or Supplemental might help the reader to distinguish between these possibilities.

It is curious to see the unusually high discrepancy between anticipated growth rates of S. elongatus CscB in co-culture with Y. liptolytica and the actual growth rate. It appears that this discrepancy is mostly driven by a predicted large growth increase in S. elongatus that is not realized in real cultures. Is there a small set of predicted fluxes from Y. litolytica ◊ S. elongatus that drives the predicted growth enhancement? While beyond the scope of this manuscript, determining heterotroph-to-autotroph fluxes such as these that may be growth promoting could have broader implications for the stability of such synthetic co-cultures.

Reviewer #2 (Remarks to the Author):

The authors have revised the manuscript and applied part of my suggested comments. The additional tables containing previously missing data will definitely increase the visibility and impact of the article. However, I still have some major concerns about the way the results are presented.

Overall, I think the authors made a good job of analyzing the co-culture between cyanobacteria and E. coli. They combined modeling and experiment and the predictions were validated in most cases. One main conclusion of this work is how complex communities are and how with our current technical limitations we are unable to properly understand metabolite exchange. This modeling approach can become a good tool to explore this or even be used to improve our technical capabilities. However, it is clear that the experimental validations only explain partially the results of the model. That must be taken into account and, unfortunately, narrow down the applications of such a model, as if for example, it is used in order co-cultures, new experimental validations will be required, and for some organisms, there are no resources available like the knockout collections.

All this make this paper a valuable piece of work of high quality for the main co-culture investigated in detail and the major conclusions should refer to this. On the contrary, when adding other co-cultures, no studied in detail and with partial or weak explanations for many obtained results, the quality of the manuscript decreases as well as the generalist conclusions.
I recommend the authors re-organize the manuscript and present it in a different way taking to a side/ supplementary the other co-cultures. The current format makes the manuscript uncomplete and it makes the reader wonder the general validity of the conclusions.
Even when the authors claim in the response to reviewers that they have explained well why they only move forward with one co-culture, this is in my opinion neither achieved nor justified in the current version. For example, the abstract is still very misleading as by reading it you still have the impression that this is a generic article and that all the experiments have been done for the 4 co-cultures mentioned there. Again, starting by saying that this article is about 4 co-cultures is impairing the quality of the work, as most of the article is about 1. The article would benefit with a clear linear story on the chosen co-culture and presenting, if so, the other three as an additional or side study.

Figure 2B and the associated text (146-154) is a bit random, it is not clear why only those metabolites where selected, especially now that the reference to bioproduction as been removed. I am not sure this fit in the main text, as the conclusions of the study are also not adding new knowledge. I would move this out to supplementary as take the reader off the main conclusions and results of the work.

The fact that the work has been done in flask (Batch), in my opinion, make the work more difficult to replicate. How did the authors calculate growth rate? Looking at the curves, the growth often looks more linear than exponential.

The authors used Yarrowia Po1g, but this strain is auxotroph so it should not grow on the media where it is shown growth (it is unable to produce some vitamins and Uracil, among others)(probably one of those has been recovered while expressing the SUC gene, but what about the others?). This strain suffered several rounds of mutations, which makes it genome different from the original wild type strain W29. What is the genome used to generate the model used here? Maybe some of these differences are responsible for the inaccuracies between the prediction and the experiments.

Regarding the secretion of fructose 6P, I appreciate it can be taken up, however, it is known that metabolism has evolved to differentially transport things in and out, in order to keep valuable molecules in (such fructose, whose excess is not released to the media but used to build sugar storage molecules). This particular example makes me wonder if the general rule assumed by the authors where "CM-models assume that when a metabolite can be consumed, it can be potentially excreted as well" is responsible for inaccuracies and predictive errors. Such a rule should be reconsidered taking into account biological pieces of evidence and our knowledge on transporter and metabolism. This can be easily done by controlling the boundaries of the transport reaction to take place only in the right direction.

Reviewer #3 (Remarks to the Author):

The authors have responded well to all of my comments, and generally the manuscript seems much improved. In examining the results with the improved clarity, a few questions persist around Figure 4b. Specifically, when the authors refer to E. coli K12 coculture (the orange bars) in this figure, can this coculture be explained further? The text doesn't seem to mention this co-culture at all, just the elongatus coculture. I assume the E. coli K12 coculture involves growing the mutant strain in coculture with the wild-type strain? Is that the case?

Also, in Figure 4b, the E. coli coculture model results for cycA don't make sense to me. Here, you are predicting that the monoculture mutant will grow, but the co-culture will not. From what I know about community modeling, this is not expected to happen. When you expand a monoculture model to a coculture model, growth should generally stay similar or go up. It's very unexpected for growth to go away entirely. I could be misunderstanding what the E. coli coculture is, but I don't see why a model that is growing on it's own would suddenly stop growing in co-culture. Is this a mistake? Can this be explained further. Generally methods descriptions for this specific study could use more fleshing out.

Response NCOMMS-19-28644A

Reviewer #1 (Remarks to the Author):

The revised manuscript of Zungia et al., is substantially improved in language and presentation. Some of the fundamental insights that are gained through the flux analysis are more clearly stated in the document. Many of my original concerns have been addressed, though I point out a few additional considerations that may further improve reader comprehension below.

We'd like to thank the reviewer for her/his time and effort taken to review our paper as well as for the valuable comments. We have addressed all the comments, see below.

Comments:

Line 280 is unclear: " This prediction was validated experimentally, where the heterotrophic partners improved resistance to S. elongatus knockouts (7-27%) under cocultivation." This statement appears to say that the authors experimentally validated some set of S. elongatus knockouts that were predicted to be lethal in mono-culture and found that they were rescued in growth in co-culture. This would be a technically impressive feat and would be of considerable interest to the subject of the manuscript, but I can not find any data in Figure 4, or elsewhere, that relates to this statement. (e.g., Fig 4a appears to relate to analysis of simulations, not live cultures). Please revise accordingly.

We thank the reviewer for pointing this out and we apologize for this potentially misleading statement. We have now reworded this paragraph, clarifying that only some of the *E. coli* knockouts were tested experimentally. We have also edited the caption of Figure 4a, explaining that the bars represent the number of genes with resistant or lethal phenotypes. Those phenotypes were determined based on model simulations for all genes in the model.

Related to this point, the manuscript is significantly improved in reader comprehension, but there continues to be some difficulties at various points when the reader is attempting to determine if a given statement or figure panel relates to simulations or experimental data. The authors are to be commended in validating a number of the simulation predictions, but further clarifying when they are referencing simulations vs. experimental (or both) in main text and figure legends would assist the ease of reading. Generally, such ambiguities tend to be clustered towards the later analyses (Figures 3 and 4 and associated Supplemental information).

We thank the reviewer for the comment. We have now further clarified when the results refer to simulations or experimental data, and paid special attention to results represented in Figure 3 and 4 as well as in the Supplementary information. We have also proof read the entire manuscript, ensuring that model simulations and experimental data are always well defined. Text changes are shown in yellow across the main manuscript and supplemental files.

In the proofs of the Supplemental Information, Figure S2 has text with insufficient resolution to read – even with digital zoom. Likewise, a list somewhere in the manuscript/Supplemental of all of the abbreviations used (e.g., for the various metabolites) and their full names would be helpful.

Thank you for catching this. We have re-organized the figure and also increased the font size. The caption of the new version of Figure S2 also contains the full names of all metabolites in the clustergrams.

Minor comments:

Thylakoid transport reactions are lumped together as one of the dominant fluxes that are predicted to constrain community growth parameters by the community models. Because it is unclear what all is included as part of “thylakoid transport” (e.g., line 250), it is hard to know if this might represent a unexpected insight, or merely a more obvious conclusion (i.e., if the model were allowed more active photosynthetic processes, a higher total biomass would be achieved). I was unable to immediately locate all of the reaction fluxes categorized as “thylakoid transport” even in the Supplemental Files. A small clarification in text and/or Supplemental might help the reader to distinguish between these possibilities.

We thank the reviewer for her/his valuable comment. We included a clarification in the text and also expanded Supplemental Table S7, showing all reactions by compartment to facilitate easier interpretation of the results.

It is curious to see the unusually high discrepancy between anticipated growth rates of S. elongatus CscB in co-culture with Y. lipolytica and the actual growth rate. It appears that this discrepancy is mostly driven by a predicted large growth increase in S. elongatus that is not realized in real cultures. Is there a small set of predicted fluxes from Y. lipolytica ◊ S. elongatus that drives the predicted growth enhancement? While beyond the scope of this manuscript, determining heterotroph-to-autotroph fluxes such as these that may be growth promoting could have broader implications for the stability of such synthetic co-cultures.

This is a great comment. Our CM-model *Y. lipolytica-S. elongatus* encompass the current knowledge of metabolic reactions at the genome level. When we analyzed the predicted flux distributions in this CM-model, we found that *S. elongatus* efficiently uptakes “light” and CO_2 but does not channel enough resources to *Y. lipolytica*. There are major processes inside the cell, such as replication, translation, as well as various inhibitions (due to regulation and small molecule production) that are currently out of the scope of metabolic models. However, these inaccurate predictions often lead to model-driven discoveries, such as underground metabolism (1). We appreciate the reviewer’s curiosity and we plan to follow up on these aspects in the future.

Reviewer #2 (Remarks to the Author):

The authors have revised the manuscript and applied part of my suggested comments. The additional tables containing previously missing data will definitely increase the visibility and impact of the article. However, I still have some major concerns about the way the results are presented. Overall, I think the authors made a good job of analyzing the co-culture between cyanobacteria and E. coli. They combined modeling and experiment and the predictions were validated in most cases. One main conclusion of this work is how complex communities are and how with our current technical limitations we are unable to properly understand metabolite exchange. This modeling approach can become a good tool to explore this or even be used to improve our technical capabilities.

We thank the reviewer for these comments and acknowledgment of our efforts to address previous concerns.

However, it is clear that the experimental validations only explain partially the results of the model. That must be taken into account and, unfortunately, narrow down the applications of such a model, as if for example, it is used in order co-cultures, new experimental validations will be required, and for some organisms, there are no resources available like the knockout collections. All this make this paper a valuable piece of work of high quality for the main co-culture investigated in detail and the major conclusions should refer to this. On the contrary, when adding other co-cultures, no studied in detail and with partial or weak explanations for many obtained results, the quality of the manuscript decreases as well as the generalist conclusions.
I recommend the authors re-organize the manuscript and present it in a different way taking to a side/ supplementary the other co-cultures. The current format makes the manuscript uncomplete and it makes the reader wonder the general validity of the conclusions. Even when the authors claim in the response to reviewers that they have explained well why they only move forward with one co-culture, this is in my opinion neither achieved nor justified in the current version.

We highly appreciate the reviewer's comments and argument for re-structuring our manuscript and reducing the main text to only the *E. coli-S. elongatus* coculture. However, after careful consideration we respectfully disagree with the reviewer's assessment and thus have decided to keep the content of the original manuscript, i.e. describing all coculture models in the main text. The reason for this is that modeling results for all coculture models further support our findings and highlight the applicability of the approach to other systems. We also think that the inclusion of all models will increase the visibility and impact of our manuscript – in particular because systems biology of microbial communities is an emergent field, and the work presented here can and will have implications for researchers from other fields. We anticipate that our results will be of great interest to a wide audience and researchers from a broad range of different disciplines, spanning from ecology and microbiology, to molecular biology and evolution, to systems biology, metabolic engineering, and quantitative biology. Another benefit of showcasing model outcomes for all communities, instead of "burying" them in the supplement, is the release of these four community models. Researchers working for example on yeast could readily adjust these models to their particular organism and generate hypothesis about microbial interactions in their system.

For example, the abstract is still very misleading as by reading it you still have the impression that this is a generic article and that all the experiments have been done for the 4 co-cultures mentioned there. Again, starting by saying that this article is about 4 co-cultures is impairing the quality of the work, as most of the article is about 1. The article would benefit with a clear linear story on the chosen co-culture and presenting, if so, the other three as an additional or side study.

We thank the reviewer for pointing this out and we apologize for the misleading statement in the abstract. We have redrafted this section, highlighting that experimental validation was only performed for the coculture of *S. elongatus-E. coli*. We have also adjusted the number of words to 149 total (previous version contained 161 words) to meet the journal guidelines (150 words).

Figure 2B and the associated text (146-154) is a bit random, it is not clear why only those metabolites where selected, especially now that the reference to bioproduction as been removed. I am not sure this fit in the main text, as the conclusions of the study are also not adding new knowledge. I would move this out to supplementary as take the reader off the main conclusions and results of the work.

Thank you for catching this and apologize for the lack of context in this analysis. We have now added background about the choice of metabolites we focused on in this study into the main text.

The fact that the work has been done in flask (Batch), in my opinion, make the work more difficult to replicate. How did the authors calculate growth rate? Looking at the curves, the growth often looks more linear than exponential.

We agree that batch cultures are not as controlled as chemostat experiments – but this is likely true for the majority of microbiological data in the literature. Of course our work can be extended to chemostat cultures in the future, but this is out of scope for the present manuscript. The reviewer, however, addressed an important question regarding the growth curves (shape). We would like to point out that all cocultures were monitored for 72 h to enable comparison of all SCPs. Individual isolates where grown individually and then mixed, which might contribute to the effect. Supplemental Fig. 1 shows that most of the growth measurements follow a lag, log and stationary phase trajectory, except for E. coli W. Also, we would like to point out that originally we explored other methods to measure growth rates in cocultures. These methods included e.g. qPCR or shotgun sequencing, but none of them were as accurate as the plate counts, likely because they measure relict DNA. Overall, we are confident that our measurements enable accurate determinations of growth rate based on the difference in the number of cells over time (μ=(ln(OD_f)-ln(OD_o)/t). We now include this clarification in the caption of the Supplemental Fig. 1.

The authors used Yarrowia Po1g, but this strain is auxotroph so it should not grow on the media where it is shown growth (it is unable to produce some vitamins and Uracil, among others)(probably one of those has been recovered while expressing the SUC gene, but what about the others?). This strain suffered several rounds of mutations, which makes it genome different from the original wild type strain W29. What

is the genome used to generate the model used here? Maybe some of these differences are responsible for the inaccuracies between the prediction and the experiments.

We thank the reviewer for the comment. We agree with the reviewer about variances between Yarrowia Po1g and W29 metabolism and we therefore adapted the model to account for these differences. As the reviewer pointed out *Y. lipolytica* Po1g has relevant properties at genome level (MATa, leu2-270, ura3-302::URA3, xpr2-3). This genotype results in leucine auxotrophy and sucrose uptake – not in uracil auxotrophy (2). We performed strain cultivation and maintenance as suggested by Yeastern Biotech Co (Taipei, Taiwan). After a careful contextualization of genotypic traits we found that leu2-270 was linked to the reaction 3-isopropylmalate dehydrogenase that involves the metabolite (2R,3S)-3-isopropylmalate. Unfortunately this metabolite is not present in the M-model (*i*Yali4) and thus in *i*CZ-Se-Yl(1686). Interestingly, to bypass this trait we found that model simulations predicted an active exchange of isoleucine among community members (flux 0.01 mmol/gDW/h). Active isoleucine exchange can potentially overcome leucine auxotrophy by activating the valine, leucine and isoleucine degradation pathway.

Regarding the genetic modification ura3-302::URA3 associated with orotidine 5'-phosphate decarboxylase reaction: When the *S. elongatus*-*Y. lipolytica* model carries flux through this reaction (flux 0.0072 mmol/gDW/h) the growth of both microorganisms is feasible (*S. elongatus* growth rate 0.061 1/h and *Y. lipolytica* 0.0361 1/h). In case the flux through this reaction is set to zero, only *S. elongatus* is able to grow, confirming the lack of uracil auxotrophy in Po1g. The MATa and xpr2-3 genotypic traits are associated with genes of non-metabolic-activity; thus simulation of these traits is out of the scope of M-modeling. We have now included this clarification in the methods section of our manuscript.

Our CM-model, *i*CZ-Se-Yl(1686), encompass the current knowledge of metabolic reactions at the genome level. There are major processes inside the cell, such as replication, translation, that are out of the scope of metabolic modeling. Disagreement of experimental outcome and model predictions can for example be due to transcriptional/translational regulation or allosteric inhibition at the enzyme level. While predicting these aspects is a general shortcoming of metabolic modeling, false predictions can also lead to model-driven discovery, such as underground metabolic functions (1).

Regarding the secretion of fructose-6P, I appreciate it can be taken up, however, it is known that metabolism has evolved to differentially transport things in and out, in order to keep valuable molecules in (such fructose, whose excess is not released to the media but used to build sugar storage molecules). This particular example makes me wonder if the general rule assumed by the authors where "CM-models assume that when a metabolite can be consumed, it can be potentially excreted as well" is responsible for inaccuracies and predictive errors. Such a rule should be reconsidered taking into account biological pieces of evidence and our knowledge on transporter and metabolism. This can be easily done by controlling the boundaries of the transport reaction to take place only in the right direction.

We agree that controlling boundaries in the models is something easy to perform. However, collecting high-throughput phenotypic and physiological data about metabolite secretion is currently extremely challenging. First, because microbes adjust their metabolism and secretion depending on culture conditions (e.g. monoculture vs coculture, culture media, ions presence, etc.) (3). Second, designing defined experimental conditions to measure secretion of metabolites is also difficult since previous findings had shown that microorganisms in isolation as well as in microbial communities can adjust their metabolism, flux distribution and metabolic exchange in response to nutrient starvation or specific genotypic traits (4, 5). There are intrinsic limitations to assign metabolites to a defined producer in microbial communities. Recent studies have determined secretion under monoculture condition and inferred that the same metabolites are secreted in a microbial community (5). However, we have recently shown that metabolic exchanges can be highly dynamic in microbial communities (4). Therefore, we do not define a predetermined set of metabolites for exchange in our approach! We manually curate all possible transport capabilities in the model using high-throughput phenotypic data (i.e. uptake in Biolog plates), enabling our algorithms to select for an optimized growth phenotype and metabolic exchange. We added fructose-6P to our list of metabolites that can be taken up but not secreted. We constrained the model to zero F6P secretion and regenerated all the Figures of the manuscript.

We have also performed a robustness analysis for all exchanged metabolites; evaluating the effect of canceling each exchanged metabolite (zero flux) over the flux of all exchanged metabolites and predicted growth phenotypes. We found that elimination of the F6P exchange does not affect the predicted growth rate of each community member (Figure R1, heatmap), but (as expected) in order to achieve the same growth rates, the model adjusts the exchange of glycolate, NH_4 and serine. Deployed fluxes in the clustergram were normalized using the optimized flux distribution of *i*CZ-Se-Yl(1686) when all exchanges were active. We have now clarified this finding in the main text of our manuscript.

Reviewer #3 (Remarks to the Author):

The authors have responded well to all of my comments, and generally the manuscript seems much improved. In examining the results with the improved clarity, a few questions persist around Figure 4b. Specifically, when the authors refer to E. coli K12 coculture (the orange bars) in this figure, can this coculture be explained further? The text doesn't seem to mention this co-culture at all, just the elongatus coculture. I assume the E. coli K12 coculture involves growing the mutant strain in coculture with the wild-type strain? Is that the case? Also, in Figure 4b, the E. coli coculture model results for cycA don't make sense to me. Here, you are predicting that the monoculture mutant will grow, but the co-culture will not. From what I know about community modeling, this is not expected to happen. When you expand a monoculture model to a coculture model, growth should generally stay similar or go up. It's very unexpected for growth to go away entirely. I could be misunderstanding what the E. coli coculture is, but I don't see why a model that is growing on it's own would suddenly stop growing in co-culture. Is this a mistake? Can this be explained further. Generally methods descriptions for this specific study could use more fleshing out.

We'd like to thank the reviewer for her/his time and effort to review our manuscript in great detail. We have now expanded the interpretation of the results in Figure 4b. Overall, feasible solutions in metabolic models are defined by a “flux solution space”. For communities this is composed by the solution space delimited for each individual metabolic network. In the cases of the knockouts (cycA) in Fig. 4b, we constrained the lower and upper bounds to zero for the reactions associated with the cycA genes. As a result, FBA predicts that growth is not possible under those conditions. When extreme constraints are applied, the optimization results in zero growth and a void flux distribution vector. Usually false negative predictions are associated with limitations to produce the minimum amount of ATP to produce certain amount of resources by the phototrophic partner. False negatives can also be linked to reactions in pathways that need further manual curation in M-models. Overall, CM-models are highly dependent on how comprehensive the individual M-models are. Fig. 4b shows how M-models and CM-models were not able to accurately predict experimentally observed phenotypes. In the last decade different attempts have been reported to curate M-model networks as much as possible (1, 6). This is a very constructive comment and we have now expanded the methods section to describe how to contextualize modeling and experimental outcomes for knockout strains.

References

1. Guzmán GI, Utrilla J, Nurk S, Brunk E, Monk JM, Ebrahim A, Palsson BO, Feist AM. 2015. Model-driven discovery of underground metabolic functions in *Escherichia coli*. Proc Natl Acad Sci 112:929–934.

2. Nicaud J, Madzak C, Van den Broek P, Gysler C, Duboc P, Niederberger P, Gaillardin C. 2002. Protein expression and secretion in the yeast. FEMS Yeast Res 2:371–379.

3. Diner RE, Schwenck SM, McCrow JP, Zheng H, Allen AE. 2016. Genetic manipulation of competition for nitrate between heterotrophic bacteria and diatoms. Front Microbiol 7.

4. Zuñiga C, Li C-T, Yu G, Al-Bassam MM, Li T, Jiang L, Zaramela LS, Guarnieri M, Betenbaugh MJ, Zengler K. 2019. Environmental stimuli drive a transition from cooperation to competition in synthetic phototrophic communities. Nat Microbiol 1–8.

5. Venturelli OS, Carr AC, Fisher G, Hsu RH, Lau R, Bowen BP, Hromada S, Northen T, Arkin AP. 2018. Deciphering microbial interactions in synthetic human gut microbiome communities. Mol Syst Biol 14:e8157.

6. Monk JM, Lloyd CJ, Brunk E, Mih N, Sastry A, King Z, Takeuchi R, Nomura W, Zhang Z, Mori H, Feist AM, Palsson BO. 2017. *i*ML1515, a knowledgebase that computes *Escherichia coli* traits. Nat Biotechnol 35:904–908.

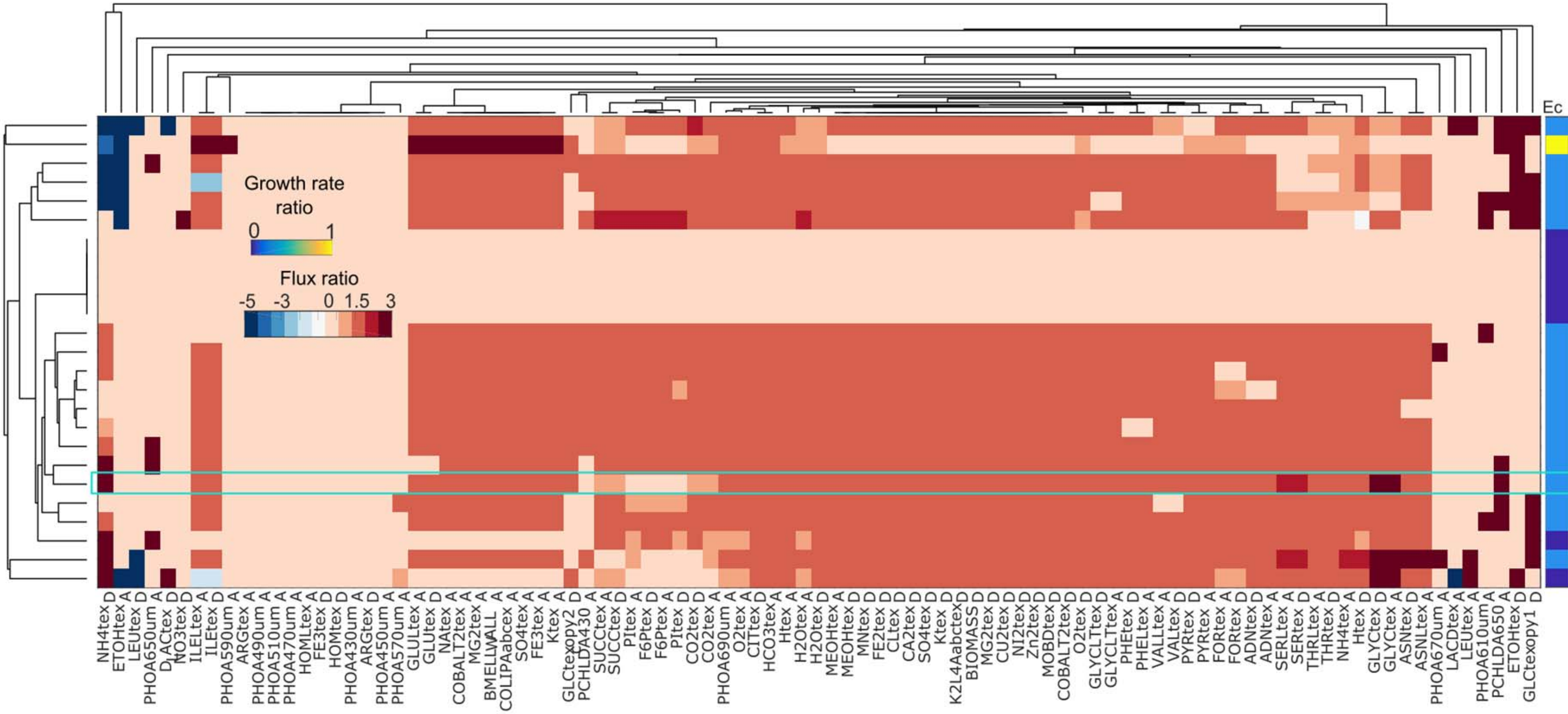

Figure R1| Survival response of the phototrophic community partners while removing metabolic exchange capabilities from community members. Reactions ending with Ec refer to *E. coli* K-12 and reactions ending with Se refer to *S. elongatus*. The growth rate ratio ($gr_{f\rightarrow 0}/gr$) was predicted when the flux of exchanged metabolites was set to zero ($gr_{f\rightarrow 0}$) in turn and compared with standard conditions. The results are displayed in the blue-yellow colored heatmap. Additionally, the effect of each constrain on the flux of all exchanged metabolites was estimated as a flux ratio ($Flux_{met,zero}/Flux_{met,std}$) as shown in the orange-colored clustergram. Transport reactions associated with these metabolites are shown in the vertical axis. Predictions show the rearrangement of metabolic exchanges by the deactivation of each metabolite. Fructose 6P transport elimination was correlated with increased flux through the glycolate, NH4 and serine transporters.

Reviewers' comments:

Reviewer #1 (Remarks to the Author):

The revised manuscript of Zungia et al., is substantially improved in language and presentation. Some of the fundamental insights that are gained through the flux analysis are more clearly stated in the document. Many of my original concerns have been addressed, though I point out a few additional considerations that may further improve reader comprehension below.

Comments:

Line 280 is unclear: ” This prediction was validated experimentally, where the heterotrophic partners improved resistance to S. elongatus knockouts (7-27%) under cocultivation.” This statement appears to say that the authors experimentally validated some set of S. elongatus knockouts that were predicted to be lethal in mono-culture and found that they were rescued in growth in co-culture. This would be a technically impressive feat and would be of considerable interest to the subject of the manuscript, but I can not find any data in Figure 4, or elsewhere, that relates to this statement. (e.g., Fig 4a appears to relate to analysis of simulations, not live cultures). Please revise accordingly.

Related to this point, the manuscript is significantly improved in reader comprehension, but there continues to be some difficulties at various points when the reader is attempting to determine if a given statement or figure panel relates to simulations or experimental data. The authors are to be commended in validating a number of the simulation predictions, but further clarifying when they are referencing simulations vs. experimental (or both) in main text and figure legends would assist the ease of reading. Generally, such ambiguities tend to be clustered towards the later analyses (Figures 3 and 4 and associated Supplemental information)

In the proofs of the Supplemental Information, Figure S2 has text with insufficient resolution to read – even with digital zoom. Likewise, a list somewhere in the manuscript/Supplemental of all of the abbreviations used (e.g., for the various metabolites) and their full names would be helpful.

Minor comments:

Thylakoid transport reactions are lumped together as one of the dominant fluxes that are predicted to constrain community growth parameters by the community models. Because it is unclear what all is included as part of “thylakoid transport” (e.g., line 250), it is hard to know if this might represent a unexpected insight, or merely a more obvious conclusion (i.e., if the model were allowed more active photosynthetic processes, a higher total biomass would be achieved). I was unable to immediately locate all of the reaction fluxes categorized as “thylakoid transport” even in the Supplemental Files. A small clarification in text and/or Supplemental might help the reader to distinguish between these possibilities.

It is curious to see the unusually high discrepancy between anticipated growth rates of S. elongatus CscB in co-culture with Y. liptolytica and the actual growth rate. It appears that this discrepancy is mostly driven by a predicted large growth increase in S. elongatus that is not realized in real cultures. Is there a small set of predicted fluxes from Y. litolytica ◊ S. elongatus that drives the predicted growth enhancement? While beyond the scope of this manuscript, determining heterotroph-to-autotroph fluxes such as these that may be growth promoting could have broader implications for the stability of such synthetic co-cultures.

Reviewer #2 (Remarks to the Author):

The authors have revised the manuscript and applied part of my suggested comments. The additional tables containing previously missing data will definitely increase the visibility and impact of the article. However, I still have some major concerns about the way the results are presented.

Overall, I think the authors made a good job of analyzing the co-culture between cyanobacteria and E. coli. They combined modeling and experiment and the predictions were validated in most cases. One main conclusion of this work is how complex communities are and how with our current technical limitations we are unable to properly understand metabolite exchange. This modeling approach can become a good tool to explore this or even be used to improve our technical capabilities. However, it is clear that the experimental validations only explain partially the results of the model. That must be taken into account and, unfortunately, narrow down the applications of such a model, as if for example, it is used in order co-cultures, new experimental validations will be required, and for some organisms, there are no resources available like the knockout collections.

All this make this paper a valuable piece of work of high quality for the main co-culture investigated in detail and the major conclusions should refer to this. On the contrary, when adding other co-cultures, no studied in detail and with partial or weak explanations for many obtained results, the quality of the manuscript decreases as well as the generalist conclusions.
I recommend the authors re-organize the manuscript and present it in a different way taking to a side/ supplementary the other co-cultures. The current format makes the manuscript uncomplete and it makes the reader wonder the general validity of the conclusions.
Even when the authors claim in the response to reviewers that they have explained well why they only move forward with one co-culture, this is in my opinion neither achieved nor justified in the current version. For example, the abstract is still very misleading as by reading it you still have the impression that this is a generic article and that all the experiments have been done for the 4 co-cultures mentioned there. Again, starting by saying that this article is about 4 co-cultures is impairing the quality of the work, as most of the article is about 1. The article would benefit with a clear linear story on the chosen co-culture and presenting, if so, the other three as an additional or side study.

Figure 2B and the associated text (146-154) is a bit random, it is not clear why only those metabolites where selected, especially now that the reference to bioproduction as been removed. I am not sure this fit in the main text, as the conclusions of the study are also not adding new knowledge. I would move this out to supplementary as take the reader off the main conclusions and results of the work.

The fact that the work has been done in flask (Batch), in my opinion, make the work more difficult to replicate. How did the authors calculate growth rate? Looking at the curves, the growth often looks more linear than exponential.

The authors used Yarrowia Po1g, but this strain is auxotroph so it should not grow on the media where it is shown growth (it is unable to produce some vitamins and Uracil, among others)(probably one of those has been recovered while expressing the SUC gene, but what about the others?). This strain suffered several rounds of mutations, which makes it genome different from the original wild type strain W29. What is the genome used to generate the model used here? Maybe some of these differences are responsible for the inaccuracies between the prediction and the experiments.

Regarding the secretion of fructose 6P, I appreciate it can be taken up, however, it is known that metabolism has evolved to differentially transport things in and out, in order to keep valuable molecules in (such fructose, whose excess is not released to the media but used to build sugar storage molecules). This particular example makes me wonder if the general rule assumed by the authors where "CM-models assume that when a metabolite can be consumed, it can be potentially excreted as well" is responsible for inaccuracies and predictive errors. Such a rule should be reconsidered taking into account biological pieces of evidence and our knowledge on transporter and metabolism. This can be easily done by controlling the boundaries of the transport reaction to take place only in the right direction.

Reviewer #3 (Remarks to the Author):

The authors have responded well to all of my comments, and generally the manuscript seems much improved. In examining the results with the improved clarity, a few questions persist around Figure 4b. Specifically, when the authors refer to E. coli K12 coculture (the orange bars) in this figure, can this coculture be explained further? The text doesn't seem to mention this co-culture at all, just the elongatus coculture. I assume the E. coli K12 coculture involves growing the mutant strain in coculture with the wild-type strain? Is that the case?

Also, in Figure 4b, the E. coli coculture model results for cycA don't make sense to me. Here, you are predicting that the monoculture mutant will grow, but the co-culture will not. From what I know about community modeling, this is not expected to happen. When you expand a monoculture model to a coculture model, growth should generally stay similar or go up. It's very unexpected for growth to go away entirely. I could be misunderstanding what the E. coli coculture is, but I don't see why a model that is growing on it's own would suddenly stop growing in co-culture. Is this a mistake? Can this be explained further. Generally methods descriptions for this specific study could use more fleshing out.

Response NCOMMS-19-28644B

Reviewer #1 (Remarks to the Author):

In their revised manuscript by Zuniga et al., the questions and comments I had previously noted have all been sufficiently resolved. Furthermore, the manuscript continues to be more intuitively organized and more readily understood. I therefore raise only editorial suggestions for this latest round of review: all of these are relatively minor and some are more stylistic opinion. These suggestions should be viewed as that (i.e., not requirements).

We'd like to thank the reviewer for her/his time and effort taken to review our paper. We have addressed all the editorial comments raised during the latest revision.

Minor comments:
Line 82: Consider deleting “the” in “the heterotrophic E. coli….”

We changed the text. This sentence now reads: ”Thus, CM-models for four synthetic communities based on the sucrose-secreting *S. elongatus* in pairwise combination with *E. coli* (strains K12 and W), *B. subtilis* str 168, and *Y. lipolytica* Po1g were reconstructed.”

Introduction (generally): In a prior round of reviews, it was mentioned that S. elongatus cscB strains have now been used for the construction of autotroph/heterotroph co-cultures with many different heterotrophic microbes, across a number of different labs. While not comprehensive, there are adequate citations to this literature in the current manuscript. However, for those not already familiar with the system, the logic/rationale for the engineered (and one of the dominant) fluxes from S. elongatus to heterotroph (that is, the secretion of sucrose as the carbon source in the media) is still a little oblique in the text. Consider if adding a sentence (or revising an existing sentence) might make the core rationale of this co-culture platform easier to follow.

We thank the reviewer for this valuable comment. We have included sentences (marked in yellow) further describing the core of our system. The paragraph now reads: ”Such phototrophic communities can exist in the harshest environments on Earth facing desiccation, nutrient starvation, salinity, temperature extremes, and high UV radiation[5]. This ability to survive extreme conditions is due, in part, to division of labor and subsequent interactions between members of the community. Photoautotrophic members, classically either cyanobacteria or eukaryotic algae, convert CO_2 into organic carbon for growth and maintenance of the heterotrophic partner(s). In turn, the heterotrophs provide additional CO_2, protection from environmental factors and predation, and often, a diverse array of metabolites produced by secondary metabolism[6]. Exchange of these metabolites can sustain the heterotrophs under conditions devoid of any organic carbon source. To date, understanding, engineering and determining viable cultivation conditions for natural phototrophic communities remains challenging[7].”

Line 124: The statement here seems to indicate that S. elongatus has a leucine auxotrophy, which is not the case. Please revise text accordingly.
Good catch - we fixed the text. The paragraph now reads as: “These expectations were validated by experiments with the exception of *Y. lipolytica-S. elongatus* communities in which *S. elongatus* did not see a boost in growth but instead exhibited diminished growth, despite our simulations showing active exchange of isoleucine and other amino acids.”

Line 133: Just a note here: a few other groups have identified a failure for B. subtilis to maintain growth in co-culture at higher densities of S. elongatus. This may be due to negative feedback – perhaps because of a higher sensitivity of B. subtilis to hyperoxia and/or ROS.

We thank the reviewer for this comment. We now discuss the effects of hyperoxia and reactive oxygen species on *B. subtilis* phenotypes (Line 352-353). This sentence now reads: ”Meanwhile, SPCs containing *B. subtilis* or *Y. lipolytica* did not grow experimentally as predicted, possibly due to the high sensitivity of amino acids to folding events of metal transporters[39], deactivation of hexose transporters in *Y. lipolytica*[40], as well as to the presence of reactive oxygen species[8,9] potentially affecting growth of *B. subtilis.*”

Lines 188-192: There is reference to a “second-highest” SPC with regard to flux exchanges. A slight point of refinement here is if the K-12 SPC is the “first-highest”? Also, I presume this discussion is referring to the total integration of all predicted exchange fluxes?
We thank the reviewer for pointing this out. We have now rephrased these sentences, explaining that the ranking was defined based on total carried flux thought the shared metabolite pool.

Line 201: Can you really experimentally state that the S. elongatus/K-12 SPC is the co-culture with the highest rate of exchange fluxes? This seems possible to quantify and defend in a simulation, but - without exhaustive measurements of exchange fluxes in real experiments – this seems very difficult to defend in vivo.
We thank the reviewer for pointing this out and we apologize for this potentially misleading statement. We have now reworded this paragraph, clarifying that the coculture *S. elongates - E. coli* K-12 exhibit the highest growth phenotypes rather than metabolic exchange.

Reviewer #2 (Remarks to the Author):

The authors have improved the manuscript and now the whole story is easier to follow and read. In addition, the authors replied to my questions and justified some of the discrepancies we had. They also have repeated the results of the models according to my comments and the comments from other reviewers.

We thank the reviewer for her/his time and effort to review our manuscript and to acknowledge our efforts to address previous concerns.

I would like to suggest a few things in relation to this last version.
-It is very important that the supplementary material also includes all the models with enough documentation so others can use it for their own purposes. The same for all the experimental data on metabolite exchange and production. That will add a good value to the article.

We thank the reviewer for this comment. The tables for all experimental data, models and documented code is now included in the supplementary material and it is also available at https://github.com/cristalzucsd/SyntheticMicrobialCommunities.

-It is still not very clear why the best model is the E coli one. The experiments seem to suggest that the growth of B. subtilis is much faster and better, so more useful for co-cultures.

We thank the reviewer for his comment. *B. subtilis* achieves remarkably higher growth phenotypes under monoculture conditions. However, the same phenotypes were not observed under coculture conditions, as shown in Fig. 1a. Experimental observations also pointed to highly variable phenotypes during co-cultivation, showing that this coculture was not stable. Changing growth phenotypes can be potentially associated with high sensitivity of amino acids to folding events of metal transporters (Wakeman et al., 2014) as well as to the presence of reactive oxygen species (Hays et al., 2017; Li et al., 2017) affecting the growth of *B. subtilis.* We now included these possibilities in the discussion section. This would clearly be a topic of future research.

-line 33, I would start by: "For this co-culture.." or would specify somehow that those experiments were done only in the E. coli pair, which is not obvious as it is written now.

We changed the text accordingly.

-In the title I am not convinced about the word, Designing. The work does not really provide a design guide at this stage that can be used to create new co-cultures. I would substitute it for Modelling.

Fixed in text. The new title reads as: Synthetic microbial communities of heterotrophs and phototrophs facilitate sustainable biotechnology.

In relation to that, what do the authors mean by a workflow for the rational design in line 37? I don't see clear that the paper provides such a workflow or guide for design.

We reworded this part, it now reads as “modelling framework” instead of “workflow”.

-line 146-158. I appreciate the clarification on why those metabolites where selected. However, I still have some questions related to these experiments, as the value of this part is not really clear to me. Is this a way to see if the production of those metabolites is in the metabolic network? Is there any difference between the monocultures and co-cultures? also, have these metabolites been detected experimentally at all? have their production been reported in the literature? if so, how the production level compare? to me, these numbers are extremely low to present any kind of viable production process. Also, I don't find clearly in the methods how these values were calculated, which were the constraints in the model and the objectives functions? This part is to me not very valuable and not supported enough to be presented in the main text of a Nature communications article.
We thank the reviewer for this comment and we apologize for the lack of background in this section. Results in Fig. 2 have the goal to demonstrate the topological potential of community metabolic networks (CM-modes) regarding bioproduction in comparison with single organism networks (M-models). Usually, to optimize the synthesis of a certain metabolite, complex algorithms and knockout simulations are needed when using M-models. In some cases a combination of M-models with reaction kinetics is applied (Khodayari et al., 2015). On the other hand, CM-models expand the network topology and highly increase the feasibility of the model. These network capabilities achieve feasible solutions while simulating increased metabolite productivities as a result of the metabolic-flux-adjustment of the community members. We selected added value chemicals (e.g. methanol, formaldehyde, ethanol, butanal, and succinate) as show cases. Simulations about those are only some examples of all possible metabolites that can be produced in a coculture framework. We compared the productivity for communities with those reported by engineered monocultures and found that cocultures were more competitive to produce succinate but not ethanol (Supplementary Table 3). The reasons for reduced productivity for ethanol could be manifold (e.g. toxicity) and are not the focus of this study. However, the ability of cocultures to expand the potential of metabolites being produced is very intriguing. Keep in mind that these are heterotrophs without any genetic modification and that certain shortcomings, such as ethanol sensitivity, could easily be overcome in the future. Engineering strains for optimized production in co-cultures would likely achieve higher productivities. Our focus here was to determining community network capabilities and not so much on improving yield. We now expanded Supplementary Table 3 to show a comparison among yields for all SPCs and metabolites under monoculture and coculture conditions. We have also included bioproduction experimental evidence for monoculture conditions when available, predicting that cocultures can achieve higher productivities than optimized strains under monoculture. Additionally, all CM-models generated during this research are publically available and will facilitate significantly the identification of suitable network states to improve yields.

In addition, we now included background about these simulations in the results section. We also expanded the methods section and Supplementary Table 3, including details about how the

simulations were performed and experimentally determined productivities under monoculture condition of our selected metabolites.

Editor: Reviewer #2 provides his/her comments on your responses to Reviewer #3's previous suggestions in Remark to Editor section.

Below is the summarized version of the report: One of the concerns of the Reviewer ## was that some of the results in that figure do not make sense, in particular the one about cycA. The response of the authors does not respond well to the question of Reviewer 3, as the question of Reviewer 3 is why if the model of the monoculture predicts growth the co-culture does not grow. the opposite result could be understood by the synergies of the co-culture but when the individual monocultures can grow it is difficult to know why the model predicts that the co-culture cannot grow. The experimental results are however more logical. Is this a flaw in how the model operates? This has not been really responded and should be addressed.

Constraint-based models depend on model structure (e.g. network topology and connectivity) and the constraints that are applied (e.g. uptake and secretion rates, lower and upper reaction boundaries) to simulate phenotypes. When extreme constraints are applied, the solution space gets reduced and optimization results are unfeasible (zero growth with a void flux distribution vector). Every knockout simulation layers an additional constraint, which can affect simulation outcomes. In the case of the knockout cycA the reactions associated with this gene are extremely important for the network, making it impossible for the model to simulate a flux distribution and resulting in a false negative outcome. This outcome can be also understood as a flaw of the model. However, as the reviewer pointed out experimental validation showed that this gene maintained its growth phenotype under coculture conditions.

References

Hays, S.G., Yan, L.L.W., Silver, P.A., Ducat, D.C., 2017. Synthetic photosynthetic consortia define interactions leading to robustness and photoproduction. J. Biol. Eng. 11, 4. https://doi.org/10.1186/s13036-017-0048-5

Khodayari, A., Chowdhury, A., Maranas, C.D., 2015. Succinate overproduction: A case study of computational strain design using a comprehensive *Escherichia coli* kinetic model. Front. Bioeng. Biotechnol. 2. https://doi.org/10.3389/fbioe.2014.00076

Li, T., Li, C.-T., Butler, K., Hays, S.G., Guarnieri, M.T., Oyler, G.A., Betenbaugh, M.J., 2017. Mimicking lichens: incorporation of yeast strains together with sucrose-secreting cyanobacteria improves survival, growth, ROS removal, and lipid production in a stable mutualistic co-culture production platform. Biotechnol. Biofuels 10, 55. https://doi.org/10.1186/s13068-017-0736-x

Wakeman, C.A., Goodson, J.R., Zacharia, V.M., Winkler, W.C., 2014. Assessment of the requirements for magnesium transporters in *Bacillus subtilis*. J. Bacteriol. 196, 1206–1214. https://doi.org/10.1128/JB.01238-13

Reviewer #2 (Remarks to the Author):

The authors addressed all my comments.

The last remark is that the tile has been changed back to include biotechnology what is misleading as the article is not related to biotechnology. I would suggest to just use the same title as in the previous revision but removing the word designing. therefore it would be:
Synthetic microbial communities of heterotrophs and
phototrophs facilitate sustainable growth

REVIEWERS' COMMENTS:

Reviewer #2 (Remarks to the Author):

The authors addressed all my comments.

The last remark is that the tile has been changed back to include biotechnology what is misleading as the article is not related to biotechnology. I would suggest to just use the same title as in the previous revision but removing the word designing. therefore it would be: Synthetic microbial communities of heterotrophs and phototrophs facilitate sustainable growth

A: we thank the reviewer for suggesting a new title. We have decided to use his/her recommendation.
